# Mandipropamid as a chemical inducer of proximity for in vivo applications

**DOI:** 10.1038/s41589-021-00922-3

**Published:** 2021-12-21

**Authors:** Michael J. Ziegler, Klaus Yserentant, Valentin Dunsing, Volker Middel, Antoni J. Gralak, Kaisa Pakari, Jörn Bargstedt, Christoph Kern, Annett Petrich, Salvatore Chiantia, Uwe Strähle, Dirk-Peter Herten, Richard Wombacher

**Affiliations:** 1 Institute of Pharmacy and Molecular Biotechnology, Heidelberg University, Heidelberg, Germany; 2 Department of Chemical Biology, Max Planck Institute for Medical Research, Heidelberg, Germany; 3 Institute of Physical Chemistry, Heidelberg University, Heidelberg, Germany; 4 Faculty of Biosciences, Heidelberg University, Heidelberg, Germany; 5 Institute of Cardiovascular Sciences & School of Chemistry, College of Medical and Dental Sciences, University of Birmingham, Birmingham, UK; 6 Institute of Biology and Biochemistry, University of Potsdam, Potsdam, Germany; 7 Institute of Biological and Chemical Systems (IBCS)—Biological Information Processing (BIP), Karlsruhe Institute of Technology (KIT), Eggenstein-Leopoldshafen, Germany; 8 Centre of Membrane Proteins and Receptors (COMPARE), Universities of Birmingham and Nottingham, Midlands, UK

**Keywords:** Chemical tools, Synthetic biology, Biophysical chemistry

## Abstract

Direct control of protein interactions by chemically induced protein proximity holds great potential for both cell and synthetic biology as well as therapeutic applications. Low toxicity, orthogonality and excellent cell permeability are important criteria for chemical inducers of proximity (CIPs), in particular for in vivo applications. Here, we present the use of the agrochemical mandipropamid (Mandi) as a highly efficient CIP in cell culture systems and living organisms. Mandi specifically induces complex formation between a sixfold mutant of the plant hormone receptor pyrabactin resistance 1 (PYR1) and abscisic acid insensitive (ABI). It is orthogonal to other plant hormone-based CIPs and rapamycin-based CIP systems. We demonstrate the applicability of the Mandi system for rapid and efficient protein translocation in mammalian cells and zebrafish embryos, protein network shuttling and manipulation of endogenous proteins.

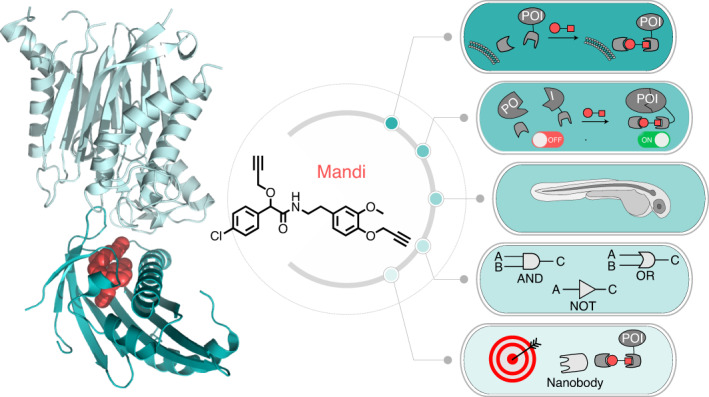

## Abstract (web summary)

The agrochemical mandipropamid (Mandi), which induces dimerization of a variant of the abscisic acid receptor, has been developed as a new chemical inducer of proximity for cellular and organismal applications.

## Main

**Fig. 1:**
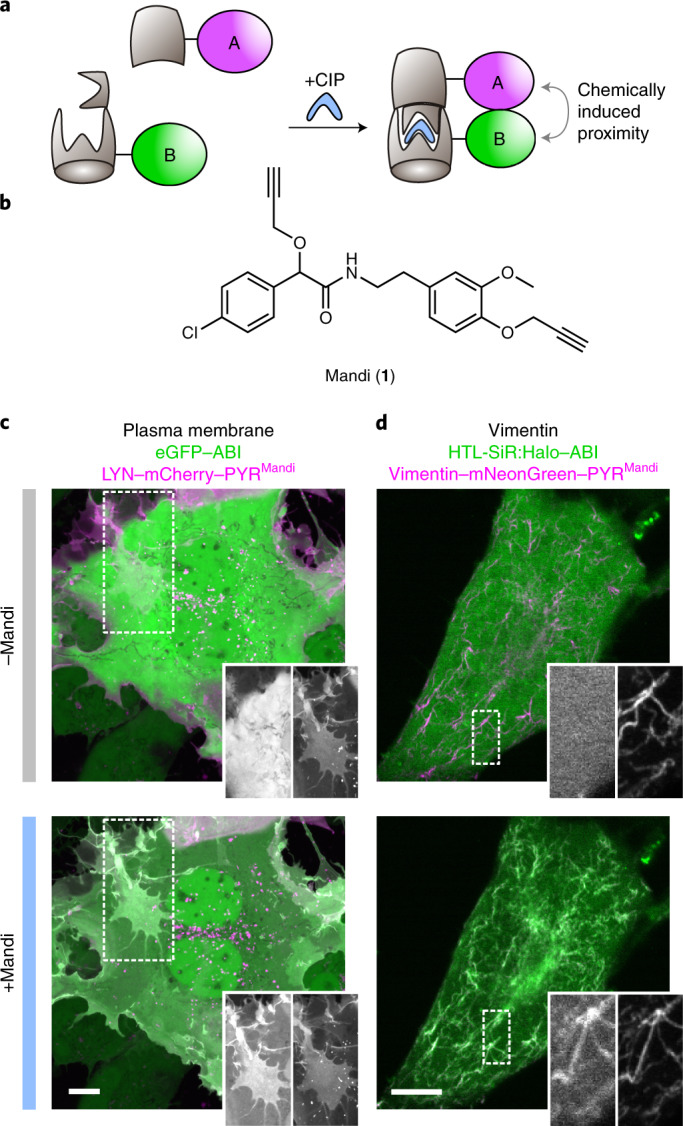
Mandi, a new CIP. **a**, Chemically induced protein proximity to control interactions between proteins of interest A and B. **b**, Chemical structure of Mandi. **c**, Live-cell confocal microscopy of COS-7 cells cotransfected with pLYN-mCherry-PYR^Mandi^ and enhanced green fluorescent protein peGFP-ABI before and 2 min after Mandi addition (100 nM); data are representative of seven cells. **d**, Live-cell confocal microscopy of COS-7 cells transfected with pvimentin-mNeonGreen-PYR^Mandi^-IRES-Halo-ABI and labeled with HaloTag ligand-SiR (HTL-SiR). Images were acquired before and 5 min after Mandi addition (50 nM); data are representative of 20 cells. The scale bars in **c** and **d** represent 10 µm. See Extended Data Fig. 2 for single-channel images.

Protein proximity is a key regulatory mechanism in cellular processes, including metabolic pathways and cellular signaling, which are essential to sustain cellular integrity and to organize cellular response. Tools used to investigate and manipulate protein proximity have to meet a range of demanding requirements, such as fast dose–response, high efficiency and spatial control. At the same time, they should not interfere with the process under study or other cellular processes and they should not be cytotoxic. CIPs are small, drug-like molecules that induce protein proximity by mediating interactions between specific receptor and receiver domains (Fig. 1a) and have been widely used in biology^1^. Different CIP systems have been successfully used to control protein proximity in various applications, such as signal transduction^2–4^, protein translocation^5^, degradation^6^ and aggregation^7^. Thus, they hold great potential for future drug development by specific control of metabolic pathways and signaling cascades^1^. The immunosuppressant rapamycin (5) is currently the best-studied CIP and has become well established to precisely manipulate cellular protein interactions^8,9^. However, while rapamycin was shown to be cell permeable and applicable in vivo, unwanted interaction with its endogenous target mammalian target of rapamycin (mTOR) can complicate the application of rapamycin as a CIP. Rapamycin analogs, so-called rapalogs, have been shown to be less toxic^10^, but their complex chemical structure can make them difficult to access. Therefore, there is high interest in novel CIPs that are orthogonal, that are chemically easily accessible and that exhibit excellent cell and tissue penetration behavior.

**Extended Data Fig. 1:**
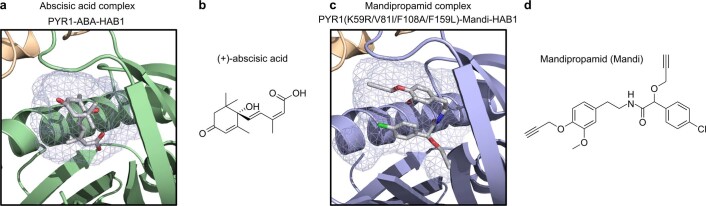
Crystal structures of CIP-bound receptor-receiver complexes for abscisic acid and mandipropamid. PYR1-HAB1 complex bound by ABA. b, Molecular structure of (+)-abscisic acid. c, PYR1(K59R/V81I/F108A/F159L)-HAB1 complex bound by mandipropamid. No crystal structure for hextuple mutant PYR^Mandi^ is available to date. d, Molecular structure of mandipropamid (Mandi). Crystal structures obtained from pdb entries 3JRQ and 4WVO and based on previous reports in the literature^12,15^.

Phytohormone-based CIP systems have received significant attention over the past years because they make use of plant proteins, which do not occur in the animal kingdom and are therefore fully orthogonal to processes in mammalian cells. Gibberellic acid (GA_3_; 6) as well as abscisic acid (ABA; 3) induce protein–protein interactions following ligand binding to regulate plant growth^11^ or stress resistance^12^ in plants. Both, GA_3_ and ABA in combination with their dimerization domains gibberellin-insensitive dwarf protein 1/gibberellic acid insensitive (GID1/GAI) and pyrabactin resistance-like (PYL)/ABI, respectively, have been used as CIP systems with times to effect in the range of minutes^13,14^. Recently, engineered ABA receptors have been reported for agrochemical control of water use in plants^15^. The genetically modified receptors do not respond to the phytohormone ABA but to the agrochemical Mandi (1), a fungicide extensively used in agriculture (Fig. 1b). A hextuple mutant PYR^Mandi^ of the ABA receptor PYR1 was identified that specifically binds Mandi^15^, replacing the natural ABA response in plants (Extended Data Fig. 1). On the basis of this finding, we decided to evaluate the PYR^Mandi^ mutant of the ABA receptor with Mandi as a CIP in mammalian systems, analogous to the use of the ABA system in previous works. The simple chemical structure of Mandi enables cheap and easy access to the molecule, and its low polarity also suggests very good cell and tissue penetration properties, which is of particular importance for in vivo applications. We demonstrate that the combination of the dimerization domains PYR^Mandi^ and ABI with Mandi as a CIP is a highly efficient CIP system to induce protein proximity in cellular systems as well as in vivo in an unrivaled fast and acute manner. Further, we show how the Mandi system can be used in conjunction with nanobodies to manipulate endogenous proteins. Finally, we demonstrate that the combination of the ABA and Mandi CIP systems allow for controlled and efficient shuttling of proteins between different cellular locations.

## Results

### Characterization of Mandi as a new CIP

We hypothesized that, like the GA_3_ and ABA systems, Mandi and the respective receptor PYR^Mandi^ can be used as a CIP in mammalian cells. With its simple molecular structure, Mandi is readily available either by chemical synthesis^16^ or commercially (Supplementary Table 1) as a pure compound. We therefore propose Mandi as an attractive candidate to overcome current limitations of CIP systems to leverage these tools for in vivo applications.

**Fig. 2:**
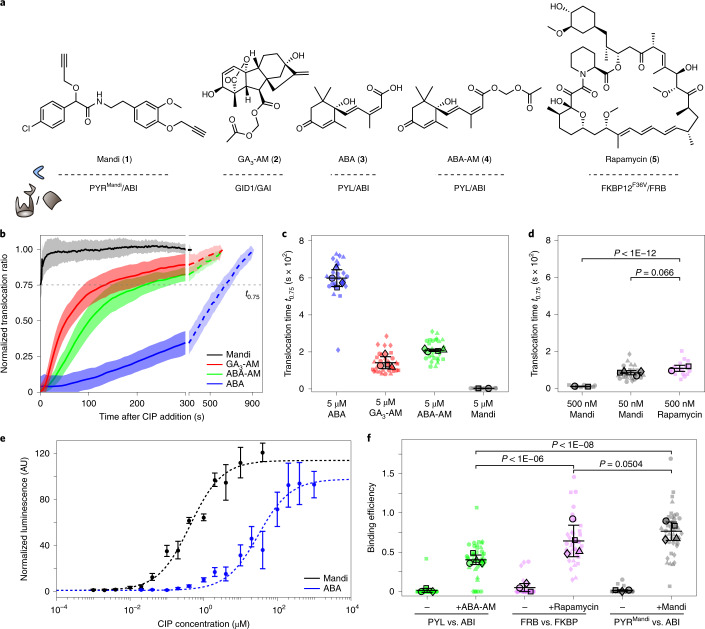
Quantitative comparison of the new Mandi system with existing CIP systems. **a**, Chemical structure of different CIP systems and their respective receptor and receiver domains. **b**, Single-cell translocation kinetics of the cytosolic receiver domain to the receptor domain localized on mitochondria. Trajectories were normalized to ratios before CIP addition and after translocation was completed. Data represent mean (line) ± s.d. (shaded region). See Supplementary Table 2 for number of cells and experiments. CIPs were injected at a 5 µM final concentration at *t* = 0 s. The translocation time, *t*_0.75_, is indicated by the dashed line. See Supplementary Fig. 4 for single-cell translocation trajectories and Supplementary Fig. 5 for averaged trajectories from experiments with reduced Mandi concentrations. **c**,**d**, Translocation times for different CIPs and CIP concentrations. Small symbols represent individual cells, and large symbols represent means from experiments. See Supplementary Table 2 for the number of cells and experiments for each condition. The means ± s.d. across experiments are indicated by error bars. **e**, Dose–response (median ± s.d.) of ABA- or Mandi-induced luciferase expression in COS-7 cells after 24 h of incubation. Four (7) samples from three (4) independent experiments for Mandi (ABA-AM). **f**, Binding efficiencies from RSICS experiments before and after CIP addition at a 500 nM final concentration. Lines indicate mean ± s.d., and symbols are as described in **c**. Conditions were compared using a two-sided unpaired *t*-test with Welch’s correction. Source data

**Extended Data Fig. 2:**
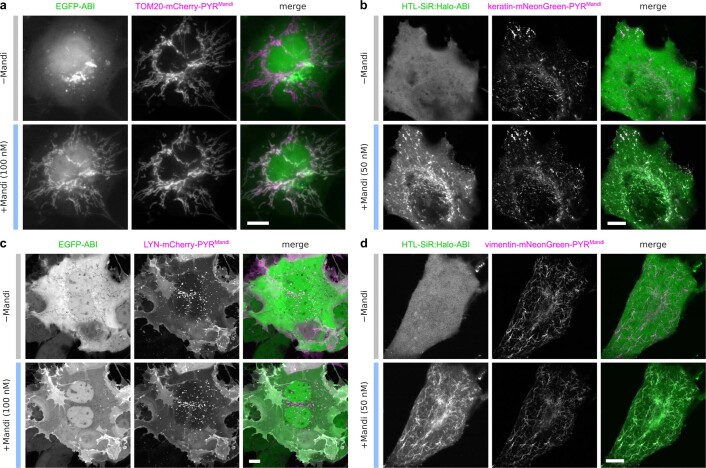
Mandi-induced protein translocation to different subcellular targets. Live-cell fluorescence microscopy of COS-7 cells transiently transfected with receiver domains targeted to subcellular targets and cytosolic receiver domain. Images were acquired by epi- (a) or confocal (b-d) fluorescence microscopy before and 2 min (c) or 5 min (a,b,d) after addition of Mandi at indicated final concentrations. Transfection with pTOM20-mCherry-PYR^Mandi^-IRES-EGFP-ABI (a), pKeratin-mNeonGreen-PYR^Mandi^-IRES-Halo-ABI (b), pLYN-mCherry-PYR^Mandi^ and peGFP-ABI (c), Vimentin-mNeonGreen-PYR^Mandi^-IRES-Halo-ABI (d). Halo-ABI was labeled with HTL-SiR. Scale bars 20 µm. Representative data for 6–20 cells in 1–2 independent experiments.

To test if Mandi can induce protein proximity in mammalian cells, we used a colocalization assay based on fluorescently labeled fusion proteins. We expressed the receptor domain PYR^Mandi^ fused to different intracellular proteins with characteristic localization and the receiver domain ABI as a cytosolic protein. Addition of Mandi resulted in rapid colocalization at the designated targets in all tested cell lines (Fig. 1c,d and Extended Data Fig. 2). While addition of Mandi to a final concentration of 1 µM resulted in efficient colocalization within seconds, colocalization using 100 nM Mandi was complete within 1 min. At 10 nM, colocalization was still detectable after 4 min, although less efficient (Supplementary Fig. 1 and Supplementary Videos 1 and 2). To quantitatively show the superior performance of a Mandi-based CIP system over existing approaches, we performed a direct comparison with other phytohormone-based CIP systems as well as the most commonly used CIP rapamycin (Fig. 2a). We used the acetoxymethyl (AM) ester-modified derivative of GA_3_ (GA_3_-AM; 2) with improved membrane permeability^13^. For ABA we evaluated both, the commonly used unmodified carboxylic acid^14^ and the so far not-reported corresponding AM ester (ABA-AM; 4). To determine the time to effect for each CIP, we extracted the recruitment kinetics for a cytosolic receiver to its corresponding receptor domain targeted to the outer mitochondrial membrane using a TOM20 fusion protein. Using an automated epifluorescence microscopy platform with integrated liquid handling, we performed time-lapse imaging after CIP addition and used a machine learning approach for automated cell segmentation and subsequent intensity readout^17,18^ (Supplementary Figs. 2 and 3). This allowed us to measure times to effect for different CIP systems across a large number of cells for a quantitative comparison. Addition of ABA, ABA-AM and GA_3_-AM at a 5 µM concentration resulted in receiver recruitment to mitochondria measured as the translocation ratio *t*_0.75_ (the time at which translocation to mitochondria reached 75% of maximum; see Methods) values within 10 ± 0.8, 3.5 ± 0.1 and 2.4 ± 0.5 min (mean ± s.d.), respectively (Fig. 2b,c). By contrast, translocation induced by Mandi or rapamycin at the same concentration occurred too fast to be resolved in this assay. Therefore, we compared the translocation kinetics of Mandi and rapamycin at 500 nM CIP concentration (Fig. 2d and Supplementary Fig. 6) and observed a tenfold faster recruitment for Mandi (*t*_0.75_ of 10.1 ± 1.7 s) than rapamycin (*t*_0.75_ of 107.9 ± 16.4 s). Remarkably, at a 50 nM concentration, translocation induced by Mandi was still >1.2 times faster than with rapamycin at 500 nM (Fig. 2d). Although previous works successfully used ABA as a CIP without AM modification, our results also showed that a significant rate enhancement, presumably due to improved cell permeability, can be achieved for the ABA CIP system using the AM ester ABA-AM.

Previous studies showed that the high-affinity rapamycin system essentially functions as an ‘on/off switch’, whereas abscisic acid shows a dose–response profile with a large linear range, allowing concentration-dependent control of induced protein proximity^14^.

**Extended Data Fig. 3:**
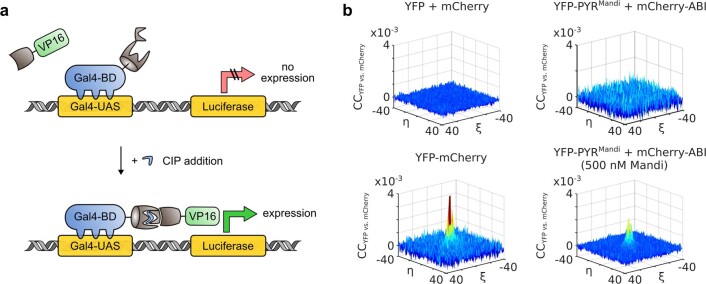
Quantitative characterization of Mandi via control of transcriptional activation and RICS. **a**, Schematic illustration of CIP-induced luciferase expression related to Fig. 2e. **b**, Representative cross-correlation functions of YFP and mCherry signal in COS-7 cells transiently transfected with indicated constructs. Left: negative (YFP + mCherry) and positive (YFP-mCherry fusion) controls. Right: mCherry-ABI vs. YFP-PYR^Mandi^ before (top) and after (bottom) addition of 500 nM Mandi. Binding efficiency for shown mCherry-ABI vs. YFP-PYR^Mandi^ after Mandi addition cross-correlation: 73%.

**Extended Data Fig. 4:**
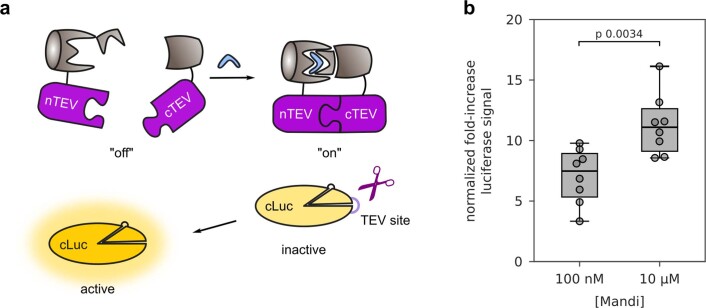
Chemically induced reconstitution of split proteases. **a**, Schematic illustration of CIP induced reconstitution of TEV protease followed by proteolytic activation of cyclic Luciferase. **b**, Normalized enhancement of cLuc activity induced by proteolytic activation at different Mandi concentrations. Box extends from 25^th^ to 75^th^ percentiles, whiskers from min to max, bar represents median. Conditions were compared using two-sided unpaired t-test with Welch’s correction. Representative data from one experiment of two independent experiments. Source data

To evaluate the dose–response dependency of Mandi-induced proximity, we used a gene expression assay with luciferase readout. Induced proximity between the yeast Gal4 DNA-binding domain (Gal4BD), preassociated with the Gal4 upstream activation sequence (Gal4UAS) and the viral VP16 transactivation domain (VP16), induces transcription of luciferase (Extended Data Fig. 3a). Similar to abscisic acid, a dose-dependent response of luciferase expression strength was observed for Mandi (Fig. 2e), which is advantageous for precise control of protein proximity. The comparison of the half-maximal effective concentration (EC_50_) showed an approximately 72-fold lower EC_50_ value for Mandi (0.43 ± 0.17 µM; mean ± s.d.) than ABA (30.8 ± 15.5 µM; 95% confidence interval (95% CI)) and highlights the increased efficiency of Mandi. The tunability of Mandi-induced proximity could further be shown by dose-responsive activation of luciferase in a split tobacco etch virus (TEV) protease assay^19^ (Extended Data Fig. 4).

To further characterize the interaction efficiency between receiver and receptor domains in the new Mandi CIP system, we measured the relative amount of receiver bound to receptor in the absence and presence of CIPs using raster image correlation spectroscopy (RICS)^20^. After transient expression of cytosolic receiver and receptor domains fused to spectrally different fluorescent proteins, we determined the interacting fraction by computing the cross-correlation functions (CCF) between spectral channels and normalizing the obtained CCF amplitudes to controls with or without constitutive interaction between fluorophores^21^ (Extended Data Fig. 3b). Mandi and rapamycin showed similar interacting fractions of their respective receptor and receiver domains after stimulation with 500 nM CIP (Mandi, 77 ± 12%; rapamycin, 71 ± 3%; mean ± s.d.; Fig. 2f), whereas for ABA-AM, at the same concentration, only 41 ± 6% interaction was observed.

### Mandi-induced protein dimerization in vivo

While CIP technologies for in vivo application are of broad interest, their translation from cellular systems to higher organism(s) is hampered by demanding requirements for cell permeability, low toxicity and a favorable pharmacokinetic profile. Rapamycin is both toxic and immunosuppressive and, consequently, of limited use for applications in living organisms because of its narrow therapeutic window^22^. Rapalogs, which were developed to overcome these limitations, have been successfully used in vivo, for example, for caspase activation in mice^23^. However, due to their highly complex chemical structures, they are costly alternatives that forfeit some of the activity of rapamycin^24^. Heterobifunctional CIPs have been successfully applied in *Xenopus* embryos^25^, but the bifunctionality with different binding characteristics can lead to saturation of the binding sites at high concentration with formation of unproductive protein small-molecule conjugates, which has been described as the hook effect in the context of proteolysis-targeting chimeras (PROTACs)^26,27^. The use of GA_3_ and ABA as CIPs in a xenograft mouse model has been recently demonstrated but required intraperitoneal injection of the CIPs^23^, presumably because of limited cell permeability of both CIPs. As Mandi possesses drug-likeness with very low toxicity in mammals^28^ and shows fast and efficient induction of protein interactions, we hypothesized that it may be ideally suited for in vivo applications.

**Fig. 3:**
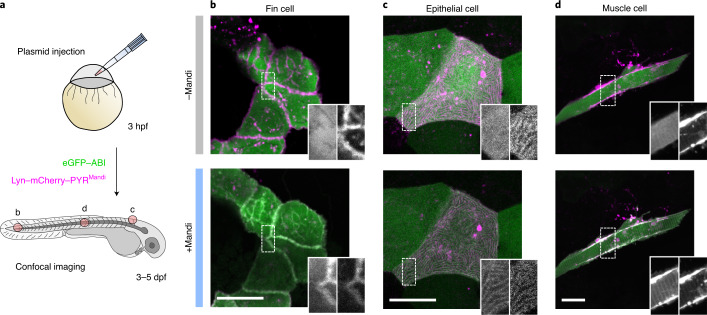
Protein translocation in living zebrafish embryos. **a**, Schematic illustration of workflow for in vivo application in zebrafish embryos. Fertilized eggs were injected with vectors for LYN–mCherry–PYR^Mandi^ or TOM20–mCherry–PYR^Mandi^ and eGFP–ABI expression, resulting in mosaic expression of target proteins at 3–5 dpf. **b**–**d**, Confocal microscopy images of different cell types (fin cells (**b**), epithelial cells (**c**) and muscle cells (**d**)) in living zebrafish embryos expressing receiver and plasma membrane-localized receptor domains before and 10–20 min after addition of 500 nM Mandi are shown. Data are representative of ≥three independent experiments for each cell type; scale bar, 40 µm.

**Extended Data Fig. 5:**
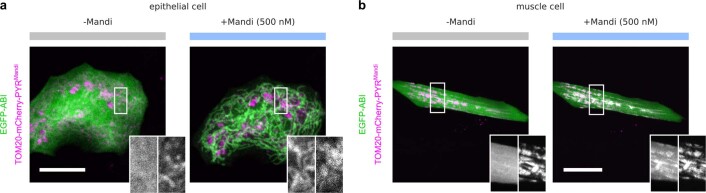
Confocal fluorescence microscopy images of protein colocalization on different cell types in living zebrafish embryos. Cytosolic **r**eceiver and mitochondria localized receptor domains expressed in zebrafish embryos. **a**, epithelial cell **b**, muscle cell. Images acquired before and 10–20 min after addition of Mandi. Scale bar 40 µm. Data representative for 3 independent experiments.

To test this, we expressed receiver and receptor domains on various cellular targets in zebrafish, *Danio rerio*, embryos and evaluated Mandi’s ability to induce protein proximity in tissue 3–5 d postfertilization (dpf; Fig. 3a). At concentrations as low as 500 nM, Mandi successfully induced protein colocalization within minutes at different subcellular targets, that is, in the plasma membrane or mitochondria and in different tissues (Fig. 3b–d, Extended Data Fig. 5 and Supplementary Video 3). Remarkably, addition of Mandi solution on top of the agarose-embedded embryos was sufficient to achieve colocalization in cells deep in the tissue (for example, muscle cells) within minutes, reflecting its excellent tissue penetration. As expected, based on risk assessments related to its use in agriculture^28^, signs of toxicity were not apparent under conditions in which zebrafish embryos were provided with 5 µM of Mandi for 72 h (Supplementary Fig. 7).

### Nanobody-assisted targeting of endogenous proteins using CIP

**Extended Data Fig. 6:**
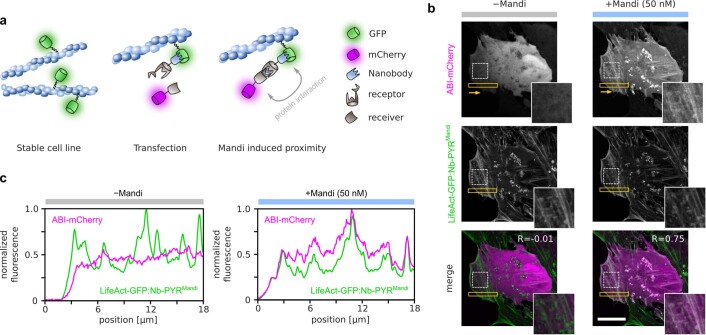
Nanobody assisted targeting of chemically induced protein proximity. **a**, Schematic illustration (a) of nanobody assisted targeting of chemically induced protein proximity. **b**, HeLa cells stably expressing LifeAct-GFP were transfected with antiGFP-nanobody-PYR^Mandi^ and mCherry-ABI fusions. Confocal images acquired before and 5 min after addition of Mandi. Pearson’s correlation coefficients indicated in images. Representative data for 30 cells in 2 independent experiments. Scale bar: 20 µm. **c**, Line profiles of GFP and mCherry signal intensity in ROI (yellow box) before and after addition of Mandi. Source data

Manipulation of endogenous proteins to enable protein interaction studies at native concentrations and in their physiological environment is highly desirable. However, tagging of endogenous proteins can result in altered expression patterns and ill-defined perturbations of protein function. Small drug-like probes for specific protein manipulation have been shown to be highly useful in cell biology research^29^; a generalization for use with arbitrary native proteins is, however, highly challenging. Recent advances using nanobodies have shown great potential for endogenous protein targeting in living cells^30–33^. We hypothesized that nanobody-assisted targeting in combination with the Mandi CIP system could induce artificial interactions between endogenous proteins and any genetically introduced effector protein in a dynamic and controlled manner. As a proof of principle in living cells, we used a well-studied anti-GFP nanobody^34^ in combination with cell lines stably expressing the F-actin-binding protein Lifeact–GFP or paxillin–yellow fluorescent protein (YFP). The anti-GFP nanobody and mCherry were expressed as fusion proteins with the Mandi receptor and receiver, respectively (Extended Data Fig. 6a). The nanobody thus serves as an adaptor between the native target and the artificial CIP system, placing the interaction of effector and endogenous target protein under strict control of Mandi. This was visualized by the appearance of characteristic structures following the addition of Mandi (Extended Data Fig. 6b,c and Supplementary Fig. 8). Provided that the nanobody does not interfere with the function of the endogenous protein of interest, this nanobody-assisted targeting of chemically induced protein proximity can be easily extended to other targets^35^.

### Reversible multi-input CIP system with Mandi

**Extended Data Fig. 7:**
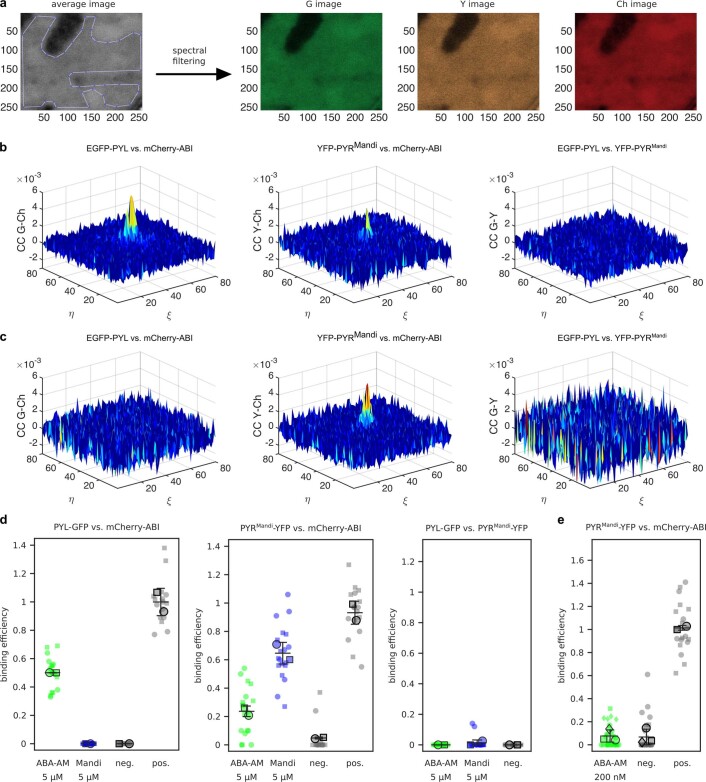
RSICS measurements of Mandi CIP system. **a**, Schematic of RSICS in the ARICS framework. A 256×256 pixel image stack of 300–400 frames acquired in 23 spectral channels is decomposed into three three-dimensional image stacks for GFP (G), YFP (Y) and mCherry (Ch), using a spectral filtering algorithm. An arbitrary ROI delimiting a homogeneous region in the cytoplasm is selected and RSICS analysis is applied to each frame of each image stack. **b,c** CCFs in the three cross-correlation channels obtained from three-color RSICS measurement on COS-7 cells co-expressing EGFP-PYL, YFP-PYR^Mandi^ and mCherry-ABI performed 15 min after incubation with 5 µM ABA-AM **(b)** or 5 µM Mandi **(c)**. **d**, Binding efficiencies for the Mandi/ABA CIP systems in the presence of 5 µM ABA-AM or 5 µM Mandi. For comparison, the binding efficiencies obtained in the negative (neg.) and positive (pos.) cross-correlation control samples are shown. Small symbols: individual data points corresponding to RSICS measurement in a single cell. Large symbols: means from experiments. Mean±SD across experiments indicated by horizontal lines. Data pooled from two independent experiments with 13 (neg.), 18 (ABA-AM), 19 (Mandi) and 16 (pos.) cells. **e**, Binding efficiency of PYR^Mandi^ vs. mCherry-ABI in the presence of 200 nM ABA-AM measured by two-color RSICS. For comparison, the binding efficiencies obtained in the negative (neg.) and positive (pos.) cross-correlation control samples measured under identical conditions are shown. Mean±SD across experiments indicated by horizontal lines. Data pooled from 2 (neg, pos) or 3 (ABA-AM) independent experiments with 28 (AB-AM), 10 (neg) and 20 (pos) cells.

The simultaneous use of multiple CIP systems allows for the construction of Boolean logic gates and enables the design of artificial genetic circuits^13,36^. For such applications, the CIP systems must be orthogonal to the organism under study and among themselves. We tested if the Mandi system could be used in conjunction with GA_3_- and ABA-based CIP systems to create complex logic gates in cell culture systems. As expected, we found Mandi to be fully orthogonal to GA_3_ (Supplementary Fig. 9). ABA and Mandi, however, recruit the identical receiver domain ABI (Supplementary Fig. 10) and, therefore, can be used to create synthetic multi-input systems^37^, which are powerful tools for advanced applications previously only achievable using double fusion constructs^38^. To investigate potential cross-reactivity, we used three-color raster spectral image correlation spectroscopy (RSICS)^39^ to determine the interacting fractions at high CIP concentrations. While Mandi did not show any cross-reactivity with the PYL receptor domain, ABA(-AM) addition resulted in weak interactions between PYR^Mandi^ and ABI (Extended Data Fig. 7). Reducing the ABA-AM concentration to 200 nM minimized the undesired cross-reactivity to a negligible proportion (<10%).

**Fig. 4:**
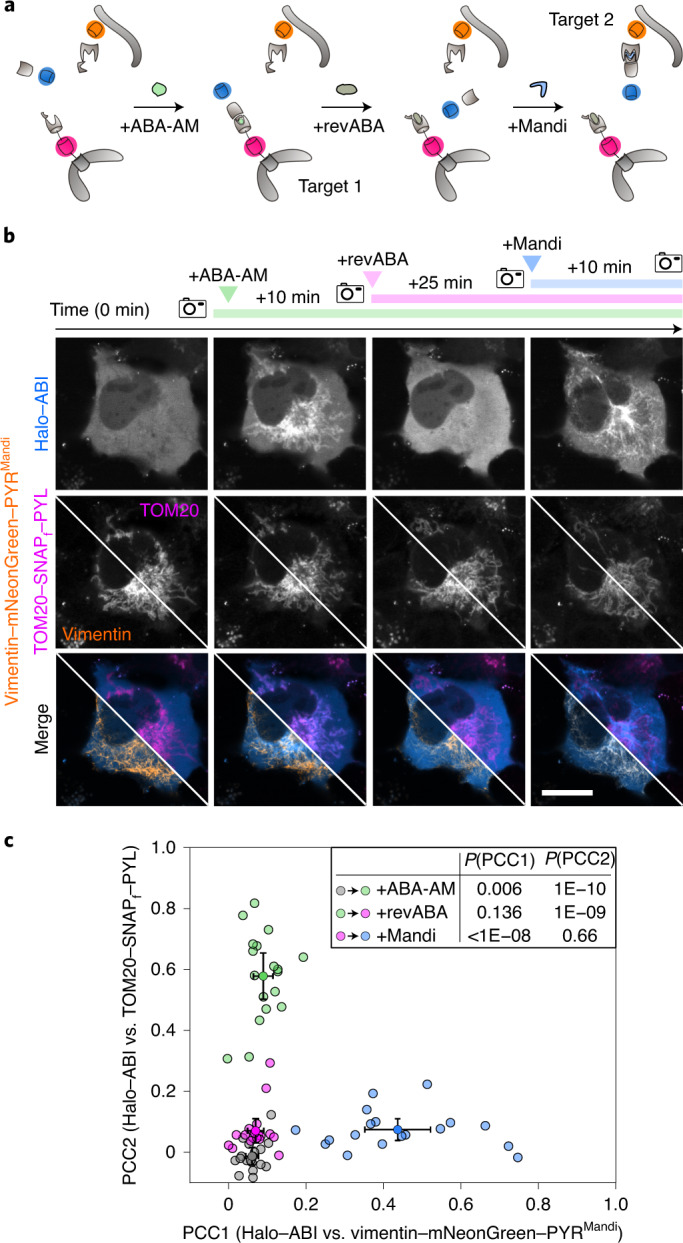
Reversible and dynamic protein shuttling in living cells. **a**, Schematic illustration of the four-step procedure to shuttle cytosolic protein between different intracellular targets. **b**, Confocal fluorescence microscopy images of the shuttling process between vimentin and mitochondria in a living cell. COS-7 cells were cotransfected with vimentin-mNeonGreen-PYR^Mandi^-IRES-Halo-ABI and TOM20-SNAP_f_-PYL. Halo–ABI and SNAP_f_–PYL were labeled with HTL-SiR and tetramethylrhodamine (TMR)-Star, respectively. The top row shows dynamic receiver localization, and the middle row shows receptor localizations as references. Split images depict vimentin and mitochondrial localization in two different channels. The bottom row shows respective merges. Images were acquired at the indicated times before and after the addition of ABA-AM (200 nM), after the addition of revABA (20 μM) and after the addition of Mandi (200 nM); scale bar 20 µm. Data are representative of 22 cells from two independent experiments. **c**, Pearson correlation coefficients (PCC; mean ± s.d.) between receiver and respective receptor channel images at the indicated time points for four-step shuttling between cytosol, mitochondria and vimentin as shown in **b**. Small symbols represent individual cells at the indicated time points. In the inset, conditions were compared with a two-sided paired *t*-test. Source data

**Extended Data Fig. 8:**
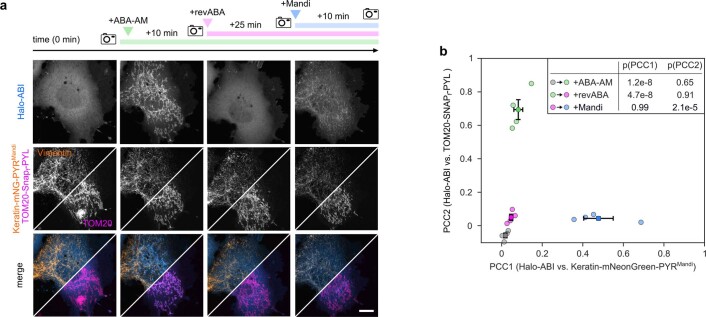
Reversible and dynamic protein shuttling between mitochondria and keratin in living cells. COS-7 cells were co-transfected with Keratin-mNeonGreen-PYR^Mandi^-IRES-Halo-ABI and TOM20-SNAP_f_-PYL. Halo- and SNAP-tag domains were stained with HTL-SiR and TMR-Star 2 h prior to imaging. **a**, Representative confocal fluorescence microscopy images of four-step shuttling between cytosol, mitochondria and keratin over time. Upper row shows dynamic receiver localization, middle row receptor localizations as references, lower row respective merges. Images acquired at t_0_, 10 min after addition of ABA-AM (200 nM), 25 min after addition of revABA (20 μM), 10 min after addition of Mandi (200 nM). Scale bar 20 µm. **b**, Pearson correlation coefficients (PCC) between images receiver and respective receptor channel images at indicated time points for four-step shuttling between cytosol, mitochondria and keratin as shown in (a). 4 cells from 1 experiment. Mean±SD across all cells indicated by dark circles and error bars. Source data

A major challenge in synthetic biology is to mimic complex and highly dynamic intracellular protein networks and to further manipulate their regulation through external stimuli. We designed a multi-input protein translocation system based on different CIP systems where a cytosolic receiver protein is reversibly shuttled between different intracellular targets depending on the specific CIP input (Fig. 4a). Such applications are limited by competing interactions between multiple receptors and the receiver^37^. We addressed this problem by using the synthetic antagonist PANMe (7; Supplementary Fig. 11a)^40^, which we will call revABA hereafter, as a suppressor to selectively inhibit the interaction between ABI and PYL (Supplementary Fig. 11b,c and Supplementary Video 4). Consecutive addition of ABA-AM, revABA and Mandi allowed for controlled shuttling of the cytosolic receiver between mitochondria and vimentin filaments with high efficiency (Fig. 4b). Similar performance was also observed when changing the subcellular localization of receiver domains to the plasma membrane and mitochondria in HEK cells (Supplementary Fig. 12) or mitochondria and keratin filaments in COS-7 cells (Extended Data Fig. 8). We quantified the efficiency of recruitment of the receiver domain to the respective receptor domains by computing Pearson correlation coefficients between individual image pairs before or after small-molecule addition for shuttling between mitochondria and vimentin in COS-7 cells. This analysis confirmed that recruitment with each CIP and suppression of the ABI–PYL interaction with revABA was highly selective, and cross-activation of ABI–PYR^Mandi^ interactions were weak compared to specific induction of ABI–PYL interactions (Fig. 4c).

## Discussion

In summary, we demonstrated that the Mandi CIP system is a versatile technology to control the localization and interaction of proteins and represents an attractive addition to the currently existing set of CIPs. The high cell permeability of Mandi enables immediate protein manipulation in living cells and organisms. We established the use of RICS to evaluate induced protein-binding efficiency in living cells and demonstrate the efficiency of Mandi in comparison to other CIP systems. In gene expression assays, we show that Mandi, comparably to ABA, possesses a large linear dose–response range and therefore has the potential to tune cellular processes in a concentration-dependent manner. Heterobifunctional CIPs have been successfully applied for that purpose, for example, in live cells to control kinetochore function^41^ and in vivo to control adhesion junctions within cell–cell contacts^25^. However, they can lead to hook effects^26,27^, whereas Mandi does not suffer from loss of activity at higher concentrations.

Mandi has an acute oral lethal dose to 50% of animals tested (LD_50_) in rats of >5,000 mg kg^–1^ and a no-observed adverse effect level (NOAEL) of 41 mg kg^–1^ d^–1^ with no evidence of neurotoxicity or genotoxicity or carcinogenic potential in long-term studies^28^. Both, low toxicity and excellent cell permeability allow for Mandi to be used in complex organisms, as demonstrated by the rapid and efficient protein translocation in living zebrafish embryos. In combination with specific nanobodies, we extend the applicability of Mandi to endogenous proteins of interest. By using the Mandi CIP system in conjunction with the ABA-AM system and the abscisic acid antagonist revABA, we realized highly controlled protein shuttling as a basis for advanced manipulation of protein interaction networks.

With respect to these findings, we expect Mandi-based technology to become a versatile and widely used tool for manipulating protein localization and interaction in cell biological research as well as for circuit design in synthetic biology. In consideration of the recent progress of humanized gene therapies and proximity-induced protein degradation, we speculate that the herein reported Mandi CIP system has the potential to be translated into therapeutic applications^42,43^.

## Methods

### Plasmids

For the construction of plasmids, fragments were amplified by PCR from appropriate sources (see Supplementary Information). Primers used for PCR (Supplementary Table 3) were delivered by Integrated DNA Technologies (IDT). The PCR reaction mix of backbone fragments was digested with DPN1 (addition of 10 µl of CutSmart Buffer + 1 µl of DPN1 to 50 µl of PCR mix and incubation at 37 °C for 1 h). All PCR fragments were purified by preparative agarose gel electrophoresis and extracted with a QIAquick gel extraction kit (Qiagen). Ligation by Gibson assembly was performed in equimolar ratios of all fragments. Plasmid sequences were validated by Sanger sequencing (Seqlab) using either standard primers or premixed sequence primers. See Supplementary Information for fragment sources and specific procedures for all plasmids used in this study (Supplementary Fig. 13). All plasmids will be made available via Addgene after final publication.

### Cell culture, transfection and sample preparation

Cells were grown at 37 °C and 5.0% CO_2_ in Dulbecco’s Modified Eagle Medium (DMEM; Sigma-Aldrich) supplemented with 2 mM l-glutamine, 1 mM sodium pyruvate and 10% (vol/vol) fetal bovine serum. Cells were routinely passaged after 2–3 d or upon reaching 80% confluency. Before seeding cells, type number 1 eight-well LabTek chambered coverslips (Thermo Fisher Scientific) were cleaned with 0.1 M hydrofluoric acid to improve cell attachment. Twenty-four hours before imaging, cells seeded in LabTek chambers were transiently transfected using FuGene HD (Promega) or TransIT-X2 (Mirus) transfection reagents according to manufacturer’s guidelines. Cells transfected with HaloTag or SNAP_f_-tag fusion constructs were labeled before imaging. Growth medium was exchanged with staining solution containing either 20 nM HTL-SiR or 200 nM TMR-Star (New England Biolabs) in DMEM and incubated between 20 min (HaloTag labeling) and up to 1 h (SNAP_f_-tag labeling).

For mitochondria staining, cells were incubated with 200 nM MitoTracker Orange CMTMRos (Thermo Fisher Scientific) in DMEM for 1 h according to manufacturer’s guidelines. All measurements were performed in Leibovitz L15 medium (Sigma-Aldrich). All used CIPs were purified by preparative HPLC. Lyophilized products were dissolved in DMSO, and stocks were diluted in L15 medium. Final DMSO concentrations were kept below 2% for all experiments.

### Protein shuttling and colocalization analysis

Samples with HEK293T (DSMZ) or COS-7 cells (ATCC) were prepared according to general remarks. Imaging was initiated in 150 µl of L15 medium. CIPs were subsequently added as 2×, 3× or 4× stocks in 150 µl of L15 medium, respectively. The final DMSO concentration was kept below 2%. Images were acquired after the incubation times indicated in the respective figures.

For quantitative analysis of shuttling efficiency, confocal *z* stacks were acquired at each time point, and individual cells in each stack were segmented manually. Pearson correlation coefficients between receiver channel and both receptor channels were then computed using custom-written ImageJ scripts. Each image was background corrected by subtracting a copy of the respective image that was smoothed by convolution with a 20-pixel Gaussian. After background correction, Pearson *R* values were calculated using the ImageJ plugin Coloc2 for each *z* slice separately. The final Pearson *R* value for each cell was obtained by averaging *R* values from individual *z* slices.

### Nanobody-assisted targeting of chemically induced protein proximity

The stable cell lines HeLa (Lifeact–GFP–Halo; a gift from J. Piehler, University of Osnabrück, Germany) and REF (paxillin–YFP; a gift from A. Cavalcanti-Adam, Max-Planck Institute for Medical Research, Heidelberg, Germany) were transfected with pnanobody-PYR^Mandi^ and pmCherry-ABI constructs 24 h before imaging. Growth medium was exchanged, and imaging was performed in 200 µl of L15 medium. Mandi was added at a 2× final concentration in 200 µl of L15 medium to a final concentration of 50 nM.

### Wide-field epifluorescence microscopy

Wide-field imaging was performed using an inverted epifluorescence microscope equipped with an Apo TIRF ×100/1.49-NA oil immersion objective (Nikon). An iChrome MLE-LFA multilaser engine (Toptica Photonics) containing four lasers emitting at 405, 488, 561 and 640 nm was used as the light source and fiber coupled into the microscope using a TIRF illumination module (Nikon). Focus stabilization in time-lapse imaging was achieved using a perfect focus system (PFS3, Nikon). Excitation and emission light were separated using a quad-edge dichroic beamsplitter, and emitted light was further filtered using bandpass filters (AHF Analysetechnik). Images were acquired using an iXon+ 897 Ultra electron-multiplying CCD camera (Oxford Instruments Andor), which was also used as a timing device to synchronize excitation lasers and camera exposures during imaging with alternating laser excitation. The microscope and all connected devices were controlled using the Micromanager software platform^44^. Typically, images were acquired with a 50-ms exposure at 5–10 W cm^–2^ illumination intensity and at a 95- or 146-nm pixel size. Multispectral images were acquired using a motorized filter wheel equipped with 525/50-nm (eGFP), 605/70-nm (mCherry, TMR) and 685/70-nm (SiR) bandpass filters.

### Confocal fluorescence microscopy

Imaging was performed on a commercially available confocal microscope (A1R, Nikon). The microscope is built around an inverted, motorized Ti2-E stand and is equipped with a galvanometric scanner, a perfect focus system (Nikon) and a stage-top incubation chamber for temperature control and CO_2_ injection (Tokai Hit). An Apo λs ×60/1.4-NA oil immersion objective was used for excitation and collection of emitted fluorescence. Solid-state lasers (488, 561 and 638 nm) (Nikon) were used for excitation, and a 405/488/561/640-nm quad band dichroic was used for separating excitation from emission light paths. Typically, 34 µW of 488-nm light, 11.5 µW of 561-nm light and 195 µW of 638-nm light were used for excitation. Signal from eGFP, mCherry/TMR and SiR was further filtered using 515/30-nm, 595/50-nm and 700/75-nm bandpass filters, respectively. For 488-nm and 561-nm detection channels, GaAsP detectors were used for detection. Detection of signal following 638-nm excitation was performed using a photomultiplier tube as a detector. A pixel size of 110 nm and a scan speed of 2.4 µs per pixel with 2× line averaging were applied for all data acquisitions. The pinhole was set to a size of 1.2 Airy units. *Z* stacks were recorded with a spacing of 500 nm. Nikon Elements was used to control image acquisition and all connected devices.

### Screening of CIP efficiency

#### Sample preparation

COS-7 cells were seeded into eight-well LabTek chambered coverslips. Transient transfection with plasmids expressing both receiver and receptor domains as cytosolic GFP and TOM20–mCherry fusions linked with an IRES sequence was conducted as described above (Supplementary Table 3). The mCherry-tagged mitochondrial receiver or receptor served as signal for segmentation of mitochondria and to determine the area to which cytosolic eGFP-tagged protein was recruited (see below). Twenty-four hours after transfection, cells were washed once with L15 medium and then imaged in L15 at room temperature. CIP solution was freshly prepared from DMSO stocks at 2× final concentration in L15 before imaging.

#### Acquisition

Automated time-lapse epifluorescence imaging was performed on a Nikon epifluorescence setup described above. Multispectral images were acquired with alternating laser excitation between frames, where excitation lasers were controlled by an Arduino microcontroller synchronized to the emCCD camera. Typically, images were acquired with 50-ms exposure per image and variable lag times between individual image pairs depending on the typical times to effect for the individual CIP systems (Supplementary Table 2). The end point of the time-lapse was chosen so that no further recruitment of the cytosolic signal to mitochondria was observed. The total number of images per post-CIP addition time-lapse was kept constant to minimize differences due to photobleaching or phototoxicity between CIPs. eGFP and mCherry were excited with CW laser illumination at 0.35- and 0.51-mW output at the objective corresponding to an average irradiance of 5.3 and 8.6 W cm^–2^ across the readout region. Emitted fluorescence was split using an Optosplit II image splitter (Cairn Research) equipped with a 560-nm longpass beamsplitter (AHF Analysetechnik) and additionally filtered using 605/670-nm (mCherry) and 525/550-nm (eGFP) bandpass filters inserted in the reflected and transmitted light paths, respectively. Signals from both paths were recombined using a second 560-nm shortpass filter and two two-axis translation mirrors. Manual coarse alignment of both channels was achieved using 0.1-µm Tetraspek multifluorescent beads (Thermo Fisher Scientific) as reference. For each experiment, a single cell in each well was manually selected with receiver and receptor expression and general cell morphology as selection criteria. Automated data acquisition was then performed using a custom-written µManager^44^ beanshell script. In brief, each acquisition consisted of a 488/561-nm excitation image pair before CIP addition (*t*_0_), CIP addition, time-lapse acquisition and acquisition of a final *t*_end_ image pair (Supplementary Fig. 2). Injection of CIP was performed with a computer-controlled Aladdin AL1000 microfluidic pump (World Precision Instruments) at a flow rate of 6 ml min^–1^. CIP was added at equal volume and double final concentration followed by a 2- to 4-s delay to allow for mixing of medium in the well with the added CIP solution. This procedure was repeated for each well on one slide.

#### Data preprocessing

Acquired multidimensional image stacks were processed in Fiji^45^ using custom-written analysis routines. Raw data were automatically checked for errors in illumination sequences, and corresponding image pairs were removed from time-lapse datasets. Across all acquisitions, <1% of image pairs were discarded during this step. Flat fielding to correct for differences in excitation intensity was performed by multiplying all images with a template image. Illumination profile templates were obtained by acquiring 20–30 images of surfaces homogeneously coated with Alexa Fluor 488 or TMR NHS esters for 488-nm and 561-nm excitation, respectively. Images were then averaged and normalized to the maximum value in the averaged image. To correct for variations in alignment of the microscope, new templates were measured for each round of experiments. Image pairs were spatially aligned with subpixel accuracy using the Image Stabilizer Plugin authored by K. Li (http://www.cs.cmu.edu/~kangli/code/Image_Stabilizer.html). Tetraspek beads (0.1 µm) served as reference structures to compute transformation coefficients. Transformation coefficients were determined separately for each experiment.

#### Segmentation and intensity extraction

Raw image data were automatically segmented using the Trainable Weka Segmentation package^18^. Models for classification of total cell area and mitochondria were trained by manual classification of ten randomly selected images from the entire dataset. The model for cell detection was trained and applied using 488-nm excitation *t*_0_ images, which exhibit purely cytosolic signal. The mitochondria model was trained using 561-nm excitation *t*_0_ images. The obtained segmentations were robust with respect to the average area occupied by mitochondria in any given image set, which typically was between 10 and 40% (Supplementary Fig. 3), and no systematic variation in segmented mitochondrial area in individual time-lapse datasets was observed (Supplementary Fig. 3b).

Regions of interest (ROIs) were obtained from Weka segmentation results by thresholding of segmentation maps. Cytosolic ROIs were obtained by computing the difference between the whole cell and mitochondria for each image pair. Because the cytosolic signal gradually translocated to mitochondria after CIP addition, cytosolic ROIs for time-lapse and *t*_end_ image pairs were computed using the *t*_0_ whole-cell ROI and the mitochondria from the current image pair. As expected, the whole-cell intensity in the 488-nm channel remained unchanged (<2% variation) after CIP addition, indicating that cell movement during time-lapse image acquisition was negligible (Supplementary Fig. 3c,d). After segmentation, average intensities for each ROI and image were extracted and exported as text files. Acquisition timestamps were extracted from image metadata and included in text files.

#### Plotting

Translocation ratios were computed using intensities extracted from *t*_0_, *t*_end_ image pairs and time-lapse data. All calculations and plots were created using MATLAB 2018a (The MathWorks). In the first step, all data were corrected for photobleaching using the decay in whole-frame intensity during acquisition. Then, the ratio of mean 488-nm intensity in the mitochondria and cytosol ROI for each frame of the time-lapse dataset was computed. The obtained ratios were corrected for the ratio before CIP addition (ratio_*t*0_) and the final ratio after time-lapse acquisition (ratio_*t*end_). No ratio was computed for frames with erroneous illumination sequence. Datasets where segmentation was not reliable were identified using the average mitochondrial 561-nm signal over time and excluded from analysis. The fraction of cells excluded was <10% across all CIPs (Supplementary Table 2). All code required for acquisition and processing of raw data will be made available after final revision of the manuscript.

### Luciferase expression assay

#### Sample preparation

For luciferase expression experiments, cells were transfected during cell seeding. For each well, a mixture of 10 µl of OptiMEM and 0.3 µl of Lipofectamine 3000 was added to 10 µl of OptiMEM, 0.4 µl of P3000 and 200 ng of DNA (equally split between pGL 4.31 (Promega) and pVP16AD-PYR^Mandi^-IRES-GAL4BD-ABI/SV-ABAactDA (Addgene, 38247)) and incubated for 25 min. The transfection mix was added to suspended cells (8.4 × 10^5^ cells per ml), and 3 × 10^3^ cells were seeded in each well of a 96-well plate (TPP). After 17 h, cells were washed with 100 µl of PBS and exchanged to 100 µl of DMEM. Twenty-four hours after transfection, cells were treated with 100 µl of the respective CIP concentration in DMEM and incubated for an additional 24 h. Cells were washed with 100 µl of PBS and lysed with 50 µl of 1× Passive Lysis Buffer (Promega) at room temperature for 10 min on a shaker (80 r.p.m.). Cell lysate (20 µl) was used for the luciferase assay.

#### Data acquisition

Luciferase Assay Reagent (100 µl; Promega) was added to 20 µl of the cell lysate by a plate reader-mounted injector. After addition, signal was recorded with a 3-s delay (shaking) and 2-s integration for 40 s with a Tecan Spark plate reader at 22 °C.

#### Data analysis

Integrated signals from individual wells were computed as the sum of 20 individual readings per well. Signals from wells containing CIPs were normalized to control wells exposed to DMSO. Median luciferase activities at different CIP concentrations were computed across all replicates from all experiments for a given condition. EC_50_ values were obtained by fitting median luciferase response profiles with a Hill equation using MATLAB 2020a.

$$R = \frac{{R_{\max }}}{{1 + \left( {\frac{{{\textrm{EC}}_{50}}}{{\left[ {{\textrm{CIP}}} \right]}}} \right)}}$$

*R* is the measured luciferase signal, *R*_max_ is the maximum asymptote and [CIP] indicates the CIP concentration.

### Split TEV recombination assay

#### Sample preparation

For the split TEV recombination assay, COS-7 cells were transfected during cell seeding. For each well, a mixture of 10 µl of OptiMEM and 0.3 µl of Lipofectamine 3000 was added to 10 µl of OptiMEM, 0.4 µl of P3000 and 200 ng of DNA (equally split between cLuc (Addgene, 119207) and pnTEV-PYR^Mandi^ and pcTEV-ABI (Addgene, 119214)) and incubated for 25 min. The transfection mix was added to suspended cells at a density of 8.4 × 10^4^ cells per ml, and 3 × 10^3^ cells were seeded in each well of a 96-well plate (TPP). After 48 h, cells were washed with 100 µl of PBS, and 100 µl of CIP solution in DMEM was added and incubated for 1 h. Cells were washed with 100 µl of PBS and lysed with 50 µl of 1× Passive Lysis Buffer (Promega) at room temperature for 10 min on a shaker (80 r.p.m.). Cell lysate (20 µl) was used for the luciferase assay.

#### Data acquisition

Luciferase Assay Reagent (100 µl; Promega) was added to 20 µl of the cell lysate by a plate reader-mounted injector. After addition, signal was recorded with a 3-s delay (shaking) and 2-s integration for 40 s with a Tecan Spark plate reader at 22 °C.

#### Data analysis

The integrated signals of each well were calculated as the sum of 20 individual measurements per well. Signal from wells containing CIPs were normalized to control wells exposed to DMSO. Conditions were compared using unpaired *t*-tests with Welch’s correction.

### Raster spectral image correlation spectroscopy

#### Sample preparation

For RSICS experiments, 10^5^ COS-7 cells were seeded in 35-mm number 1.5 optical glass-bottom dishes (CellVis) 24 h before transfection. Cells were cotransfected with 10 ng of pYFP-PYR^Mandi^, 200 ng of peGFP-PYR, 250 ng of pmCherry-ABI, 50 ng of peGFP-FRB or 50 ng of pmCherry-FKBP12 and imaged 20 h after transfection. For the negative cross-correlation control, cells were cotransfected with 50 ng of peGFP-N1, YFP-N1 and pmCherry-N1 vectors. To calibrate the maximum cross-correlation of the setup, positive-control samples were prepared by transfecting cells with 50 ng of pmCherry-eGFP or pmCherry-YFP heterodimer constructs, as described previously^46^. For single-species samples, cells were transfected with 50 ng of peGFP-N1, pYFP-N1 or pmCherry-N1. All transfections were performed using Lipofectamine 3000 according to the manufacturer’s instructions (Thermo Fisher Scientific). Further information on plasmids for control measurements can be found in Dunsing et al.^46^. RSICS measurements with CIPs were performed after 15-min incubation of samples.

#### Data acquisition

RSICS measurements were performed on a Zeiss LSM 880 system (Carl Zeiss) using a Plan Apochromat ×40/1.2 Korr DIC M27 water immersion objective. Per measurement, 300–400 frames of 256 × 256 pixels were acquired with a 50-nm pixel size (that is, a scan area of 12.83 × 12.83 µm through the midplane of cells), a 2.05-µs pixel dwell time and a 1.23-ms line and 314.57-ms frame time (corresponding to ca. 1.5–2 min of total acquisition time). Samples were excited with a 488-nm argon laser and a 561-nm diode laser at ca. 4.8-µW (488 nm) and 5.9-µW (561 nm) excitation powers, respectively. Laser powers were chosen to maximize the signal emitted by each fluorophore species and to maintain photobleaching below 25% for all species. Typical counts per molecule were ca. 25 kHz for eGFP, 15–20 kHz for YFP and 10 kHz for mCherry. To split excitation and emission light, a 488/561-nm dichroic mirror was used. Fluorescence was detected between 490 nm and 695 nm in 23 spectral channels of 8.9 nm on a 32-channel GaAsP array detector operating in photon counting mode. To obtain reference emission spectra for each individual fluorophore species, four image stacks of 25 frames were acquired at the same imaging settings on single-species samples on each day. In addition, negative and positive cross-correlation control samples were measured on each day. All measurements were performed at room temperature.

#### Data analysis

RSICS analysis followed the implementation described recently^39,47^, which is based on applying the mathematical framework of fluorescence lifetime and fluorescence spectral correlation spectroscopy^48,49^ to RICS. Four-dimensional image stacks were imported in MATLAB (The MathWorks) from CZI image files using the Bioformats package^49^ and further analyzed using custom-written code. First, average reference emission spectra were calculated for each individual fluorophore species from single-species measurements. Four-dimensional image stacks were then decomposed into two (eGFP, mCherry/YFP, mCherry) or three (eGFP, YFP, mCherry) three-dimensional image stacks (eGFP, YFP, mCherry) using the spectral-filtering algorithm presented by Schrimpf et al.^39^. Cross-correlation RICS analysis was performed in the arbitrary region RICS (ARICS) framework^50^. To this aim, an ROI was selected in the time- and channel-averaged image frame containing a homogeneous region in the cytoplasm of cells. This approach allowed for the exclusion of visible intracellular organelles or pixels in the extracellular space. Image stacks were further processed with a high-pass filter (with a moving four-frame window) to remove slow signal variations and spatial inhomogeneties. Afterwards, RICS autocorrelation functions (ACFs) and pair-wise CCFs were calculated for each image stack and the eGFP–mCherry/YFP–mCherry or all detection channel combinations (eGFP–YFP, eGFP–mCherry, YFP–mCherry) for two- or three-color experiments, respectively^39,50^. A normal diffusion RICS fit model^21,51^ was then fitted to ACFs and CCFs. From the amplitudes of the ACFs and CCFs, the relative cross-correlation (rel.cc.) was calculated for each three cross-correlation (CC) combination (eGFP–YFP, eGFP–mCherry, YFP–mCherry)

$${\textrm{rel.cc}} = {\textrm{max}}\left\{ {\frac{{G_{{\textrm{CC}},ij}(0,0)}}{{G_{{\textrm{AC}},i}(0,0)}},\frac{{G_{{\textrm{CC}},ij}(0,0)}}{{G_{{\textrm{AC}},j}(0,0)}}} \right\}$$

where *G*_CC*,ij*_(0,0) is the amplitude of the CCF of species *i* and *j*, and *G*_AC*,i*_(0,0) is the amplitude of the ACF of species *i*. The convergence of the RICS fit to the CCFs allowed for the discrimination between weak and no binding (that is, in the absence of interactions, the CCFs are dominated by noise, and the RICS fit does not converge to meaningful parameters, as previously described^47^). In the latter case, the rel.cc. was set to zero. Binding efficiencies were calculated by subtracting the residual average rel.cc. measured in each cross-correlation channel of the negative control (containing two or three mixed fluorescent protein (FP) species) from the measured cross-correlation. The result was then normalized using the average rel.cc. obtained from the positive cross-correlation controls (containing eGFP–mCherry or eGFP–YFP heterodimers). The positive controls account for imperfect alignment of the optical observation volumes and non-fluorescent states of the fluorescent protein tags (for example, due to limited maturation or dark states^46,52^). To ensure statistical robustness of the three-color RICS analysis and sufficient signal-to-noise ratios, the analysis was restricted to cells expressing all three fluorophore species in comparable amounts, that is, relative average signal intensities of less than three for all species.

### Zebrafish strains

The AB_2_O_2_ wild-type line (European Zebrafish Resource Centre EZRC, Karlsruhe) was used for all experiments. Zebrafish husbandry^53^ and experimental procedures were performed in accordance with German animal protection regulations (Regierungspräsidium Karlsruhe, Germany, 35-9185.64/BH KIT).

### Real-time imaging of zebrafish embryos

peGFP-ABI and pTOM20-mCherry-PYR^Mandi^ or pLYN-mCherry-PYR^Mandi^ plasmids were injected into the yolk of one- to two-cell embryos^54^. Positive coexpressing 3- to 5-d-old embryos, which were immobilized on a microscopy slide using 0.5% low melting point agarose supplemented with 0.02% MESAB, were used. Embryos were imaged with a ×63/0.9-NA HCX Apo water-dipping objective installed on a Leica TCS SP2 confocal microscope and the corresponding Leica LCS software (Leica). All experiments were performed at room temperature. Mandi in water (50 µM stock solution in DMSO; 500 nM final concentration) was added on top of the embedded embryos.

### Reporting Summary

Further information on research design is available in the Nature Research Reporting Summary linked to this article.

## Online content

Any methods, additional references, Nature Research reporting summaries, source data, extended data, supplementary information, acknowledgements, peer review information; details of author contributions and competing interests; and statements of data and code availability are available at 10.1038/s41589-021-00922-3.

## Supplementary information

Supplementary Information Supplementary Tables 1–3, Figs. 1–13, References and Notes 1 and 2.

Reporting Summary

Supplementary Video 1 Translocation of cytosolic eGFP–ABI to mitochondria-localized TOM20–mCherry–PYR^Mandi^ after addition of Mandi to a 100 nM final concentration. COS-7 cells were transiently transfected with TOM20-mCherry-PYR^Mandi^-IRES-eGFP-ABI. Epifluorescence time-lapse images were acquired after addition of Mandi at *t* = 0 s. Raw data were background subtracted, flatfielded to correct for Gaussian-shaped illumination profiles and temporally smoothed with a two-frame running average; scale bar, 10 µm. Data are representative of six cells.

Supplementary Video 2 Translocation of cytosolic eGFP–ABI to mitochondria-localized TOM20–mCherry–PYR^Mandi^ after addition of Mandi to a 10 nM final concentration. COS-7 cells were transiently transfected with TOM20-mCherry-PYR^Mandi^-IRES-eGFP-ABI. Epifluorescence time-lapse images were acquired after addition of Mandi at *t* = 0 s. Image pairs with 488-nm and 561-nm illumination for eGFP (green) and mCherry (magenta) excitation, respectively, were acquired for each time point. Raw data were background subtracted, flatfielded to correct for Gaussian-shaped illumination profiles and temporally smoothed by a two-frame running average projection; scale bar, 10 µm. Data are representative of 12 cells from two independent experiments.

Supplementary Video 3 Translocation of cytosolic eGFP–ABI to plasma membrane-localized LYN–mCherry–PYR^Mandi^ in vivo. eGFP-ABI and LYN-mCherry-PYR^Mandi^ plasmids were injected in the yolk of one- to two-cell zebrafish embryos. Recruitment of eGFP to the plasma membrane after addition of Mandi (final concentration of 500 nM) at *t* = 0 min, followed by confocal microscopy. Raw data were corrected for photobleaching and temporally smoothed by a two-frame running average projection; scale bar, 40 µm. Data are representative of three independent experiments.

Supplementary Video 4 Recruitment of ABI to PYL can be efficiently reversed using revABA. COS-7 cells were transiently transfected with TOM20-mCherry-PYL-IRES-eGFP-ABI. eGFP–ABI was recruited to mitochondria by incubation with 200 nM ABA for 2 h. Directly before imaging, medium was replaced with L15 containing 200 nM ABA. revABA at a concentration of 10 µM was then added during imaging at time point *t* = 0 min, and eGFP–ABI localization was followed over time; scale bar, 10 µm. Data are representative of 20 cells from three independent experiments.

## Data Availability

The datasets generated during this study are available from the corresponding author upon request. Plasmids will be deposited at Addgene. Source data are provided with this paper.

## mandipropamid

PubChemID: 445470557
InChIKey: KWLVWJPJKJMCSH-UHFFFAOYSA-N
MDL Molfile
Chemdraw file

## gibberellic acid acetoxymethyl ester

PubChemID: 445470551
InChIKey: IVIYFJSPDLOPEX-AXKFECAUSA-N
MDL Molfile
Chemdraw file

## (+)-abscisic acid

PubChemID: 445470552
InChIKey: JLIDBLDQVAYHNE-YKALOCIXSA-N
MDL Molfile
Chemdraw file

## (+)-abscisic acid acetoxymethyl ester

PubChemID: 445470553
InChIKey: DCBYVLVTJLMSHG-OROXUCHXSA-N
MDL Molfile
Chemdraw file

## rapamycin

PubChemID: 445470554
InChIKey: QFJCIRLUMZQUOT-HPLJOQBZSA-N
MDL Molfile
Chemdraw file

## gibberellic acid

PubChemID: 445470555
InChIKey: IXORZMNAPKEEDV-OBDJNFEBSA-N
MDL Molfile
Chemdraw file

## revABA

PubChemID: 445470556
InChIKey: WPPIXXDTHHCJMC-JJTXBFMGSA-N
MDL Molfile
Chemdraw file

## Source data

Source Data Fig. 2 Single-cell trajectories, luminescence intensities of luciferase assay and two-color RICS binding efficiencies.
Source Data Fig. 4 Images for Pearson’s coefficient determination, ROIs and calculated Pearson’s coefficients.
Source Data Extended Data Fig. 4 Unprocessed luminescence intensities of split luciferase assay.
Source Data Extended Data Fig. 6 Images for line profiles, ROIs and intensity line profiles.
Source Data Extended Data Fig. 8 Images for Pearson’s coefficient determination, ROIs and calculated Pearson’s coefficients.

## Code availability

MATLAB code for RSICS analysis is available at GitHub (https://github.com/VaDu8989/SpectralFFS). Custom ImageJ/Fiji scripts for processing time-lapse microscopy data for analysis of CIP kinetics is available at GitHub (https://github.com/KlausYserentant/CIPKinetics).
