# Supplementary material for: Mandipropamid as a chemical inducer of proximity for in vivo applications
Source: Nat Chem Biol. 2021 Dec 21;18(1):64–9. doi: 10.1038/s41589-021-00922-3 (PMC8709788; doi:10.1038/s41589-021-00922-3)

nature portfolio

https://doi.org/10.1038/s41589-021-00922-3

Supplementary information

Mandipropamid as a chemical inducer of proximity for in vivo applications

In the format provided by the authors and unedited

SUPPLEMENTARY INFORMATION

Mandipropamid as Chemical Inducer of Proximity for in vivo Application and Protein Network Manipulation

Michael J. Ziegler[1,2‡], Klaus Yserentant[3-5 ‡], Valentin Dunsing[6], Volker Middel[7], Antoni J. Gralak[1,3], Kaisa Pakari[3], Jörn Bargstedt[1], Christoph Kern[1,3], Annett Petrich[6], Salvatore Chiantia[6], Uwe Strähle[7], Dirk-Peter Herten[3,5,8] and Richard Wombacher[1,2*]

[1] Institute of Pharmacy and Molecular Biotechnology, Heidelberg University, Im Neuenheimer Feld 364, 69120 Heidelberg, Germany.

[2] Max Planck Institute for Medical Research, Department of Chemical Biology, Jahnstrasse 29, 69120 Heidelberg, Germany.

[3] Institute of Physical Chemistry, Heidelberg University, Im Neuenheimer Feld 229, 69120 Heidelberg, Germany.

[4] Faculty of Biosciences, Heidelberg University, Im Neuenheimer Feld 234, 69120 Heidelberg, Germany.

[5] Institute of Cardiovascular Sciences & School of Chemistry, College of Medical and Dental Sciences, University of Birmingham, Birmingham, B15 2TT, United Kingdom.

[6] University of Potsdam, Institute of Biology and Biochemistry, Karl-Liebknecht-Str. 24-25, 14476 Potsdam, Germany.

[7] Institute of Biological and Chemical Systems (IBCS) - Biological Information Processing (BIP), Karlsruhe Institute of Technology (KIT), 76344 Eggenstein-Leopoldshafen, Germany.

[8] Centre of Membrane Proteins and Receptors (COMPARE), Universities of Birmingham and Nottingham, Midlands, United Kingdom.

‡ These authors contributed equally

* Corresponding author: wombacher@mr.mpg.de

Supplementary Information

1 SUPPLEMENTARY TABLES 3

2 SUPPLEMENTARY FIGURES 5

3 SUPPLEMENTARY REFERENCES 16

SUPPLEMENTARY NOTE 1: PLASMID DESIGN AND PREPARATION 17

SUPPLEMENTARY NOTE 2: SYNTHESIS AND CHARACTERIZATION 22

1 Supplementary Tables

Supplementary Table 1: Comparison of cost of purchase for different CIPs.

	Price	Price/100 mg [EUR]	Source
Mandipropamid[1]	133 EUR/100 mg	133	Sigma-Aldrich
Mandipropamid[2]	880 USD/2.84 kg	<<1	Keystone
Rapamycin[1]	279 EUR/10 mg	2,790	Sigma-Aldrich
Rapamycin	71 EUR/100 mg	71	MedChemExpress
Giberellic acid (GA_3)	62 EUR/250 mg	24.8	Sigma-Aldrich
GA_3 AM ester (GA_3-AM)	69 EUR/10 mg	690	Sigma-Aldrich
Abscisic acid (ABA)	128 EUR/100 mg	128	Sigma-Aldrich
ABA AM ester (ABA-AM)[3]	-	-	no commercial source
AP21967	876 EUR/5 mg	17,520	TaKaRa Bio
AP20187[4]	2842 EUR/50 mg	5,684	MedChemExpress
iRap	-	-	no commercial source

[1] Analytical standard
[2] If purified from Revus Top® as described in Materials & Methods section of our manuscript.
[3] Developed in this study
[4] Homodimerizer

Supplementary Table 2: Experimental design for automated measurements of CIP-induced protein translocation. See Supplementary Fig. 21 for plasmid ids.

CIP	CIP con-centration [μM]	Acquisition frame rate [pair/min]	Image pairs per acquisition	Plasmid	Fraction cells successfully processed [%]	# cells (experiments)
GA_3-AM	5	12	120	p#01	96.8	30 (n=4)
ABA	5	8	120	p#02	92.0	23 (n=4)
ABA-AM	5	12	120	p#02	93.8	30 (n=4)
Mandi	5	24	120	p#07	91.7	13 (n=2)
Mandi	0.5	60	300	p#07	100	11 (n=2)
Mandi	0.05	60	300	p#07	100	28 (n=4)
Rapamycin	0.5	60	300	pMito-mCherry-FRB (Addgene #59352), eGFPC1 FKBP^{F36V} (Addgene #67529)*	94	16 (n=2)

* For translocation experiments FKBP12^{F36V} was used, which showed comparable recruitment kinetics as FKBP12 wildtype

2 Supplementary Figures

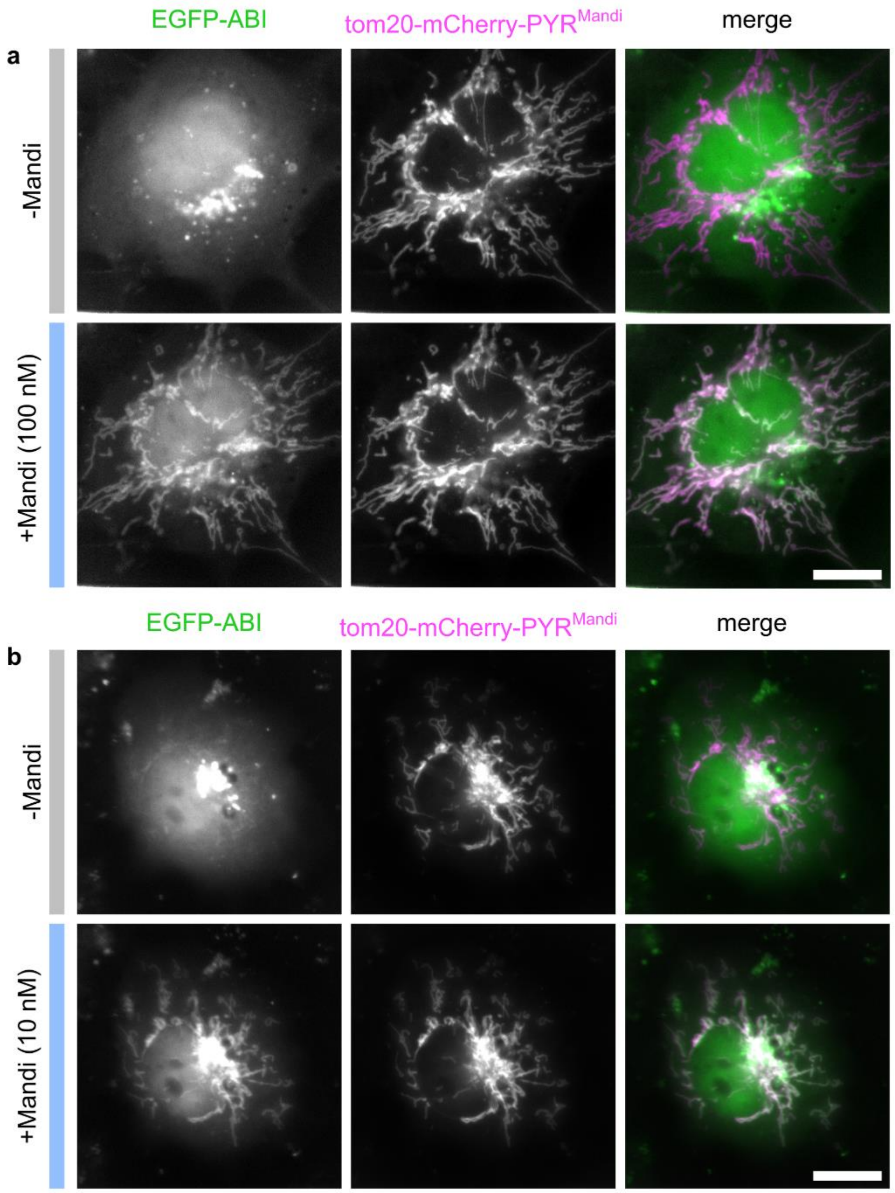

Supplementary Figure 1: Live-cell epifluorescence microscopy images of Mandi-induced translocation at different concentrations. COS-7 cells were transfected with TOM20-mCherry-PYR^{Mandi}-IRES-EGFP-ABI. Images were acquired before addition of 100 nM (a) or 10 nM (b) Mandi and after completion of translocation. Timelapse of translocation process shown in supplementary videos 1,2. Colocalization was completed within 1 min (a) and 3-5 min (b). Scale bar 20 µm. Representative data for 6 (a) or 12 (b) cells from 2 independent experiments.

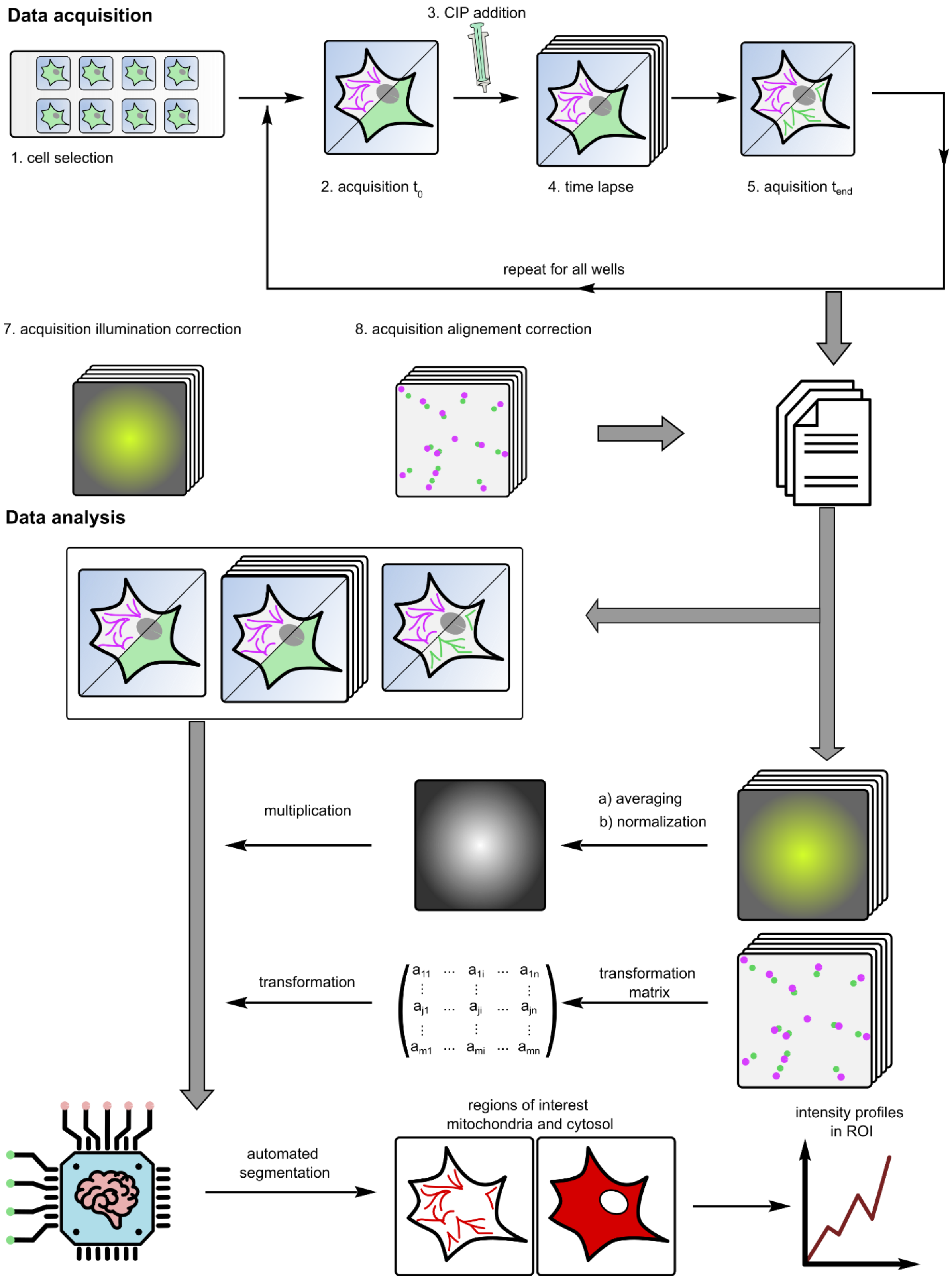

Supplementary Figure 2: Screening of CIP efficiency with automated microscopy. Experimental workflow consisting of manual cell selection, automated microscopy and CIP addition and data processing.

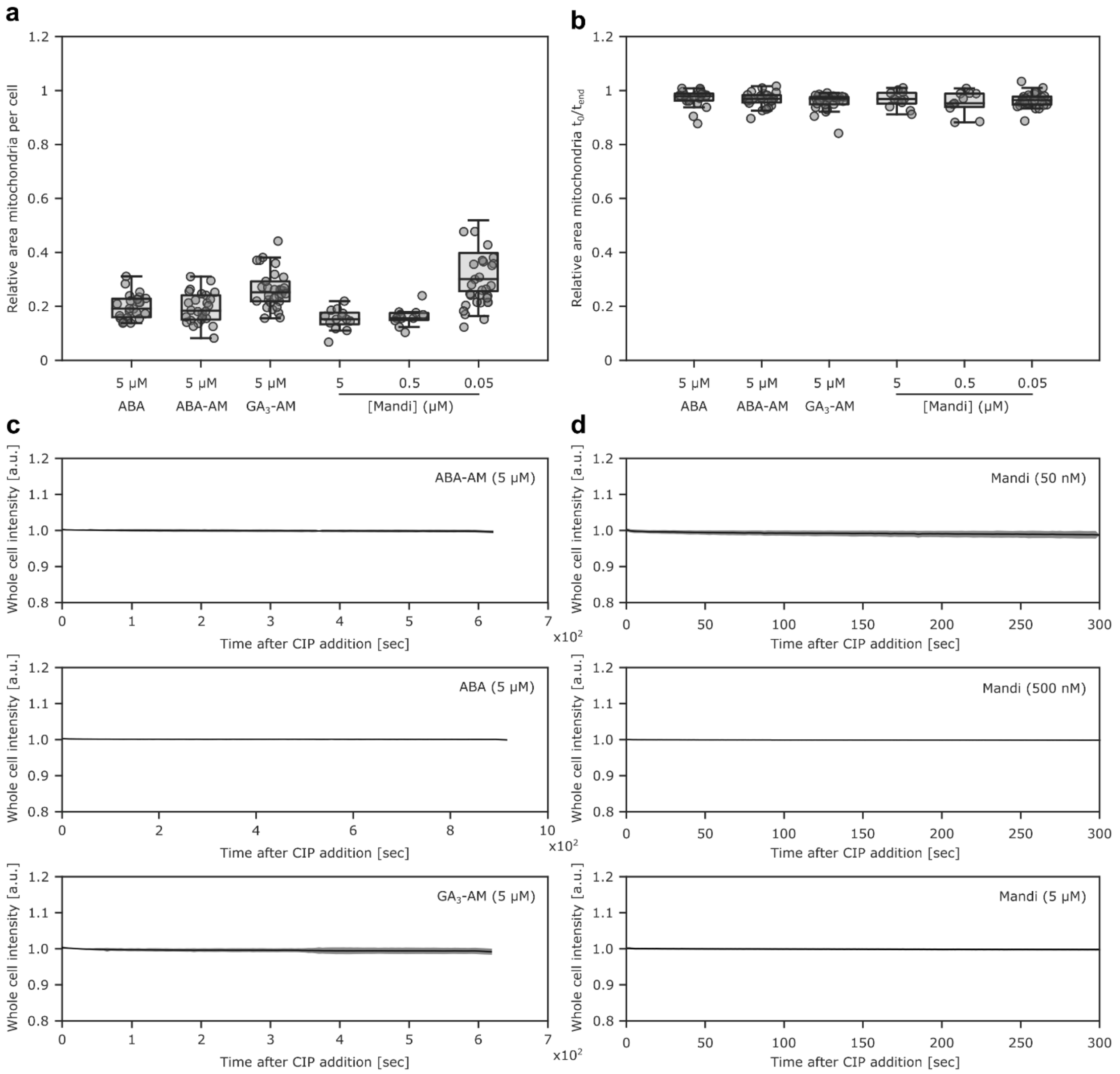

Supplementary Figure 3: Control data for CIP efficiency screening with automated microscopy. **a,** relative area occupied by mitochondria obtained from calculating the fraction of cell ROI vs. mitochondria ROI. Box plot indicates 25th percentile, median and 75th percentile. Whiskers extend to 1x interquartile distance. See supplementary table 2 for number of cells and experiments per condition. **b,** relative mitochondrial ROI size in t_0 and t_{end} image pairs obtained from thresholding Weka probability maps. Box plots as in a. **c,d,** normalized change in 488 nm intensity measured across cell ROI for all cells with a given CIP. Solid line indicates average and shaded region variation (±1 standard deviation) over time. Data in (b-d) from datasets shown in Fig. 2b,c.

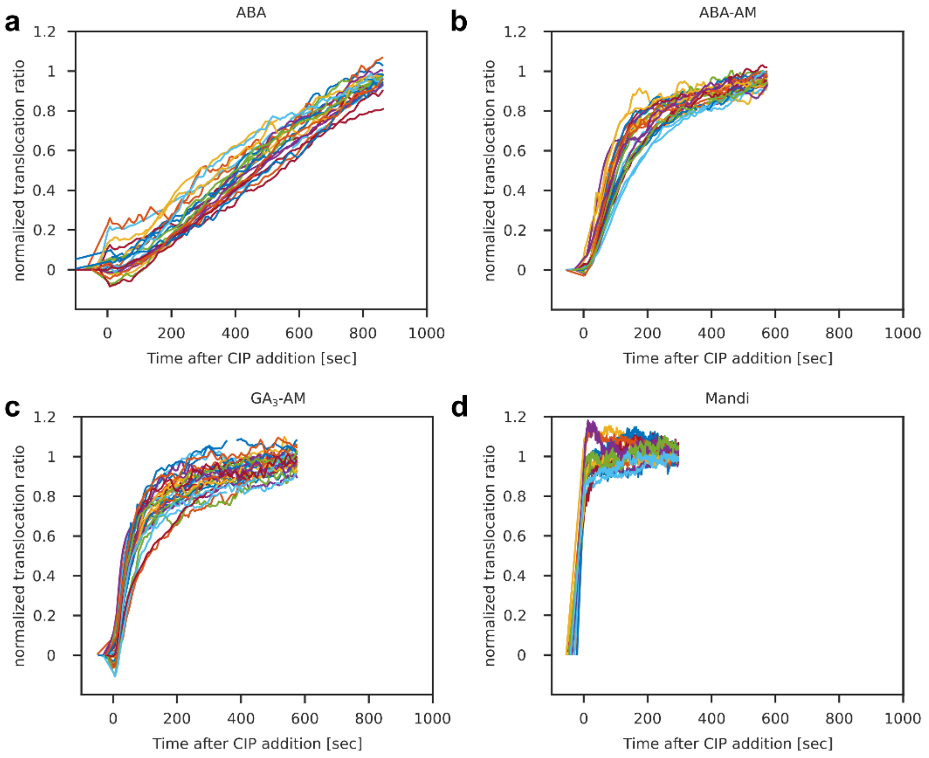

Supplementary Figure 4: Single-cell translocation ratios. a, ABA**. b,** ABA-AM**. c,** GA_3-AM **d,** Mandi**.** Each line represents photobleaching-corrected intensity ratio from mitochondria and cytosol normalized by corresponding t_0 and t_{end} values. Gaps in individual traces due to discarded frames (see methods). CIP addition at time t=0 sec.

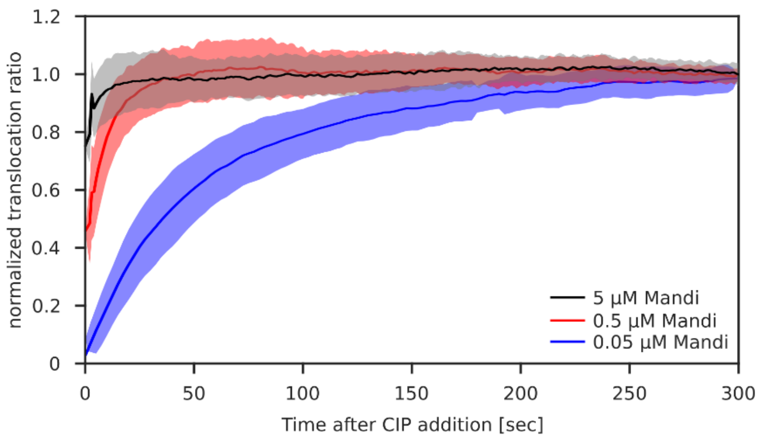

Supplementary Figure 5: Averaged translocation ratios for different Mandi concentrations. Source data used to compute translocation times $t_{0.75}$ shown in Fig. 2c,d. Solid line indicates mean and shaded region variation (±1 standard deviation) over time.

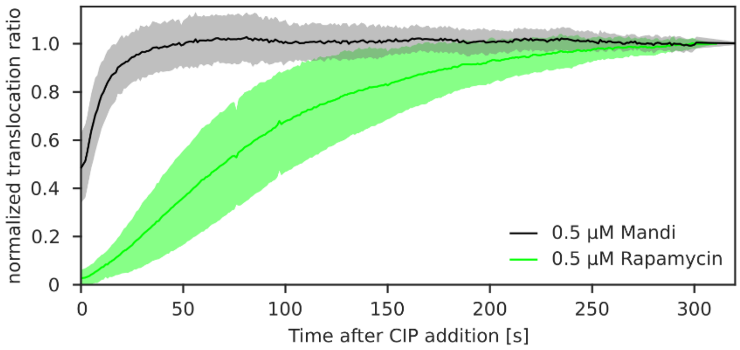

Supplementary Figure 6: Averaged translocation ratios for rapamycin and Mandi at 500 nM concentration. Ratio of cytosolic vs. mitochondrial receiver domain over time after addition of Mandi or rapamycin to 500 nM final concentration. Source data used to compute translocation times $t_{0.75}$ shown in Fig. 2d. Mean (line) ± SD (shaded region). Data for Mandi as shown in Supplementary Fig. 5. Data for rapamycin from 16 cells from 2 independent experiments.

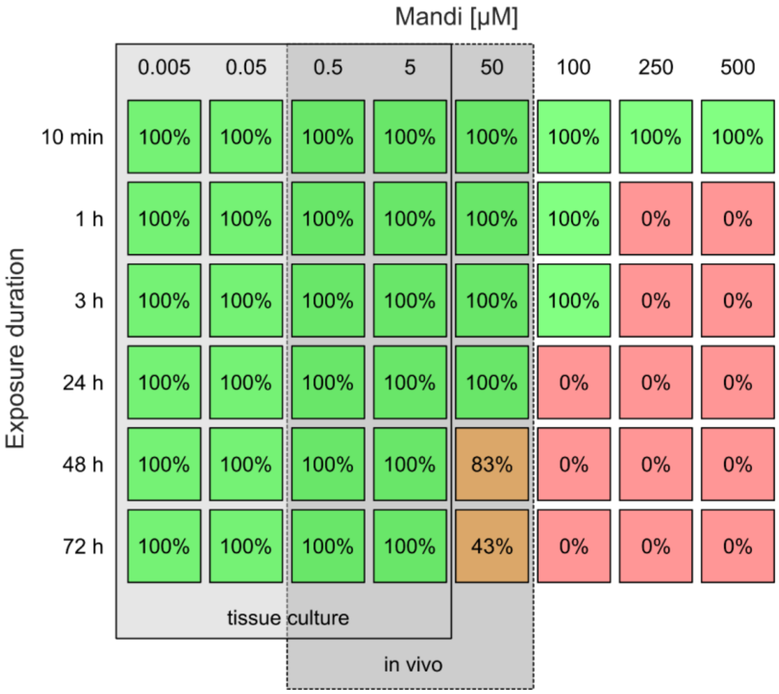

Supplementary Figure 7: Toxicological investigation of Mandi influence on zebrafish embryos. Investigation started 3-5 dpf. Survival rate summarized from 3 independent experiments using 10 embryos per condition. High concentrations of 500 µM to 50 mM showed phenotypes such as accumulation of red blood cells in the heart and slowed heartbeat.

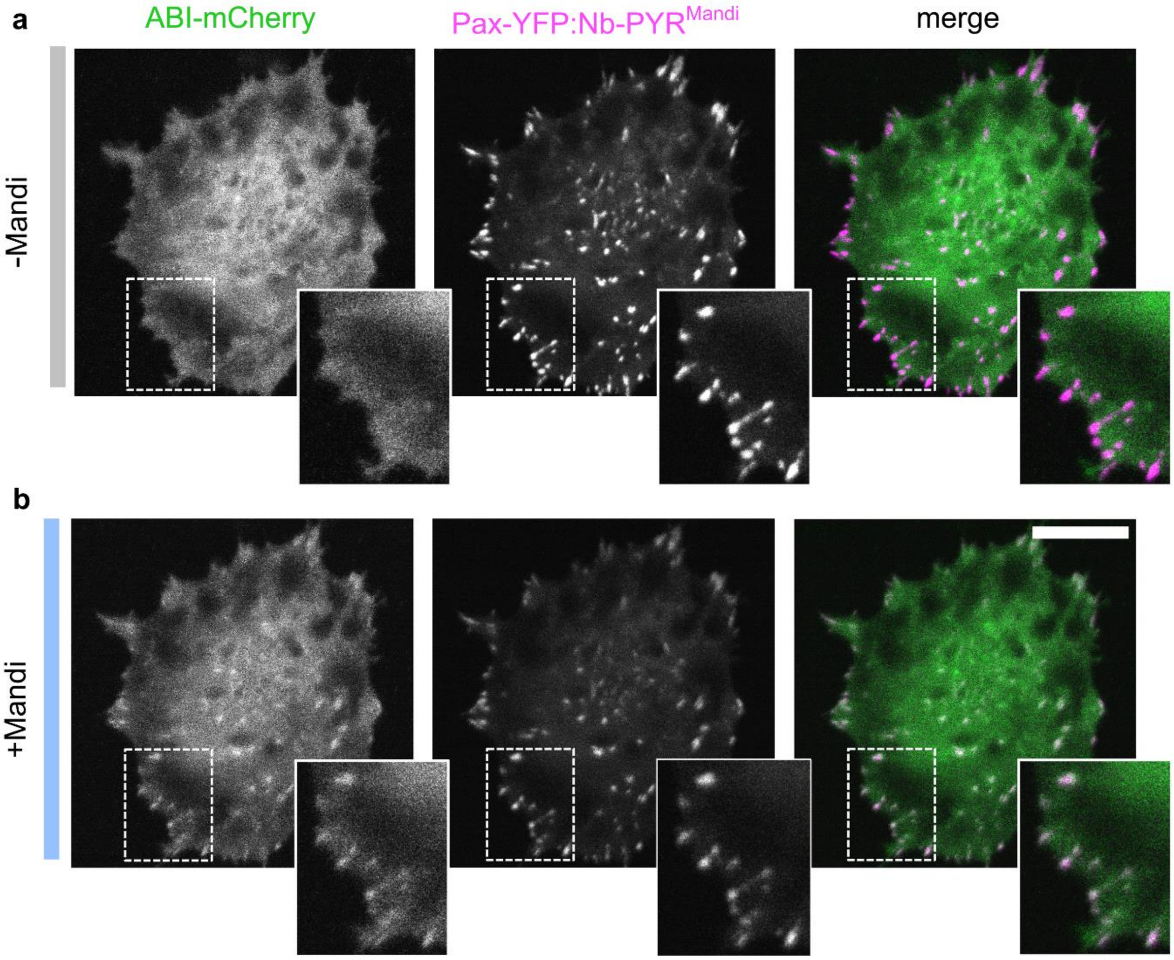

Supplementary Figure 8: **Nanobody assisted protein targeting of chemically induced protein proximity in rat embryonic fibroblast (REF) cells stably expressing paxillin-YFP. a,** Confocal fluorescence microscopy images acquired before and **b,** 5 min after addition of 50 nM Mandi. Scale bar 20 µm. Representative data for 15 cells from 2 experiments.

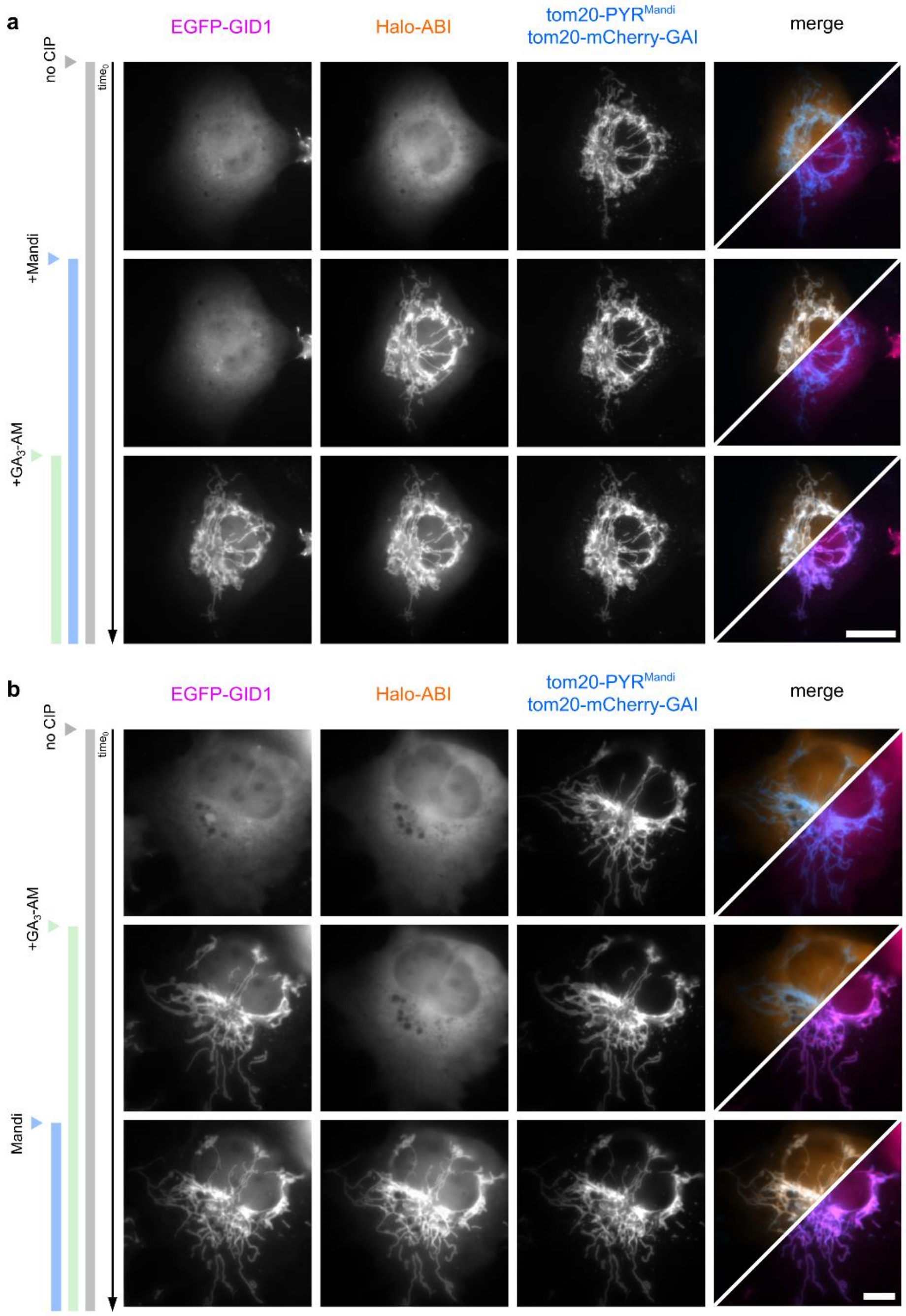

Supplementary Figure 9: Live-cell epifluorescence microscopy images to show orthogonality of Mandi and GA_3-based CIPP systems. COS-7 cells were co-transfected with TOM20-mCherry-GAI-IRES-EGFP-GID1 and TOM20-PYR^{Mandi}-IRES-Halo-ABI. Halo-tag domain was stained with HTL-SiR prior to imaging. **a,** Images acquired before CIP addition (row 1), 5 minutes after addition of Mandi (50 nM, row 2), 5 min after addition of GA_3-AM (500 nM, row 3). **b,** Images acquired before CIP addition (row 1), 5 minutes after addition of GA_3-AM (500 nM, row 2), 5 min after addition of Mandi (50nM, row 3). Scale bar 10 µm. Representative data for 3 cells.

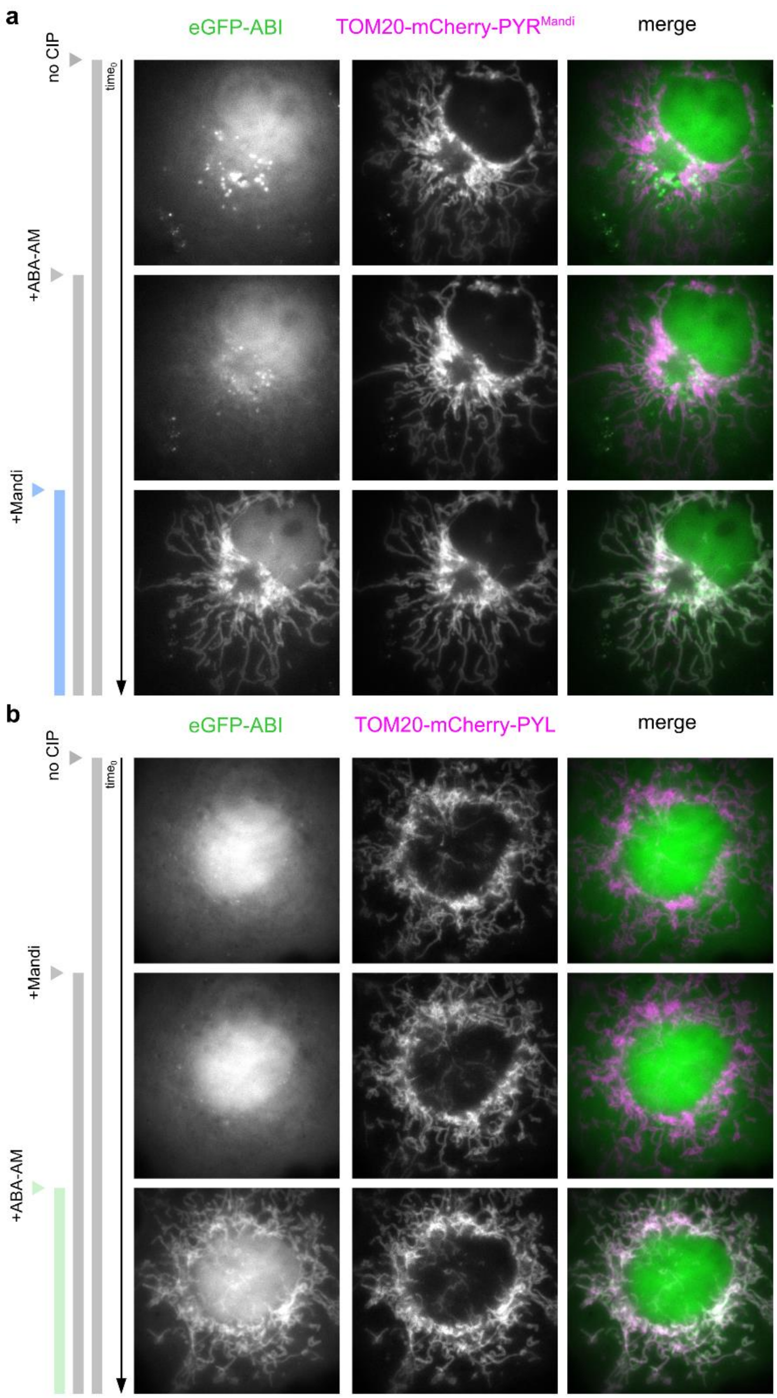

Supplementary Figure 10: Live-cell epifluorescence microscopy images to show orthogonality of Mandi and ABA- based CIPP systems. a, COS-7 cells were transfected with TOM20-mCherry-PYR^{Mandi}-IRES-EGFP-ABI. Images acquired before CIP addition (row 1), 10 minutes after addition of ABA-AM (200 nM, row 2), 10 min after addition of Mandi (200 nM, row 3). **b,** COS-7 cells were transfected with TOM20-mCherry-PYL-IRES-EGFP-ABI. Images acquired before CIP addition (row 1), 10 minutes after addition of Mandi (200 nM, row 2), 10 min after addition of ABA-AM (200 nM, row 3). Scale bar 10 µm. Representative data for 11 cells**.**

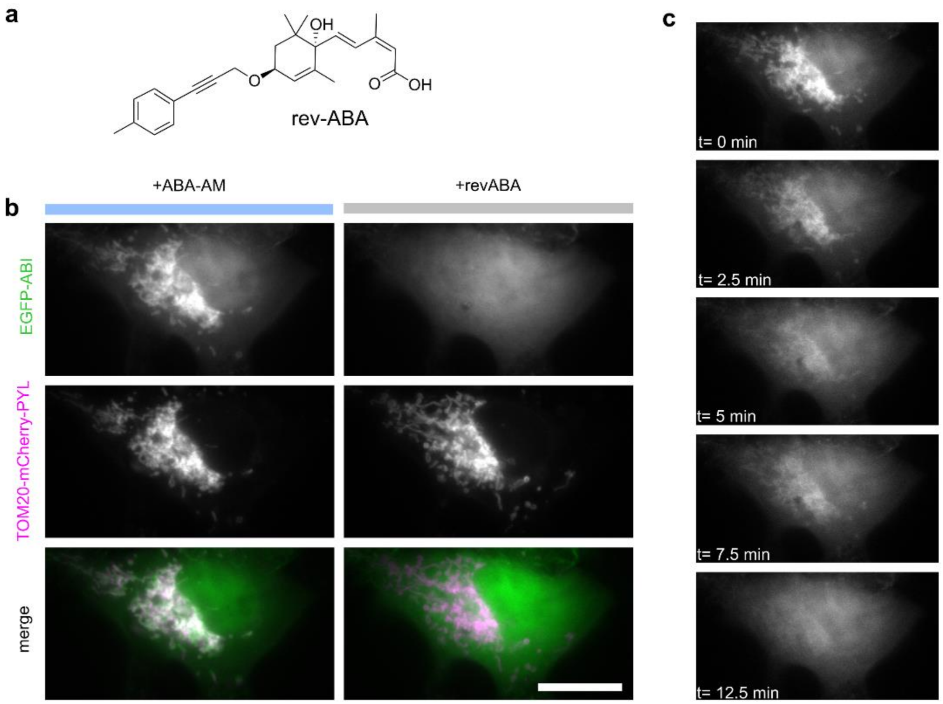

Supplementary Figure 11: revABA can reverse ABA-AM induced protein-protein interaction. a, Molecular structure of revABA. **b,** Epifluorescence microscopy images of COS-7 cells transfected with TOM20-mCherry-PYL-IRES-EGFP-ABI. Cells were incubated for 4 h with ABA-AM (200 nM) to induce dimerization. Imaging was performed in L15 with ABA-AM (200 nM). Images were acquired before and 20 min after addition of revABA (5 µM, 50x excess). **c,** Exemplary images from timelapse (Supplementary video 4). Reversion completed after ~12.5 min. Scale bar 20 µm. Representative data for 20 cells from 3 independent experiments.

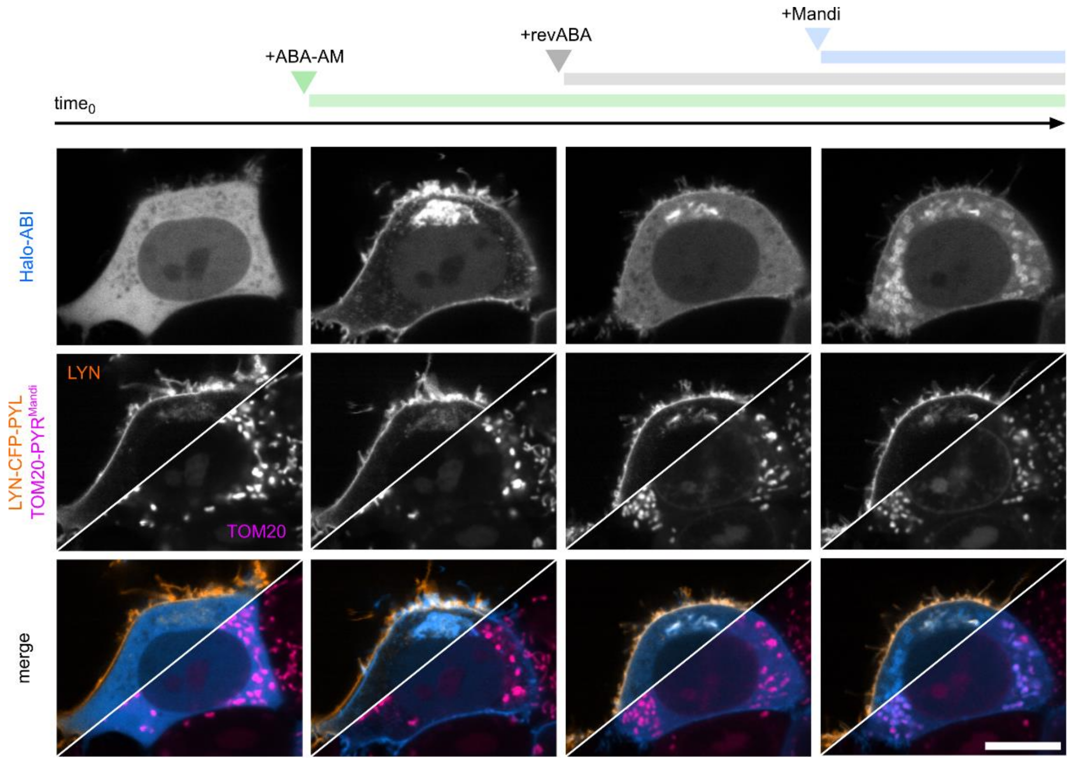

Supplementary Figure 12: Reversible and dynamic protein shuttling between mitochondria and outer membrane in living cells. HEK293T cells were co-transfected with TOM20-PYR^{Mandi}-IRES-Halo-ABI and LYN-CFP-PYL. Halo-tag domains was stained with HTL-SiR (20 nM) 2 h prior to imaging. Mitochondria were stained with MitoTracker Orange (100 nM, Thermo Fisher Scientific, USA) according to supplier protocol. Upper row shows dynamic receiver localization, middle row receptor localizations as references, lower row respective merges. Images acquired at t_0, 10 min after addition of ABA-AM (5 µM), 25 min after addition of revABA (100 µM), 10 min after addition of Mandi (5 µM). Scale bar 10 µm. Representative data for 20 cells from 2 independent experiments.

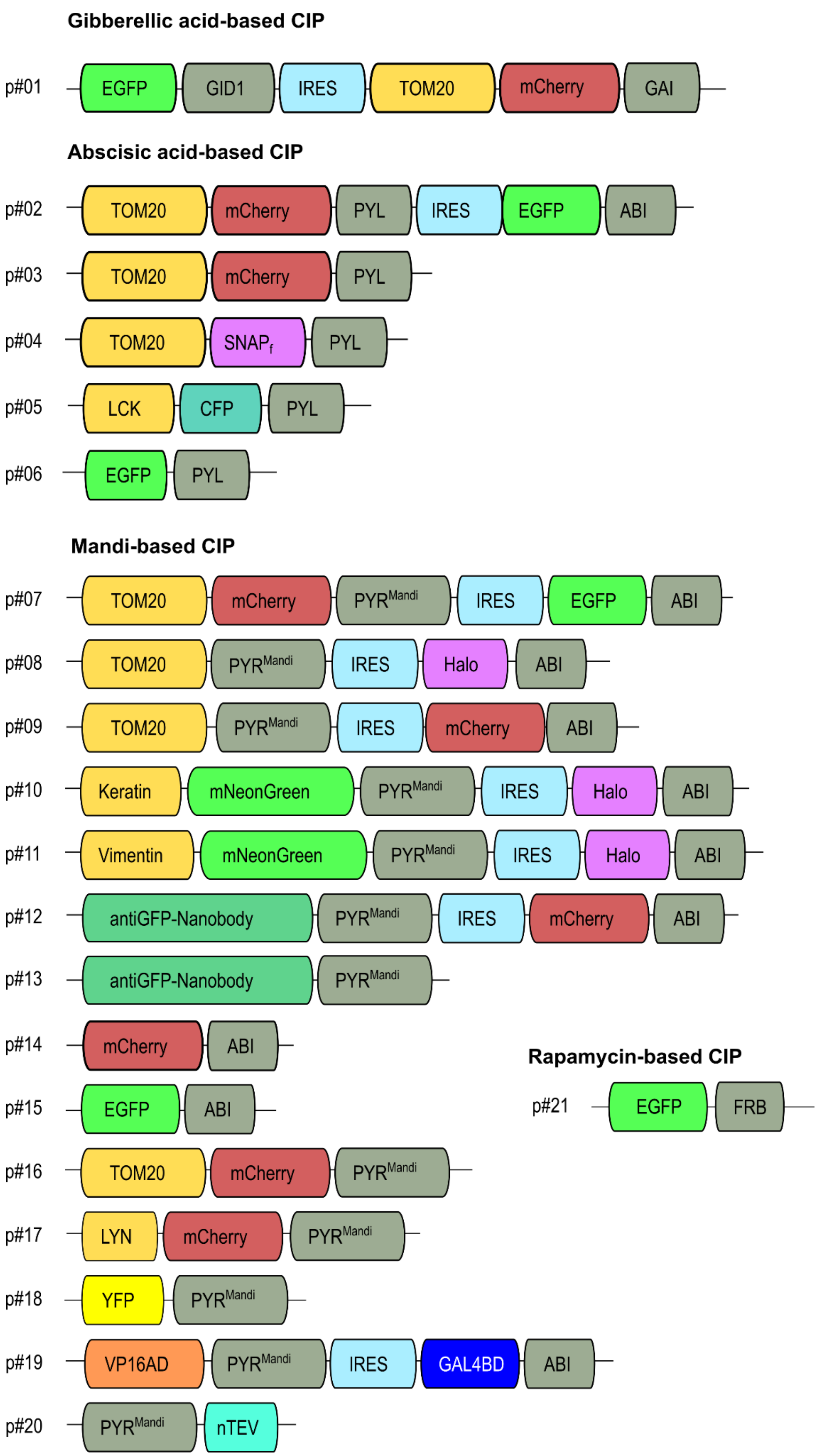

Supplementary Figure 13: List of plasmids prepared for this study

3 Supplementary References

1 Miyazono, K.-i. *et al.* Structural basis of abscisic acid signalling. *Nature* **462**, 609-614 (2009).

2 Park, S.-Y. *et al.* Agrochemical control of plant water use using engineered abscisic acid receptors. *Nature* **520**, 545-548 (2015).

3 Fink, T. *et al.* Design of fast proteolysis-based signaling and logic circuits in mammalian cells. *Nat. Chem. Biol.* **15**, 115-122 (2019).

4 Lamberth, C. *et al.* Synthesis and fungicidal activity of N-2-(3-methoxy-4-propargyloxy)phenethyl amides. Part II: Anti-oomycetic mandelamides. *Pest Manage. Sci.* **62**, 446-451 (2006).

5 Schelkle, K. M. *et al.* Light-Induced Protein Dimerization by One- and Two-Photon Activation of Gibberellic Acid Derivatives in Living Cells. *Angew. Chem., Int. Ed.* **54**, 2825-2829 (2015).

6 Takeuchi, J. *et al.* Structure-Based Chemical Design of Abscisic Acid Antagonists That Block PYL–PP2C Receptor Interactions. *ACS Chem. Biol.* **13**, 1313-1321 (2018).

7 Omar, M. A., Frey, W., Conrad, J. & Beifuss, U. Transition-Metal-Free Synthesis of Imidazo[2,1-b]thiazoles and Thiazolo[3,2-a]benzimidazoles via an S-Propargylation/5-exo-dig Cyclization/Isomerization Sequence Using Propargyl Tosylates as Substrates. *J. Org. Chem.* **79**, 10367-10377 (2014).

Supplementary Note 1: Plasmid Design and Preparation

EGFP-GID1-IRES-TOM20-mCherry-GAI was cloned from pcDNA3-PLE-IRES-GFP-GID1, which was a gift from Aline Daniel (Heidelberg University). For that, PLE and GFP-GID1 were removed by amplification of pcDNA3 backbone and IRES site with flanked primers (#1, #2, #3, #4). PLE was substituted by EGFP-GID1 which was amplified with appropriate overlaps from EGFP-GID1 (Addgene, #37306) (#5, #6). EGFP-GID1 was substituted with TOM20-mCherry-GAI fragment amplified with appropriate overlaps from TOM20-mCherry-GAI plasmid (Addgene, #37306) (#7, #8). All fragments were ligated by Gibson Assembly and sequenced using standard primers (T7-fw, IRES-rev, SP6**).**

TOM20-mCherry-PYL-IRES-EGFP-ABI was generated from pSV-ABAactDA (#38247), TOM20-mCherry-GAI (#37316) and EGFP-GID1 (#37306) all obtained from Addgene. From pSV-ABAactDA, VP16AD and GAL4DBD were removed and exchange by TOM20-mCherry and EGFP. For this purpose, two backbone fragments were amplified by PCR using flanked primers (#9, #10, #11, #12). TOM20-mCherry-GAI delivered the TOM20-mCherry fragment (#13, #14) EGFP-GID1 originated the EGFP fragment (#15, #16). All fragments were ligated by Gibson Assembly and sequenced using standard primers (IRES-gap, SV-40 for, SV-40-pA-rev, IRES-rev).

TOM20-mCherry-PYR^{Mandi}-IRES-EGFP-ABI was generated from TOM20-mCherry-PYL-IRES-EGFP-ABI. For that, the PYL side was removed by amplification of the backbone using flanked primers (#63, #64). PYR^{Mandi} gene fragment was delivered as gBlock (CTG TAC AAG AGC GCA GGA GGA CCA ATG CCA TCT GAA TTG ACC CCT GAG GAA CGC TCC GAA TTG AAA AAT TCA ATC GCC GAA TTC CAT ACC TAT CAG CTC GAC CCC GGA TCT TGC AGT TCA CTG CAT GCA CAG CGC ATC CAC GCG CCC CCA GAA TTG GTG TGG TCT ATC GTT CGC CGC TTT GAC AAA CCC CAA ACG CAC CGG CAC TTC ATA AAG TCA TGT TCA GTT GAA CAG AAT TTC GAA ATG CGA GTG GGC TGC ACC AGA GAT ATA ATA GTA ATA TCC GGT CTC CCT GCA AAT ACA TCC ACG GAG CGA CTG GAC ATA CTT GAC GAT GAA AGA AGA GTT ACG GGC GCT TCT ATA ATT GGG GGC GAA CAC CGG CTG ACT AAC TAT AAG GGC GTC ACA ACC GTT CAC CGC TTC GAG AAG GAA AAC CGC ATC TGG ACT GTA GTG TTG GAA AGC TAT GTA GTG GAT ATG CCT GAA GGA AAT TCT GAA GAC GAC ACT AGG ATG CTT GCG GAT ACA GTC GTC AAA CTT AAC CTC CAG AAA CTT GCT ACT GTA GCG GAG GCT ATG GCC CGG AAC TCA GGT GAT GGC TCT GGC AGC CAG GTC ACG TGA GGA TCC GCC CCT CTC CCT) by Integrated DNA Technologies, Inc. (IDT, Belgium). Backbone and gBlock were ligated by Gibson assembly and sequenced using standard primers (SV40 for, SV40-pA-rev, IRES rev, EGFP-N-rev).

TOM20-PYR^{Mandi}-IRES-mCherry-ABI and **TOM20-PYR^{Mandi}-IRES-HALO-ABI** were generated from TOM20-mCherry-PYR^{Mandi}-IRES-EGFP-ABI. mCherry and EGFP were removed and EGFP was substituted with mCherry or Halo. For TOM20-PYR^{Mandi}-IRES-mCherry-ABI, three fragments (backbone, PYR^{Mandi}-IRES and mCherry) were amplified from the source vector using flanked primers with appropriate overlaps (#17, #18, #19, #20, #21, #22). For TOM20-PYR^{Mandi}-IRES-HALO, the same backbone (#19, #20) and PYR^{Mandi}-IRES fragments were used, however PYR^{Mandi}-IRES fragment had to be amplified consisting and overlap to

Halo fragment (#25, #26). Halo fragment was obtained from TOM20-HALO (#27, #28), which was a gift from Dirk Ollech (Karolinska Institutet). All fragments were ligated by Gibson Assembly and sequenced using standard primers (SV40 for, SV40-pA-rev).

LCK-CFP-PYL was generated from LCK-CFP-$SNAP_f$, which was a gift from Dirk Ollech (Karolinska Institutet). $SNAP_f$ was removed by amplification of the backbone (#29, #30). PLY was amplified from TOM20-mCherry-PYL-IRES-EGFP-ABI with appropriate overlaps to the backbone fragments (#31, #32). All fragments were purified by preparative agarose gel and extracted with QIAquick Gel Extraction Kit (Qiagen). All fragments were ligated by Gibson Assembly and sequenced using standard primers (EGFP-C-F-31).

Vimentin-mNeonGreen-PYR^{Mandi}-IRES-Halo-ABI and **keratin-mNeonGreen-PYR^{Mandi}-IRES-Halo-ABI** were cloned from TOM20-PYR^{Mandi}-IRES-HALO-ABI. TOM20 side was removed by amplification of the backbone (#33, #34). Fragments of vimentin-mNeonGreen and keratin-mNeonGreen were amplified with Gibson overlaps from mNeonGreen-Keratin-17 (#35, #36) and mNeonGreen-Vimentin-7 (#37, #38), both obtained from Allele Biotech (San Diego, USA). All fragments were ligated by Gibson Assembly and sequenced using standard primers (SV40for).

TOM20-mCherry-PYL was cloned from TOM20-mCherry-PYL-IRES-EGFP-ABI. TOM20-mCherry-PLY fragment was amplified by PCR (#39,) with appropriate Gibson overlaps to pcDNA3-swap (pcDNA3 vector with swapped XhoI and XbaI restriction sides, gift from Aline Daniel (Heidelberg University)). pcDNA3-swap was cut with fast digest XhoI and XbaI enzymes (Thermo Fisher Scientific). All fragments were ligated by Gibson Assembly and sequenced using standard primers (T7, SP6).

TOM20-$SNAP_f$-PYL was cloned from TOM20-mCherry-PYL. mCherry was removed by amplification of the backbone (#41, #42). $SNAP_f$ fragment was amplified with appropriate Gibson overlaps to the backbone from LCK-CFP-$SNAP_f$, (#43, #44), which was a gift from Dirk Ollech (Karolinska Institutet). All fragments were ligated by Gibson Assembly and sequenced using standard primers (T7).

AntiGFPNanobody-PYR^{Mandi}-IRES-mCherry-ABI was cloned from TOM20-PYR^{Mandi}-IRES-mCherry-ABI. TOM20 sequence was removed by PCR amplification of backbone (#45, #46). AntiGFPNanobody insert was cloned with Gibson overlaps from pOPINE-GFP-nanobody (#47, #48), which was obtained from Addgene (#49172). All fragments were ligated by Gibson Assembly and sequenced using standard primers (SV40-for).

AntiGFPNanobody-PYR^{Mandi} and **mCherry-ABI** were cloned from AntiGFPNanobody-PYR^{Mandi}-IRES-mCherry-ABI by deletion of either IRES-mCherry-ABI (#49, #50) or AntiGFPNanobody-PYR^{Mandi}-IRES (#51, #52). Fragments were religated by Gibson Assembly and sequenced using standard primers (SV40-for).

EGFP-ABI was cloned from EGFP-GID1 (#37306). GID1 sequence was removed by PCR amplification of backbone (#53, #54). ABI insert with Gibson overlaps was amplified from TOM20-mCherry-PYR^{Mandi}-IRES-EGFP-ABI (#55, #56). Fragments were ligated by Gibson Assembly and sequenced using standard primers (EGFP-for).

TOM20-mCherry-PYR^{Mandi} was cloned from EGFP-GID1 (#37306). EGFP-GID1 sequence was removed by PCR amplification of backbone (#54, #57). PYR^{Mandi} insert with Gibson overlaps was amplified from TOM20-mCherry-PYR^{Mandi}-IRES-EGFP-ABI (#58, #59). Fragments were ligated by Gibson Assembly and sequenced using standard primers (EGFP-for).

LYN-mCherry-PYR^{Mandi} was cloned from TOM20-mCherry-PYR^{Mandi}. TOM20 sequence was removed by PCR amplification of backbone (#57, #60). LYN fragment with Gibson overlaps was generated by hybridization of two complementary primers (#61, #62) for 5 min at 95°C. Fragments were ligated by Gibson Assembly and sequenced using standard primers (CMV-for).

pEGFP-PYL was cloned from EGFP-ABI. ABI sequence was removed by PCR amplification of backbone (#65, #66). PYL fragment with appropriate Gibson overlaps was amplified from tom20-mCherry-PYL-IRES-EGFP-ABI (#67, #68). Fragments were ligated by Gibson Assembly and sequenced using standard primers (EGFP-for).

pYFP-PYR^{Mandi} was cloned from LYN-mCherry-PYR^{Mandi}. LYN-mCherry sequence was removed by PCR amplification of backbone (#69,#70). YFP fragment with appropriate Gibson overlaps (#71, #72) was amplified from pcDNA3-LAP2-YFP-NLS (#25847). Fragments were ligated by Gibson Assembly and sequenced using standard primers (CMV-for).

VP16AD-PYR^{Mandi}-IRES-GAL4BD-ABI was cloned from SV-ABAactDA (#38247). PYL sequence was removed by PCR amplification of backbone (#73, #74). PYR^{Mandi} fragments with appropriate Gibson overlaps (#75, #76) was amplified from TOM20-mCherry-PYR^{Mandi}-IRES-EGFP-ABI. Fragments were ligated by Gibson Assembly and sequenced using standard primers (IRES rev).

PYR^{Mandi}-nTEV was cloned from PYL-nTEVp (#119213). PYL sequence was removed by PCR amplification of backbone (#77, #78). PYR^{Mandi} fragments with appropriate Gibson overlaps (#79, #80) was amplified from TOM20-mCherry-PYR^{Mandi}-IRES-EGFP-ABI. Fragments were ligated by Gibson Assembly and sequenced using standard primers (CMV-for).

EGFP-FRB was cloned from pEGFP-FRB^{*T2098L} (#25919) by PCR amplification (#81, #82) followed by ligation using site directed mutagenesis kit (New England Biolabs, USA). Plasmid was sequenced using standard primers (Eurofins, Luxembourg - cmv-f, pEGFP C2-RP).

Supplementary Table 3: List of all primers used in this study.

Primer	Sequence (5’-3’)
#1	TAA ATG CAG AAC GGC CCC GGT GCT C
#2	CAT GGT GGC GGC CGC AAT TGC GCT AG
#3	TAA ACC GGA ATT CCG
#4	CAT GGT TGT GGC CAT ATT ATC ATC

#5	GCA ATT GCG GCC GCC ACC ATG GTG AGC AAG GGC GAG GAG CTG
#6	GAG GGG CGG AAT TCC GGT TTA ACA TTC CGC GTT TAC AAA CGC
#7	GAT AAT ATG GCC ACA ACC ATG GGT CGG AAC AGC GCC ATC
#8	GCA ATT GCG GCC GCC ACC ATG GTG AGC AAG GGC GAG GAG
#9	ACG CGT GTG CCT TTG TAT GGT
#10	CAT GAA TTC CGA AAA TGG ATA
#11	CCA ACT CAA GAC GAA TTC ACC
#12	CAT GAG CTC GGC CAT ATT ATC
#13	TAT CCA TTT TCG GAA TTC ATG CCA CCA TGG ATG GGT CGG AAC
#14	GGT GAA TTC GTC TTG AGT TGG TCC TCC TGC GCT CTT GTA CAG
#15	GAT AAT ATG GCC GAG CTC ATG GTG AGC AAG GGC GAG GAG CTG
#16	ACC ATA CAA AGG CAC ACG CGT TCC TCC TGC GCT CTT GTA CAG
#17	TGG TCC TCC TGC GCT CTT GTA CAG
#18	TGA GGA TCC GCC CCT CTC CCT
#19	GTG CCT TTG TAT GGT TTT AC
#20	GAA GTT GGG GTC ACT TCG TC
#21	CGA AGT GAC CCC AAC TTC AGC GCA GGA GGA CCA ATG
#22	TCC GGA TCC CAT GAG CTC GGC CAT ATT ATC ATC C
#23	GAG CTC ATG GGA TCC GGA GCA AGT GGA AT
#24	AGT AAA ACC ATA CAA AGG CAC TGG TCC TCC TGC GCT CTT GT
#25	CGA AGT GAC CCC AAC TTC AGC GCA GGA GGA CCA ATG C
#26	TCG GAT CCC ATG AGC TCG GCC ATA TTA T
#27	AGC TCA TGG GAT CCG AAA TCG GTA CTG G
#28	AGT AAA ACC ATA CAA AGG CAC ACC GGA AAT CTC CAG AGT AG
#29	GGT GAA TTC ACC GGT ACC TG
#30	TGA GCG GCC GCA TAG ATA AC
#31	GGT ACC GGT GAA TTC ACC ACT CAA GAC GAA TTC ACC CA
#32	CTA TGC GGC CGC TCA GTT CAT AGC TTC AGT GAT CG
#33	AGC GCA GGA GGA CCA ATG
#34	CCA TGG TGG CAT GAA TTC CG
#35	GAA TTC ATG CCA CCA TGG ATG TCC ACC AGG TCC GTG TC
#36	TGG TCC TCC TGC GCT CTT GTA CAG CTC GTC CAT GC
#37	GAA TTC ATG CCA CCA TGG ATG AGC TTC ACC ACT CGC TC
#38	TGG TCC TCC TGC GCT CTT GTA CAG CTC GTC CAT GC
#39	TCA CAC TGG CGG CCG TCT AGA ATG GGT CGG AAC AGC GCC ATC
#40	TAT AGA ATA GGG CCC CTC GAG TCA AGC GTA ATC TGG AAC ATC
#41	AGC GCA GGA GGA CCA ACT CA
#42	TCC ACT TGC TCC GGA TCC GA
#43	TCC GGA GCA AGT GGA ATG GAC AAA GAC TGC GAA ATG AA
#44	TGG TCC TCC TGC GCT ATT AAC CTC GAG TTT AAA CGC GG
#45	AGC GCA GGA GGA CCA ATG CC
#46	CCA TGG TGG CAT GAA TTC CG
#47	GAA TTC ATG CCA CCA TGG ATG CAG GTT CAA CTG GTG GA
#48	TGG TCC TCC TGC GCT TTT AGA GCT CAC CGT CAC CTG
#49	GAT TAC AAG GAC GAT GAC GAT AAG TGA AGC GGC

#50	ATC CTC ACG TGA CCT GGC TGC CAG AGC CAT CAC CT
#51	ATT CAT GCC ACC ATG GAT GGT GAG CAA GGG CGA GGA
#52	CCA TGG TGG CAT GAA TTC CGA AAA TGG ATA TA
#53	CTT GTA CAG CTC GTC CAT GC
#54	CTG ATC ATA ATC AGC CAT ACC ACA
#55	TGG ACG AGC TGT ACA AGA GCG CAG GAG GAA CGC GT
#56	TGG TAT GGC TGA TTA TGA TCA GTC ACT TAT CGT CAT CGT CCT TGT
#57	CAT GGT GGC GAC CGG TAG
#58	CCG GTC GCC ACC ATG GGT CGG AAC AGC GCC ATC
#59	TGG TAT GGC TGA TTA TGA TCA GTC ACG TGA CCT GGC TGC CAG A
#60	GGA TCC GGA GCA AGT GGA AT
#61	CCG GTC GCC ACC ATG GGA TGT ATT AAA TCA AAA AGG AAA GAC GGA TCC GGA GCA AGT
#62	ACT TGC TCC GGA TCC GTC TTT CCT TTT TGA TTT AAT ACA TCC CAT GGT GGC GAC CGG
#63	TGG TCC TCC TGC GCT CTT GTA CAG
#64	TGA GGA TCC GCC CCT CTC CCT
#65	CTT TAC AGC TCG TCC ATG CCG AG
#66	ATT ACG CTT GAA GCG GCC GCG ACT CTA GAT
#67	TGG ACG AGC TGT ACA AGG GAT CCG GAG CAA GTG GAA
#68	GCC GCT TCA AGC GTA ATC TGG AAC ATC
#69	AGC GCA GGA GGA CCA ATG C
#70	CAT GGT GGC GAC CGG TAG
#71	TGG TCC TCC TGC GCT CTT GTA TAG CTC GTC CAT GCC G
#72	CCG GTC GCC ACC ATG GTG AGC AAG GGC GAG GAG
#73	AGG TCA CGT GAG GAT CCG CCC CTC TCC CT
#74	GAT GGC ATT GGC GCG CCC CCA CCG TAC T
#75	CGC GCC AAT GCC ATC TGA ATT GAC CCC T
#76	GGA TCC TCA CGT GAC CTG GCT GCC AGA
#77	GAT GGC ATT GGC GCG CCC CCA CCG ATG T
#78	GGT CAC GGG ATC CGG AAG TGG AGA AAG C
#79	CGC GCC AAT GCC ATC TGA ATT GAC CCC T
#80	CCG GAT CCC GTG ACC TGG CTG CCA GA
#81	CAA GGA CCT CAC TCA AGC CTG GG
#82	ACA TTC CCT GAT TTC ATG

Supplementary Note 2: Synthesis and Characterization

Mandipropamid (1)

There are multiple sources of mandipropamid (**1**). It can be purchased as pure compound from common suppliers (e.g., Sigma Aldrich analytical standard PESTANAL®) or synthesized according to literature[4]. Here, we present an alternative method to access mandipropamid at the gram scale: the extraction and isolation of mandipropamid from Revus TOP®, an agrochemical marketed by Syngenta.

*Extraction of Mandipropamid **(1)** from Revus TOP®*

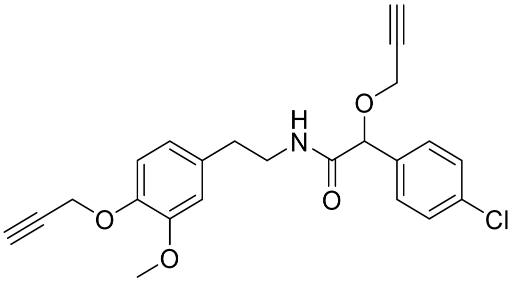

Mandipropamid was isolated from the commercially available suspension concentrate Revus TOP®, which contains mandipropamid and difenoconazol. 10 g Revus TOP® was suspended in 50 ml H_2O and extracted 10-fold with 50 ml DCM. The yellow extract was dried over Na_2SO_4 and concentrated under reduced pressure. The crude product was purified by column chromatography (SiO_2, DCM:EA- 9:1) yielding in an off-white solid (2.2 g, 5.34 mmol).

^{1}H NMR (300 MHz, $CDCl_3$) δ 7.37 – 7.26 (m, 4H), 6.97 (d, *J* = 7.9 Hz, 1H), 6.70 (m, 3H), 4.96 (s, 1H), 4.78 – 4.74 (m, 2H), 4.11 (m, 2H), 3.83 (s, 3H), 3.54 (m, 2H), 2.79 (t, *J* = 6.7 Hz, 2H), 2.51 (t, *J* = 2.4 Hz, 1H), 2.48 (t, *J* = 2.4 Hz, 1H).

^{13}C NMR (75 MHz, $CDCl_3$) δ 169.5 (s, 1C), 149.8 (s, 1C), 145.4 (s, 1C), 134.7 (s, 1C), 134.6 (s, 1C), 132.7 (s, 1C), 128.8 (s, 2C), 128.7 (s, 2C), 120.6 (s, 1C), 114.7 (s, 1C), 112.4 (s, 1C), 79.7 (s, 1C), 75.8 (s, 1C), 75.7 (s, 1C), 56.9 (s, 2C), 56.4 (s, 2C), 55.7 (s, 1C), 40.2 (s, 1C), 35.2 (s, 1C).

HR-ESI$^{+:}$ m/z calcd. for $[C_{23}H_{22}ClNO_4+Na]^+$: 434.1130; found: 434.1123.

Synthesis of acetoxymethyl CIP

*(+)-Abscisic acid acetoxymethyl ester (ABA-AM, **4**)*

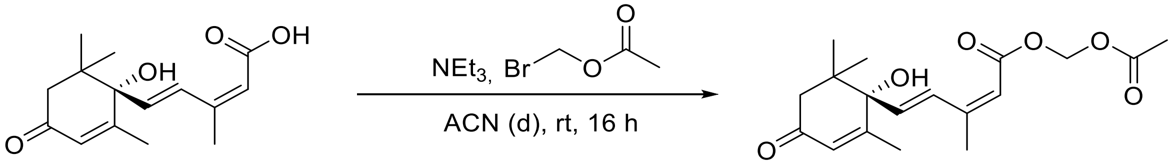

A flame-dried Schlenk tube was charged with (+)-abscisic acid (**3**,10.8 mg, 40.8 µmol, 1 eq) dissolved in 1 ml anhydrous acetonitrile. After addition of bromomethyl acetate (5.2 µl, 8.1 mg, 53.0 µmol, 1.3 eq) and triethylamine (7.0 µl, 5.4 mg, 53.0 µmol, 1.3 eq) the reaction mixture was stirred overnight at room temperature. The crude product was concentrated under reduced pressure and purified by column chromatography (SiO_2, Cy:EA- 2:1) yielding in an off-white resin (8.8 mg, 28.1 µmol, 69 %).

^{1}H NMR (500 MHz, CD_3OD) δ 7.79 (dd, J = 16.1, 0.9 Hz, 1H), 6.34 (dd, J = 16.1, 0.7 Hz, 1H), 5.94 (t, J = 1.3 Hz, 1H), 5.76 – 5.75 (m, 1H), 5.75 (s, 2H), 2.53 (d, J = 16.9 Hz, 1H), 2.20 (d, J = 16.4 Hz, 1H), 2.11 – 2.04 (m, 6H), 1.93 (s, 3H), 1.07 (s, 3H), 1.02 (s, 3H).

^{13}C NMR (126 MHz, $CDCl_3$) δ 197.7 (s, 1C), 170.1 (s, 1C), 164.3 (s, 1C), 162.3 (s, 1C), 152.1 (s, 1C), 137.6 (s, 1C), 128.0 (s, 1C), 127.3 (s, 1C), 117.2 (s, 1C), 79.8 (s, 1C), 79.1 (s, 1C), 49.9 (s, 1C), 41.7 (s, 1C), 24.5 (s, 1C), 23.2 (s, 1C), 21.5 (s, 1C), 21.0 (s, 1C), 19.0 (s, 1C)

HR-ESI^{+}: m/z calcd. for $[C_{18}H_{24}O_6+Na]^+$: 359.1465; found: 359.1477.

*Gibberellic acid acetoxymethyl ester (GA$_3$-AM, **2**)*

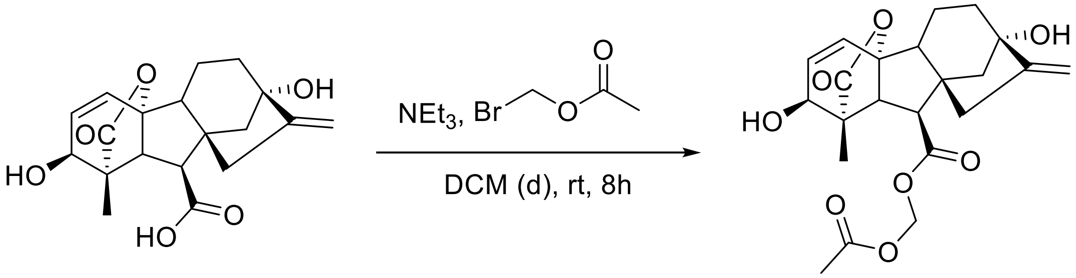

GA$_3$-AM was prepared according to procedures described in previous publications[5].

Synthesis of abscisic acid antagonist revABA (PANMe)

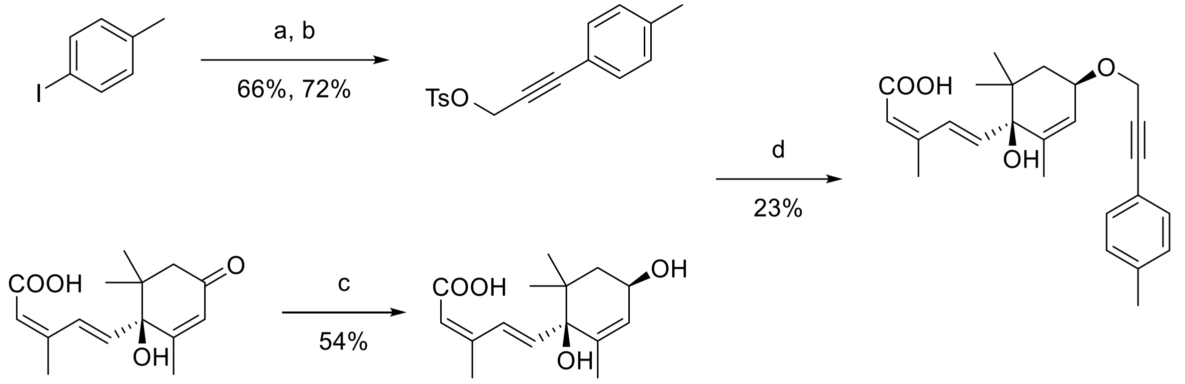

Scheme S 1: Route for synthesis of ABA antagonist revABA[6]. a, propargylalcohol, $(Ph_3P)_2PdCl_2$, CuI, DIPEA, THF; **b,** TsCl, KOH, Et2O; **c:** NaBH4, $CeCl_3$*7H_2O, MeOH **d,** NaH, THF.

(p-Tolyl)prop-2-yn-1-ol

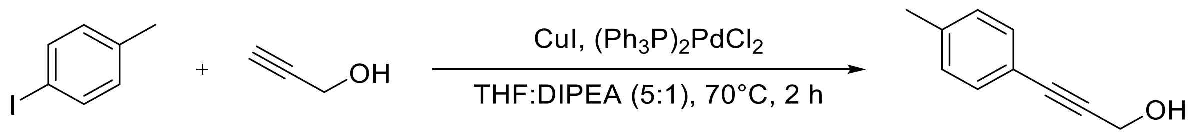

p-Iodo toluene (1.0 g, 4.6 mmol, 1 eq) was dissolved in a mixture of 5 ml THF and 1 ml DIPEA. After addition of propargyl alcohol (300 µl, 5.50 mmol, 1.2 eq) the mixture was degassed by bubbling argon through the solution for 10 min. After addition of copper(I) iodide (43.7 mg, 229.3 µmol, 0.05 eq) and bis(triphenylphosphine)palladium(II) dichloride (64.4 mg, 91.7 µmol, 0.02 eq) the reaction mixture was heated to 70°C and stirred for 2 h. The reaction was quenched with a 1 M aqueous solution of HCl and extracted three times with DCM. The organic layer was dried over Na_2SO_4 and concentrated under reduced pressure. The crude product was purified by column chromatography (SiO_2, Cy:EA- 9:1) yielding a brown oil (446.8 mg, 3.06 mmol, 67%). The measured NMR spectrum was in accordance with literature reported spectra[6].

^{1}H NMR (300 MHz, $CDCl_3$) δ 7.33 (d, *J* = 8.0 Hz, 2H), 7.12 (d, *J* = 8.0 Hz, 2H), 4.49 (d, *J* = 6.1 Hz, 2H), 2.34 (s, 3H), 1.65 (t, *J* = 6.1 Hz, 1H).

^{13}C NMR (75 MHz, $CDCl_3$) δ 138.23 (s, 1C), 131.15 (s, 2C), 128.64 (s, 2C), 118.98 (s, 1C), 86.04 (s, 1C), 85.45 (s, 1C), 51.31 (s, 1C), 21.05 (s, 1C).

3-(p-Tolyl)prop-2-yn-1-yl 4-methylbenzenesulfonate

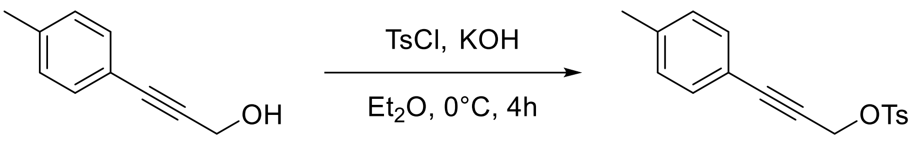

According to Omar *et al.*[7], tosyl chloride (26.1 mg, 136.8 µmol, 1 eq) and (*p*-tolyl)prop-2-in-1-ol (20.0 mg, 136.8 µmol, 1 eq) were dissolved in 5 ml diethyl ether and cooled to 0 °C. Freshly grinded KOH (76.8 mg, 1,37 mmol, 10 eq) was slowly added and the reaction was stirred for further 4 h at 0 °C. After TLC showed full conversion, the reaction mixture was quenched with ice water and extracted with diethyl ether. The combined organic layer was dried over Na_2SO_4 and concentrated under reduced pressure. The product was obtained as a brown oil (29.9 mg, 99.5 µmol, 73%). The measured NMR spectrum was in accordance with literature and the product was directly used without further purification.

^{1}H NMR (300 MHz, Chloroform-*d*) δ 7.85 (d, *J* = 8.1 Hz, 2H), 7.31 (d, *J* = 8.0 Hz, 2H), 7.15 (d, *J* = 8.1 Hz, 2H), 7.08 (m, *J* = 8.0 Hz, 2H), 4.94 (s, 2H), 2.40 (s, 3H), 2.34 (d, *J* = 3.4 Hz, 3H).

^{13}C NMR (126 MHz, Chloroform-d) δ 144.9 (s, 1C), 139.3 (s, 1C),133.4 (s, 1C), 131.7 (s, 2C), 129.8 (s, 2C), 129.0 (s, 2C), 128.2 (s, 2C), 118.3 (s, 1C), 89.2 (s, 1C), 79.9 (s, 1C), 58.8 (s, 1C), 21.6 (s, 1C), 21.5 (s, 1C).

HR-ESI$^{+:}$ m/z calcd. for $[C_{17}H_{16}O_3S+Na]^+$: 323.0718; found: 323.0724.

1.1.1.1 ABA-(S)-OH

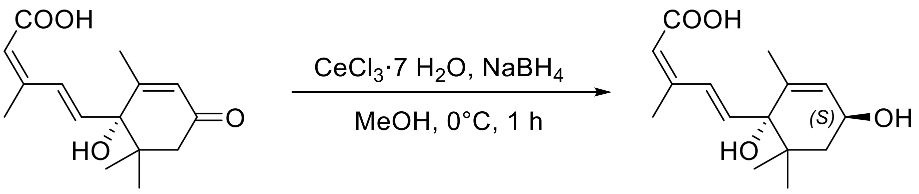

According to Takeuchi *et al.*[6], a flame-dried Schlenk flask was equipped with abscisic acid **3**(50 mg, 189.2 µmol, 1 eq) and dissolved in 4 ml dry methanol. The solution was cooled to 0°C and cerium(III) chloride heptahydrate (211.4 mg, 567.5 µmol, 3 eq) and sodium borohydride (15.1 mg, 399.1 µmol, 2.1 eq) were added. The reaction mixture was stirred for further 60 min and quenched with a saturated solution of ammonium chloride. After concentration of the solution in vacuo to remove methanol, the reaction mixture was acidified to pH 2 by addition of aqueous 1 M HCl and extracted with DCM. The combined organic layer was washed with brine, dried over Na_2SO_4 and concentrated under reduced pressure. The crude product was purified by column chromatography (SiO_2, Cy:EA- 3:1), yielding in a colorless, cloudy oil (31.3 mg, 117.5 µmol, 62 %).

^{1}H NMR (500 MHz, Methanol-*d4*) 7.79 – 7.60 (m, 1H), 6.21 (d, J = 16.1 Hz, 1H), 5.69 (d, J = 1.6 Hz, 1H), 5.52 (dt, J = 2.8, 1.4 Hz, 1H), 4.18 (ddt, J = 10.4, 6.4, 2.2 Hz, 1H), 2.01 (d, J = 1.3 Hz, 3H), 1.71 (m, 1H), 1.64 (s, 3H), 1.63 – 1.55 (m, 1H), 1.01 (s, 3H), 0.90 (s, 3H).

^{13}C NMR (126 MHz, Methanol-*d4*) δ 169.7 (s, 1C), 152.0 (s, 1C), 141.4 (s, 1C), 139.5 (s, 1C), 128.8 (s, 1C), 128.0 (s, 1C), 118.4 (s, 1C), 80.3 (s, 1C), 66.4 (s, 1C), 44.9 (s, 1C), 41.0 (s, 1C), 25.6 (s, 1C), 23.2 (s, 1C), 21.4 (s, 1C), 18.2 (s, 1C).

HR-ESI$^{+:}$ m/z calcd. for $[C_{15}H_{22}O_4+Na]^+$: 289.1416; found: 289.1423.

ABA antagonist revABA ***7***

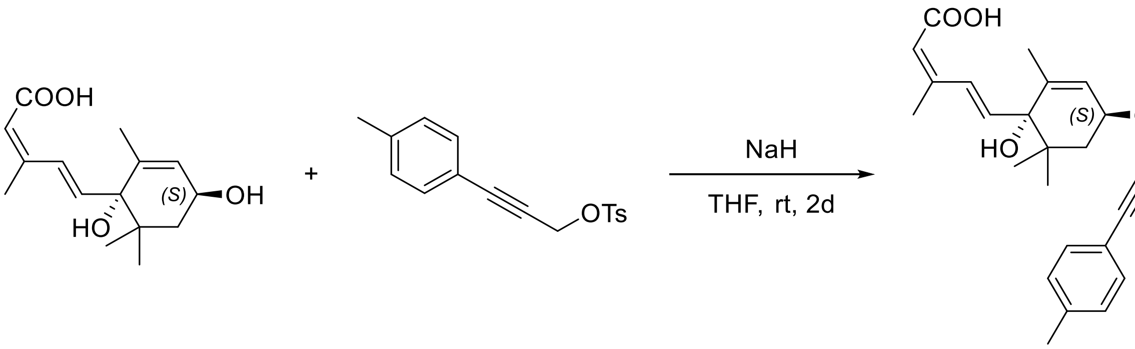

According to literature[6], a flame-dried Schlenk tube was equipped with sodium hydride (10.2 mg, 60 wt%, 255.3 µmol, 3.4 eq), which was suspended in 5 ml dry THF, cooled to 0°C. ABA-(*S*)-OH (20.0 mg, 75.1 µmol, 1 eq) was added and the solution was stirred for 15 min. In a separate flame-dried Schlenk tube, 3-(*p*-Tolyl)prop-2-in-1-yl 4-methylbenzolsulfonat (28.2 mg, 80 wt%, 75.1 µmol, 1 eq))was dissolved in 2 ml dry THF and slowly added to the reaction mixture. The reaction was allowed to warm up to room temperature and stirred for 2 d under an argon atmosphere. As the reaction did not show full conversion according TLC, another 6.8 eq NaH (20.4 mg, 0.51 mmol) were added to the reaction mixture and the reaction was stirred for further 16 h at 50°C. The reaction was quenched with 1 M HCl solution and extracted with DCM. The organic layer was washed with brine, dried over Na_2SO_4 and concentrated under reduced pressure. The product was purified by column chromatography (SiO_2, Cy:EA 0-50%) and obtained as a yellowish resin (6.8 mg, 17.2 µmol, 23%). For live cell experiments a batch of revABA was further purified by preparative reverse phase HPLC (C18, H_2O/MeCN gradient +0.1% TFA).

^{1}H NMR (500 MHz, DMSO-*d*$_6$) δ 7.62 (d, *J* = 15.9 Hz, 1H), 7.32 (d, *J* = 7.8 Hz, 2H), 7.19 (d, *J* = 7.8 Hz, 2H), 6.08 (d, *J* = 15.9 Hz, 1H), 5.63 (s, 1H), 5.57 (s, 1H), 4.39 (d, *J* = 1.5 Hz, 2H), 4.13 (m, 1H), 2.31 (s, 3H), 1.94 (d, *J* = 1.1 Hz, 3H), 1.78 (m, 1H) 1.57 (m, 4H), 0.93 (s, 3H), 0.84 (s, 3H).

^{13}C NMR (126 MHz, DMSO-*d*$_6$) δ 166.9 (s, 1C), 149.4 (s, 1C), 140.3 (s, 1C), 139.2 (s, 1C), 138.4 (s, 1C), 131.2 (s, 1C), 129.3 (s, 2C), 125.9 (s, 2C), 123.8 (s, 1C), 119.0 (s, 1C), 117.7 (s, 1C), 86.1 (s, 1C), 85.2 (s, 1C), 77.6 (s, 1C), 71.9 (s, 1C), 55.3 (s, 1C), 40.1 (s, 1C), 39.3 (s, 1C), 25.2 (s, 1C), 22.9 (s, 1C), 20.9 (s, 2C), 17.9 (s, 1C).

HR-ESI$^{+:}$ m/z calcd. for $[C_{25}H_{30}O_4+Na]^+$: 417.2042; found: 417.2041.

NMR Characterization

*Mandipropamid (**1**)*

^{1}H NMR

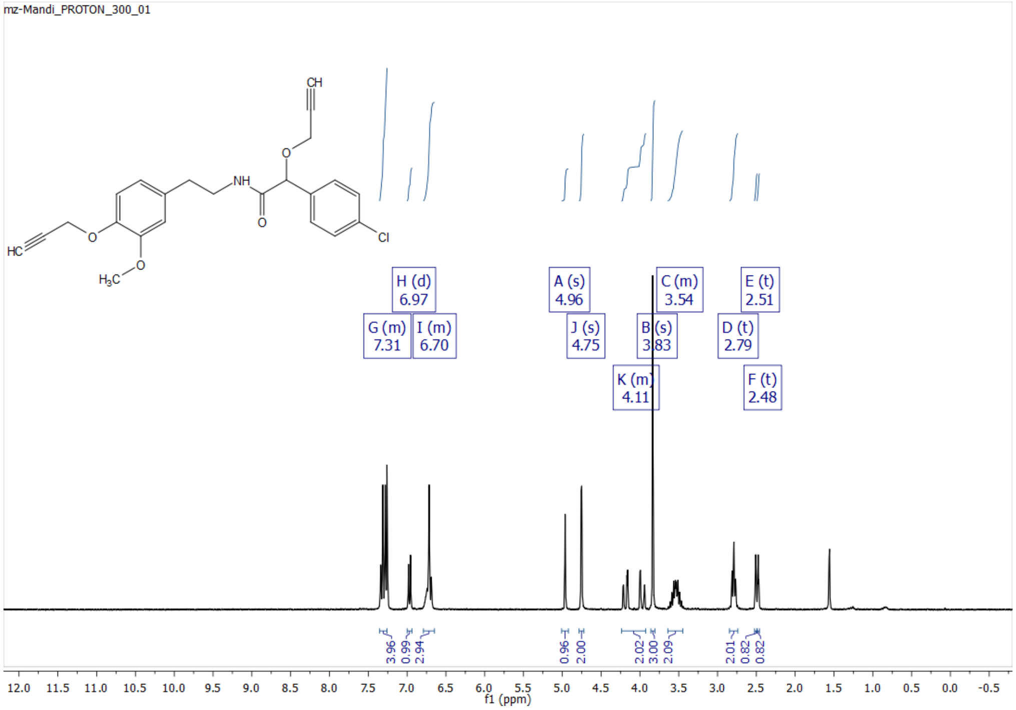

^{13}C NMR/APT

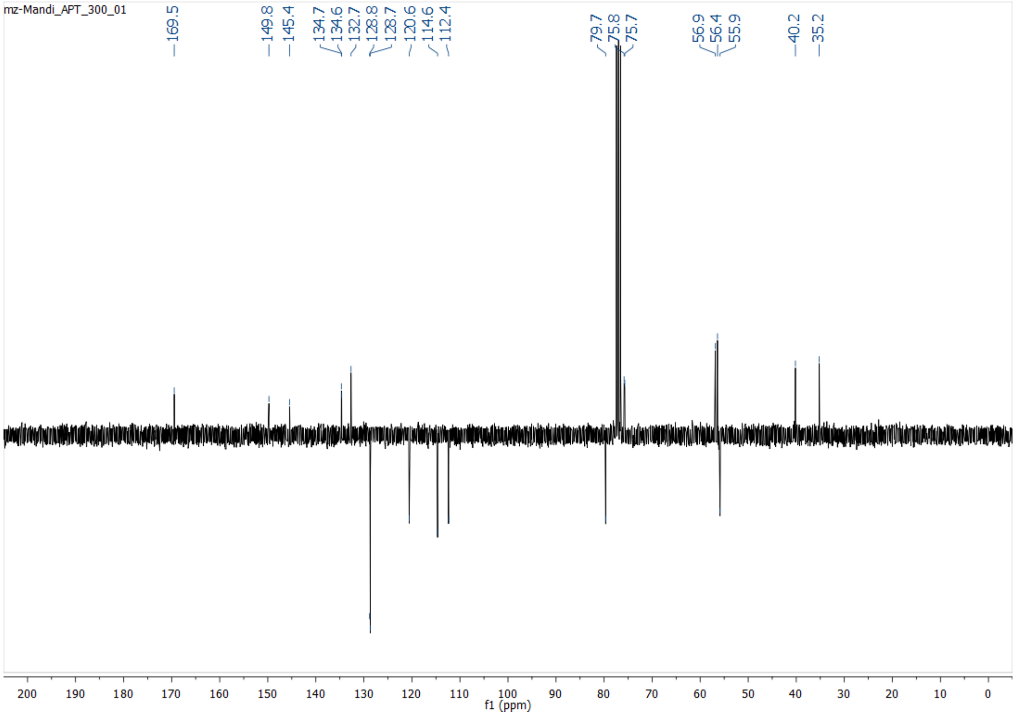

*ABA-AM (**4**)*

^{1}H NMR

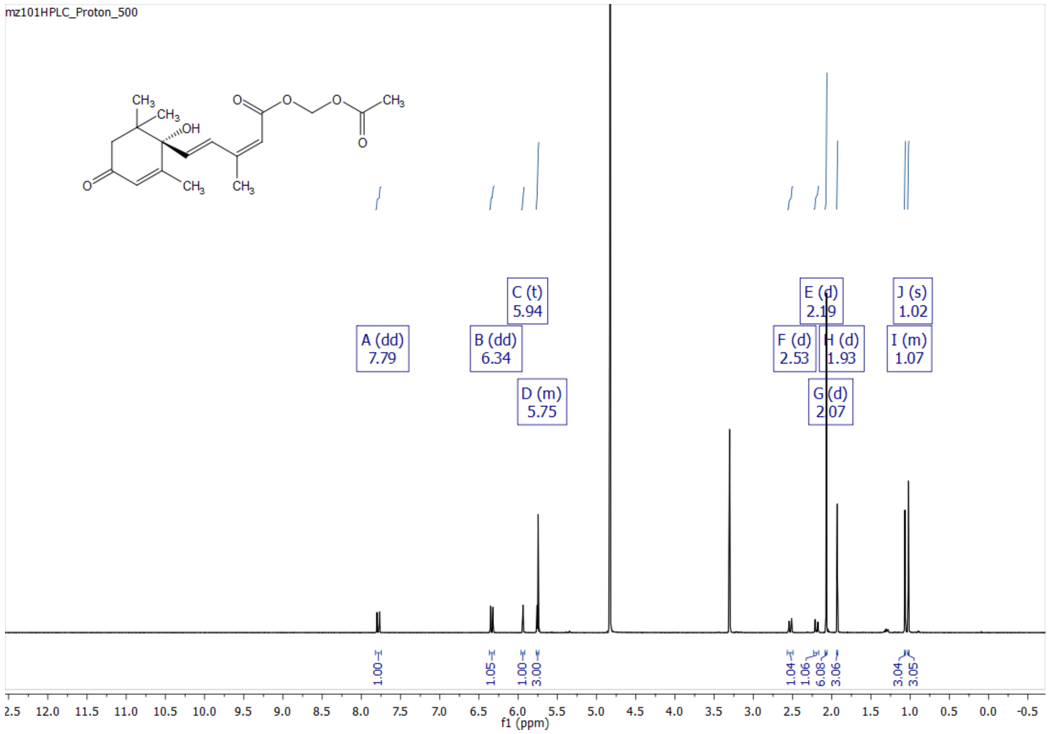

^{13}C NMR/APT

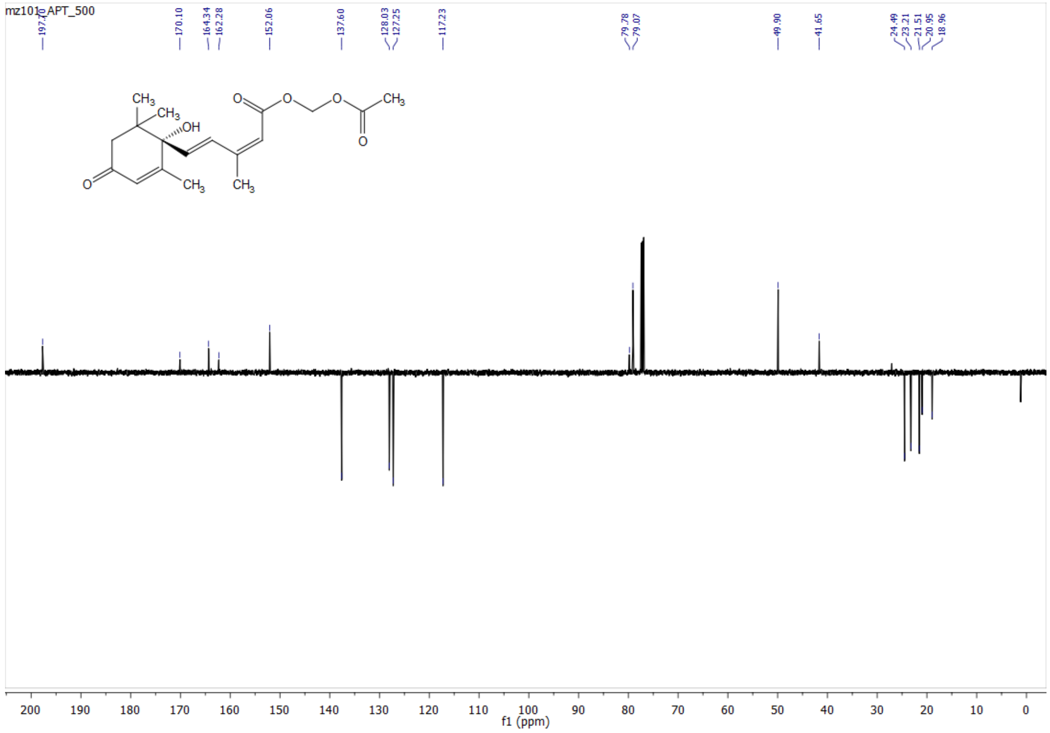

(p-Tolyl)prop-2-yn-1-ol

^{1}H NMR

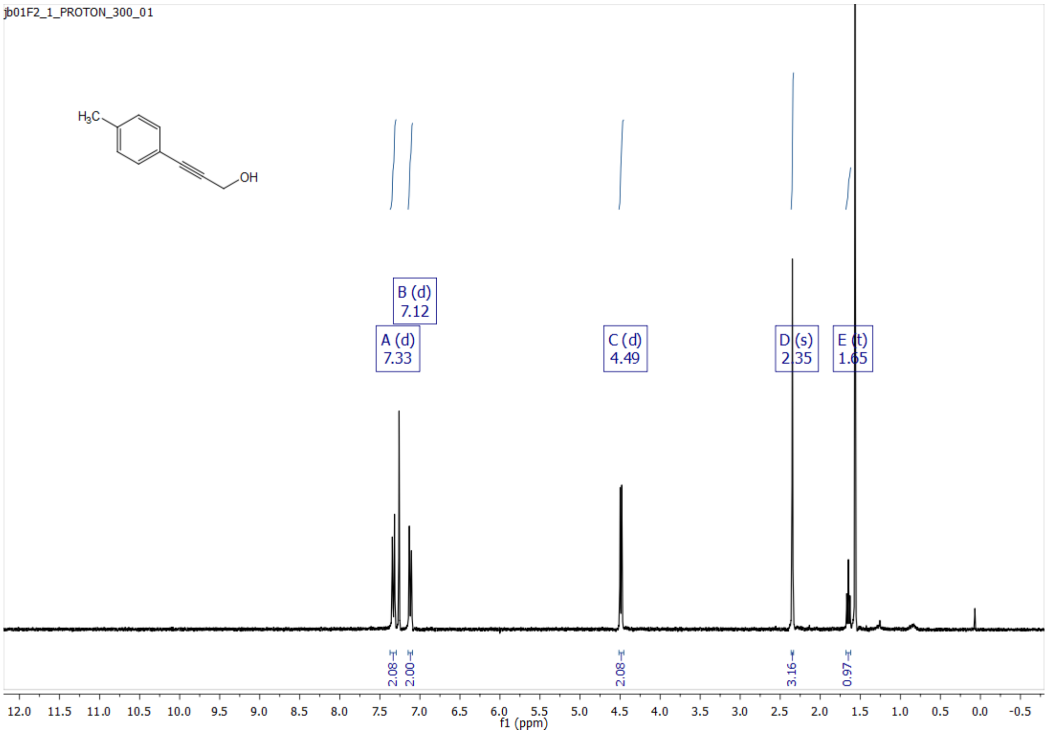

^{13}C NMR/APT

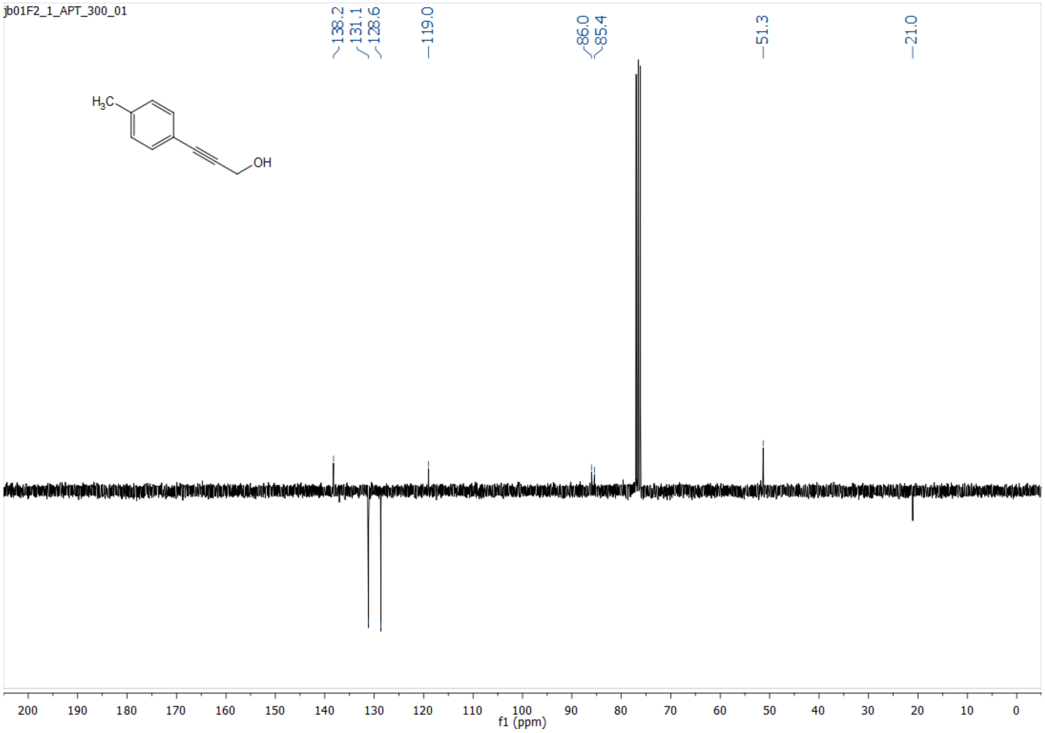

3-(p-Tolyl)prop-2-yn-1-yl 4-methylbenzolsulfonate

^{1}H NMR

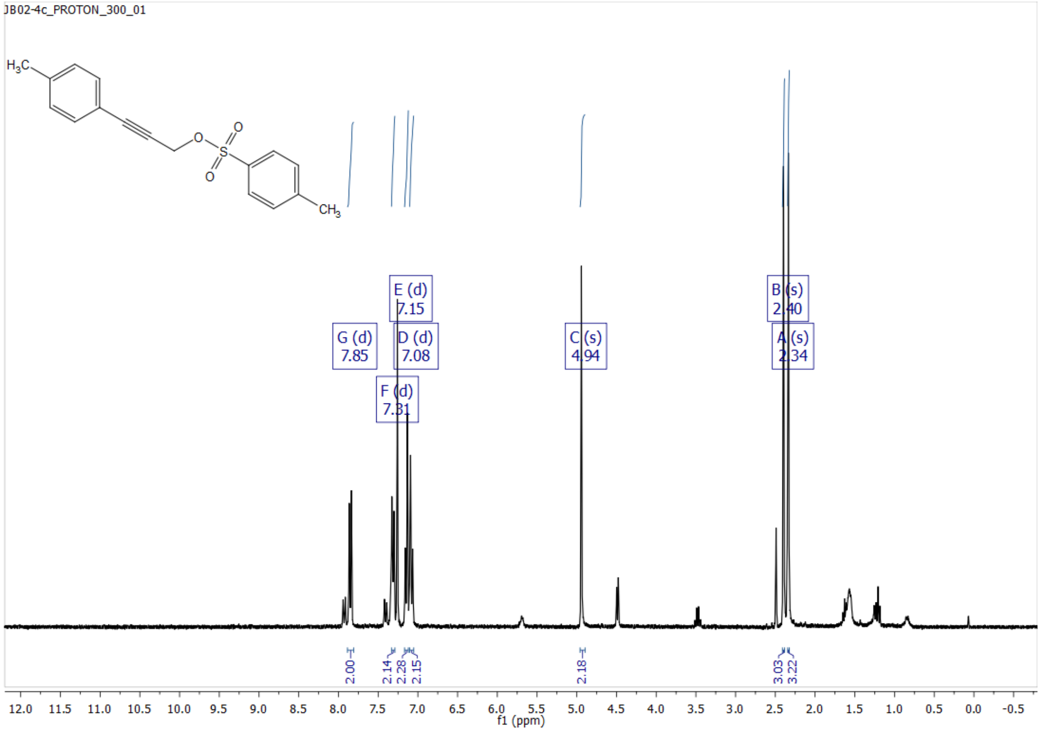

^{13}C NMR/APT

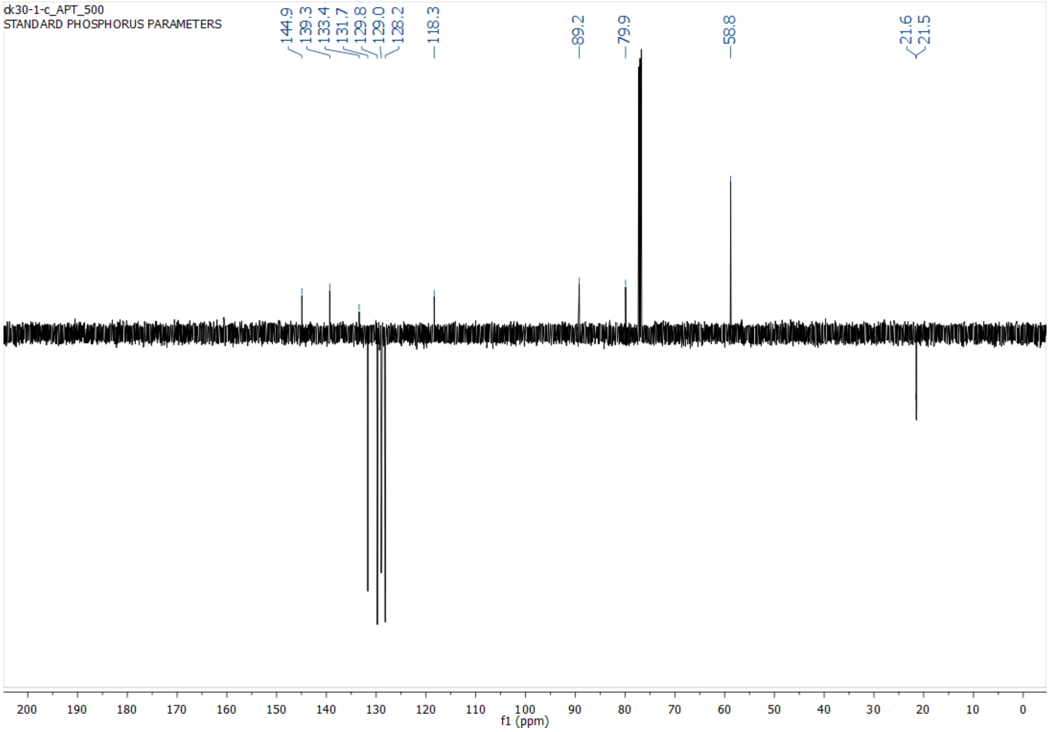

ABA-(S)-OH

^{1}H NMR

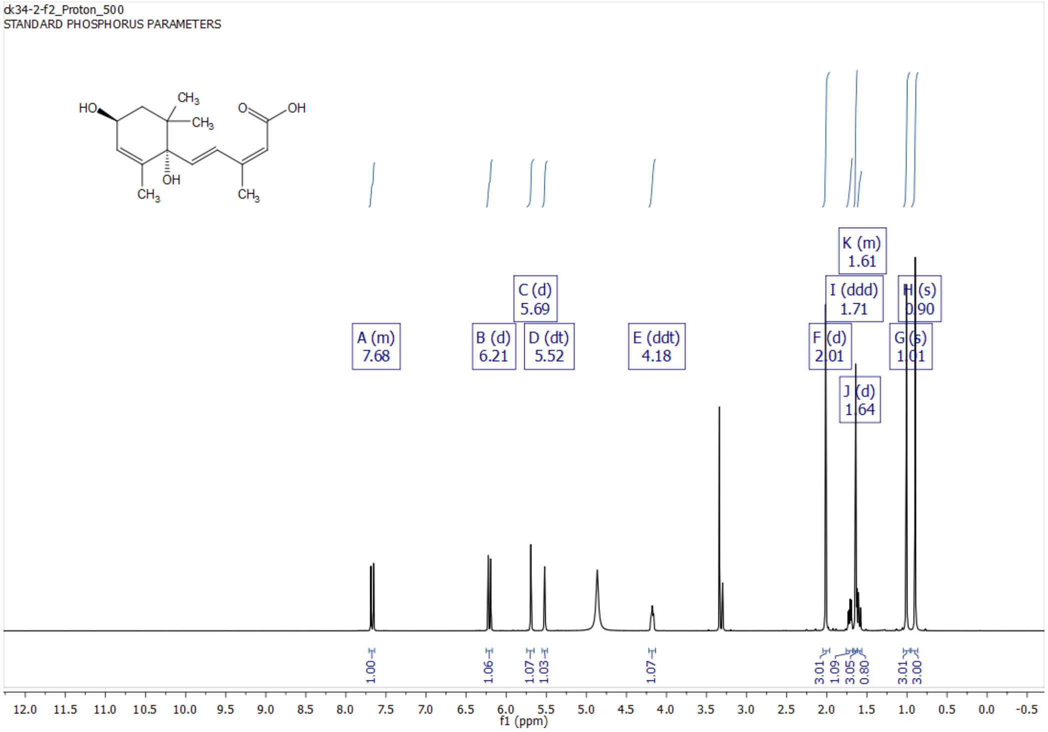

^{13}C NMR-APT

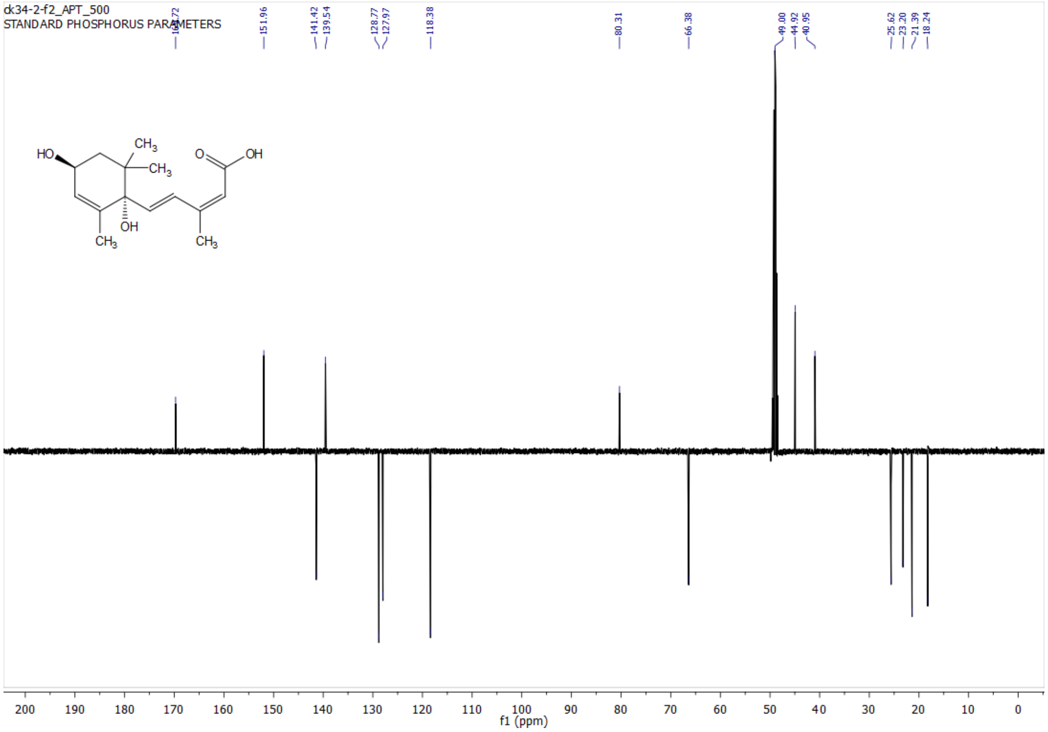

revABA (**7**)

^{1}H NMR

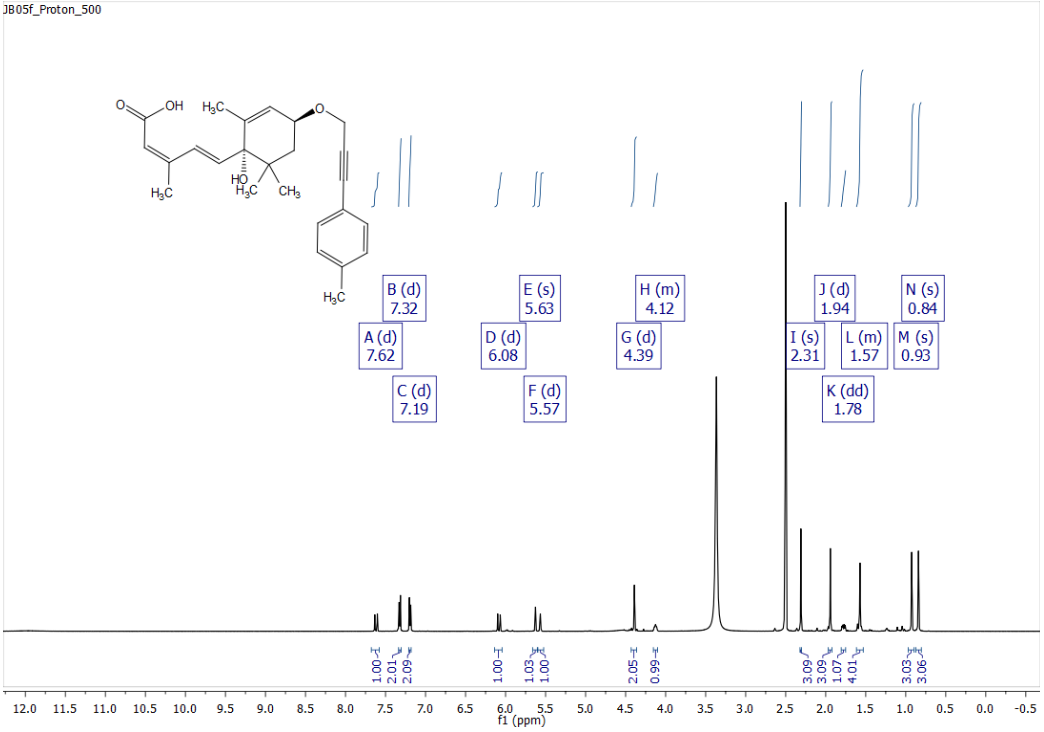

^{13}C NMR-APT

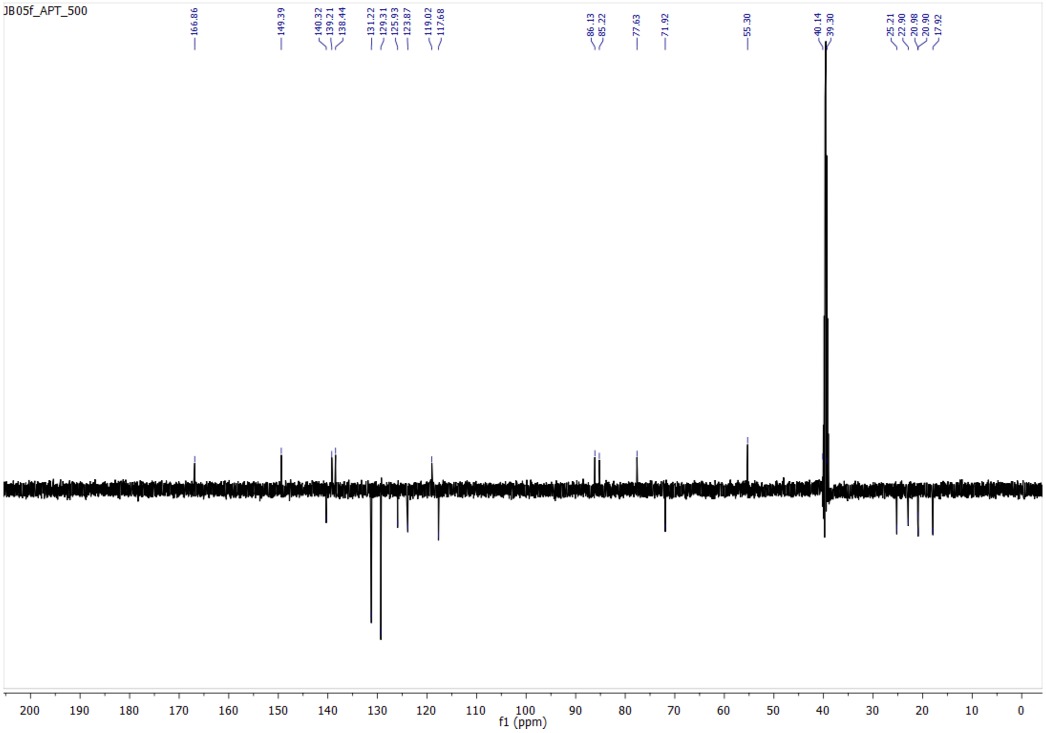

Supplement: Supplementary file 1 — Supplementary Tables 1–3, Figs. 1–13, References and Notes 1 and 2. [file 41589_2021_922_MOESM15_ESM.pdf]
