# Supplementary material for: Mandipropamid as a chemical inducer of proximity for in vivo applications
Source: Nat Chem Biol. 2021 Dec 21;18(1):64–9. doi: 10.1038/s41589-021-00922-3 (PMC8709788; doi:10.1038/s41589-021-00922-3)
Supplement: Supplementary file 2 — Reporting Summary [file 41589_2021_922_MOESM16_ESM.pdf]

nature research

Corresponding author(s): Richard Wombacher

Last updated by author(s): Jul 9, 2021

# Reporting Summary

Nature Research wishes to improve the reproducibility of the work that we publish. This form provides structure for consistency and transparency in reporting. For further information on Nature Research policies, see our Editorial Policies and the Editorial Policy Checklist.

## Statistics

For all statistical analyses, confirm that the following items are present in the figure legend, table legend, main text, or Methods section.

| n/a | Confirmed | |
|---|---|---|
| ☐ | ☒ | The exact sample size ($n$) for each experimental group/condition, given as a discrete number and unit of measurement |
| ☐ | ☒ | A statement on whether measurements were taken from distinct samples or whether the same sample was measured repeatedly |
| ☐ | ☒ | The statistical test(s) used AND whether they are one- or two-sided<br>*Only common tests should be described solely by name; describe more complex techniques in the Methods section.* |
| ☒ | ☐ | A description of all covariates tested |
| ☒ | ☐ | A description of any assumptions or corrections, such as tests of normality and adjustment for multiple comparisons |
| ☐ | ☒ | A full description of the statistical parameters including central tendency (e.g. means) or other basic estimates (e.g. regression coefficient) AND variation (e.g. standard deviation) or associated estimates of uncertainty (e.g. confidence intervals) |
| ☐ | ☒ | For null hypothesis testing, the test statistic (e.g. $F$, $t$, $r$) with confidence intervals, effect sizes, degrees of freedom and $P$ value noted<br>*Give P values as exact values whenever suitable.* |
| ☒ | ☐ | For Bayesian analysis, information on the choice of priors and Markov chain Monte Carlo settings |
| ☒ | ☐ | For hierarchical and complex designs, identification of the appropriate level for tests and full reporting of outcomes |
| ☐ | ☒ | Estimates of effect sizes (e.g. Cohen's $d$, Pearson's $r$), indicating how they were calculated |

*Our web collection on statistics for biologists contains articles on many of the points above.*

## Software and code

Policy information about availability of computer code

| | |
|---|---|
| Data collection | - Confocal microscopy: NIS-Elements AR Version 5.11.01 (Nikon)<br>- Epifluorescence microscopy: MicroManager Version 1.4.23. Described in Edelstein et al., J Biol Methods (2014).<br>- RSICS: Zen Version 2.1 (Zeiss)<br>- In vivo confocal microscopy: TCSNTV (Leica)<br>-Plate reader: Tecan Spark (Tecan) |
| Data analysis | - Fluorescence microscopy data: Imagej/Fiji Version 1.52p. Described in Schindelin et al., Nat. Methods (2012).<br>- Plotting and in-cell CIP kinetics: Custom Matlab code, MATLAB Release 2018a (MathWorks) as described in Methods.<br>- Mass spectrometry: ESI-Compass 1.3 Version 4.0 (Bruker).<br>- NMR: MestReNova Version 9.0.1 (Mestrelab Research).<br>- Unsupervised segmentation of fluorescence micrscopy images: Trainable Weka Segmentation. ImageJ plugin, version 3.2.33. Described in Arganda-Carreras et al. Bioinformatics (2017).<br>- Alignment of multispectral fluorescence microscopy images: Image stabilizer developed by Kang Li. Obtained via http://www.cs.cmu.edu/~kangli/code/Image_Stabilizer.html<br>- RSICS: Custom Matlab code, MATLAB Release 2015b as described in Methods, following published literature.<br>- Gene Expression: Custom Matlab code, MATLAB Release 2015b<br>- Split Protease: GraphPad Prism 9.1.2 |

For manuscripts utilizing custom algorithms or software that are central to the research but not yet described in published literature, software must be made available to editors and reviewers. We strongly encourage code deposition in a community repository (e.g. GitHub). See the Nature Research guidelines for submitting code & software for further information.

## Data

Policy information about availability of data

All manuscripts must include a data availability statement. This statement should provide the following information, where applicable:

- Accession codes, unique identifiers, or web links for publicly available datasets
- A list of figures that have associated raw data
- A description of any restrictions on data availability

The manuscript contains the following data availability statement:
"Raw data is available from the corresponding author upon reasonable request."

# Field-specific reporting

Please select the one below that is the best fit for your research. If you are not sure, read the appropriate sections before making your selection.

☒ Life sciences ☐ Behavioural & social sciences ☐ Ecological, evolutionary & environmental sciences

For a reference copy of the document with all sections, see nature.com/documents/nr-reporting-summary-flat.pdf

# Life sciences study design

All studies must disclose on these points even when the disclosure is negative.

| | |
|---|---|
| Sample size | No statistical methods were used to predetermine sample sizes. The number of cells/embryos assayed per condition and experiment was chosen such as to minimize the time samples (cells, embryos) were kept on the microscope. For all samples at least 2 repetitions were performed. |
| Data exclusions | In general, cells/embryos were selected for imaging based on the expression strength of transfected constructs. Cells/embryos with weak or strong expression not representative for population of transfected cells were not used for experiments.<br><br>- in-cell CIP kinetics: Cells where segmentation was not reliable were identified using the average mitochondrial 561 nm signal over time and excluded from analysis.The percentage of discarded cells per condition is stated in supplementary table 2.<br>- RSICS experiments: Measurements with average expression levels differing by more than a factor of 3 between the three fluorophore species were excluded as stated in the Methods section.<br>- Protein shuttling: No cells were excluded from analysis. |
| Replication | All experimental findings were reproducible in at least two independent experiments. The specific number of experiments and replicates for each experiment is stated in the corresponding figure caption. |
| Randomization | n/a - no allocation into experimental groups was performed. |
| Blinding | n/a - no allocation into experimental groups was performed. All quantifications are based on automated analysis workflows without user interference. |

# Reporting for specific materials, systems and methods

We require information from authors about some types of materials, experimental systems and methods used in many studies. Here, indicate whether each material, system or method listed is relevant to your study. If you are not sure if a list item applies to your research, read the appropriate section before selecting a response.

## Materials & experimental systems

| n/a | Involved in the study | |
|---|---|---|
| ☒ | ☐ | Antibodies |
| ☐ | ☒ | Eukaryotic cell lines |
| ☒ | ☐ | Palaeontology and archaeology |
| ☐ | ☒ | Animals and other organisms |
| ☒ | ☐ | Human research participants |
| ☒ | ☐ | Clinical data |
| ☒ | ☐ | Dual use research of concern |

## Methods

| n/a | Involved in the study | |
|---|---|---|
| ☒ | ☐ | ChIP-seq |
| ☒ | ☐ | Flow cytometry |
| ☒ | ☐ | MRI-based neuroimaging |

# Eukaryotic cell lines

Policy information about cell lines

| | |
|---|---|
| Cell line source(s) | HEK293T and COS-7 cells were obtained from ATCC.<br>REF cells expressing YFP-Paxilin were obtained from Ada Cavalcanti-Adam (Max-Planck Institute for Medical Research).<br>HeLa cells expressing LifeAct-eGFP-HaloTag were obtained from Jacob Piehler (Universitaet Osnabrueck). |
| Authentication | All Cell lines were used without authentication. |
| Mycoplasma contamination | None of the cell lines used was tested for Mycoplasma contamination. |
| Commonly misidentified lines (See ICLAC register) | HEK293T cell lines were used due to their suitability in visualizing plasma membrane-localized reporters. |

# Animals and other organisms

Policy information about studies involving animals; ARRIVE guidelines recommended for reporting animal research

| | |
|---|---|
| Laboratory animals | Danio rerio, AB2O2 wildtype, larvae 3-5 days post fertilization |
| Wild animals | n/a - no wild animals were used. |
| Field-collected samples | n/a - no wild animals were used. |
| Ethics oversight | Regierungspräsidium Karlsruhe, Germany, 35-9185.64/BH KIT |

Note that full information on the approval of the study protocol must also be provided in the manuscript.
