# Supplementary figures and images for: Mandipropamid as a chemical inducer of proximity for in vivo applications
Source: Nat Chem Biol. 2021 Dec 21;18(1):64–9. doi: 10.1038/s41589-021-00922-3 (PMC8709788; doi:10.1038/s41589-021-00922-3)

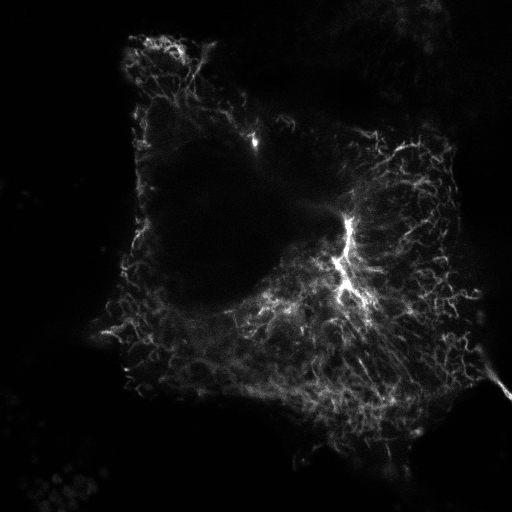

Supplement: Source Data Fig. 4 — Images for Pearson’s coefficient determination, ROIs and calculated Pearson’s coefficients. [file 41589_2021_922_MOESM22_ESM.zip › Figure_04/Figure_04b/exp01_cell07_t0.tif]

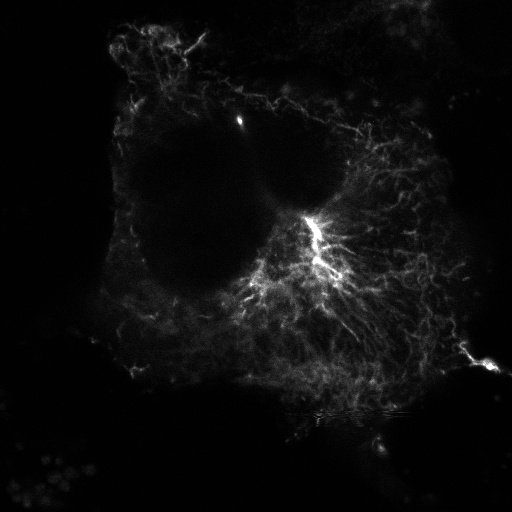

Supplement: Source Data Fig. 4 — Images for Pearson’s coefficient determination, ROIs and calculated Pearson’s coefficients. [file 41589_2021_922_MOESM22_ESM.zip › Figure_04/Figure_04b/exp01_cell07_t1.tif]

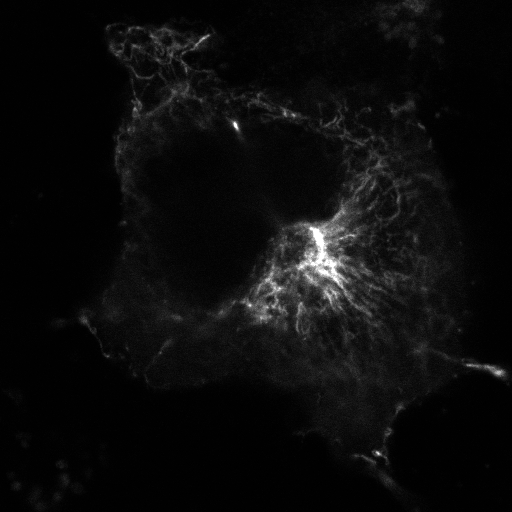

Supplement: Source Data Fig. 4 — Images for Pearson’s coefficient determination, ROIs and calculated Pearson’s coefficients. [file 41589_2021_922_MOESM22_ESM.zip › Figure_04/Figure_04b/exp01_cell07_t2.tif]

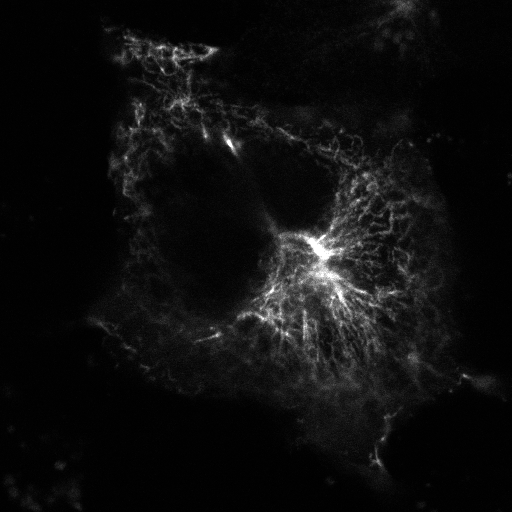

Supplement: Source Data Fig. 4 — Images for Pearson’s coefficient determination, ROIs and calculated Pearson’s coefficients. [file 41589_2021_922_MOESM22_ESM.zip › Figure_04/Figure_04b/exp01_cell07_t3.tif]

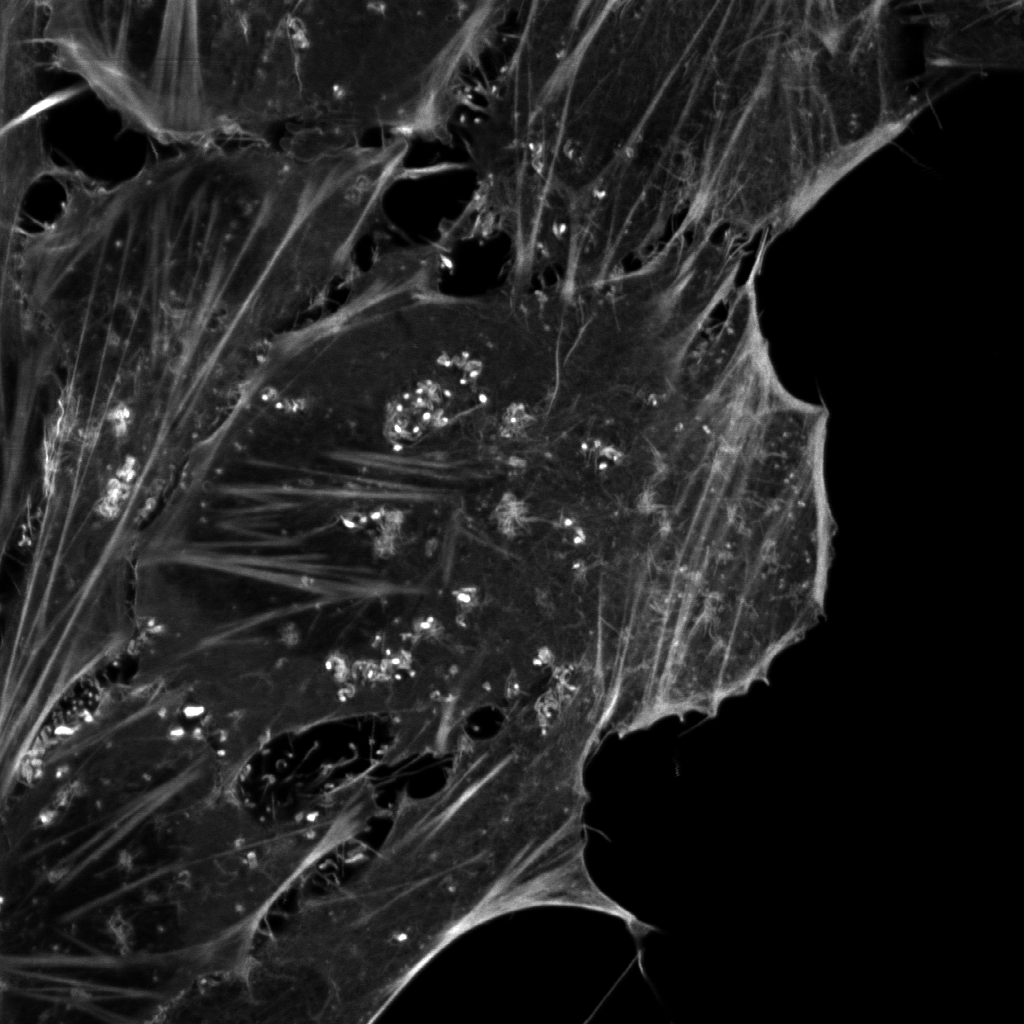

Supplement: Source Data Extended Data Fig. 6 — Images for line profiles, ROIs and intensity line profiles. [file 41589_2021_922_MOESM24_ESM.zip › ExtendedDataFigure_06/ExtendedDataFigure_04b/exp01_cell04.tif]

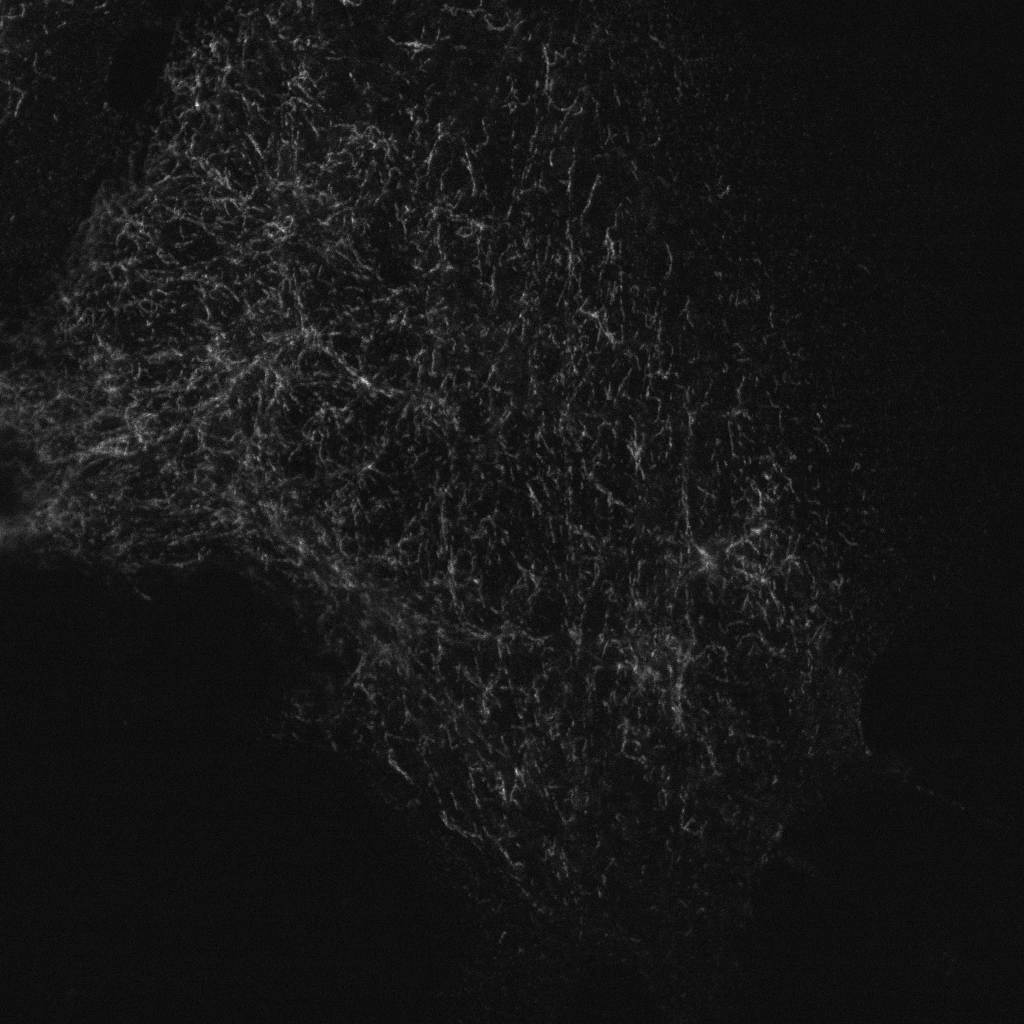

Supplement: Source Data Extended Data Fig. 8 — Images for Pearson’s coefficient determination, ROIs and calculated Pearson’s coefficients. [file 41589_2021_922_MOESM25_ESM.zip › ExtendedDataFigure_08/ExtendedDataFigure_08a/exp01_cell02_t0.tif]

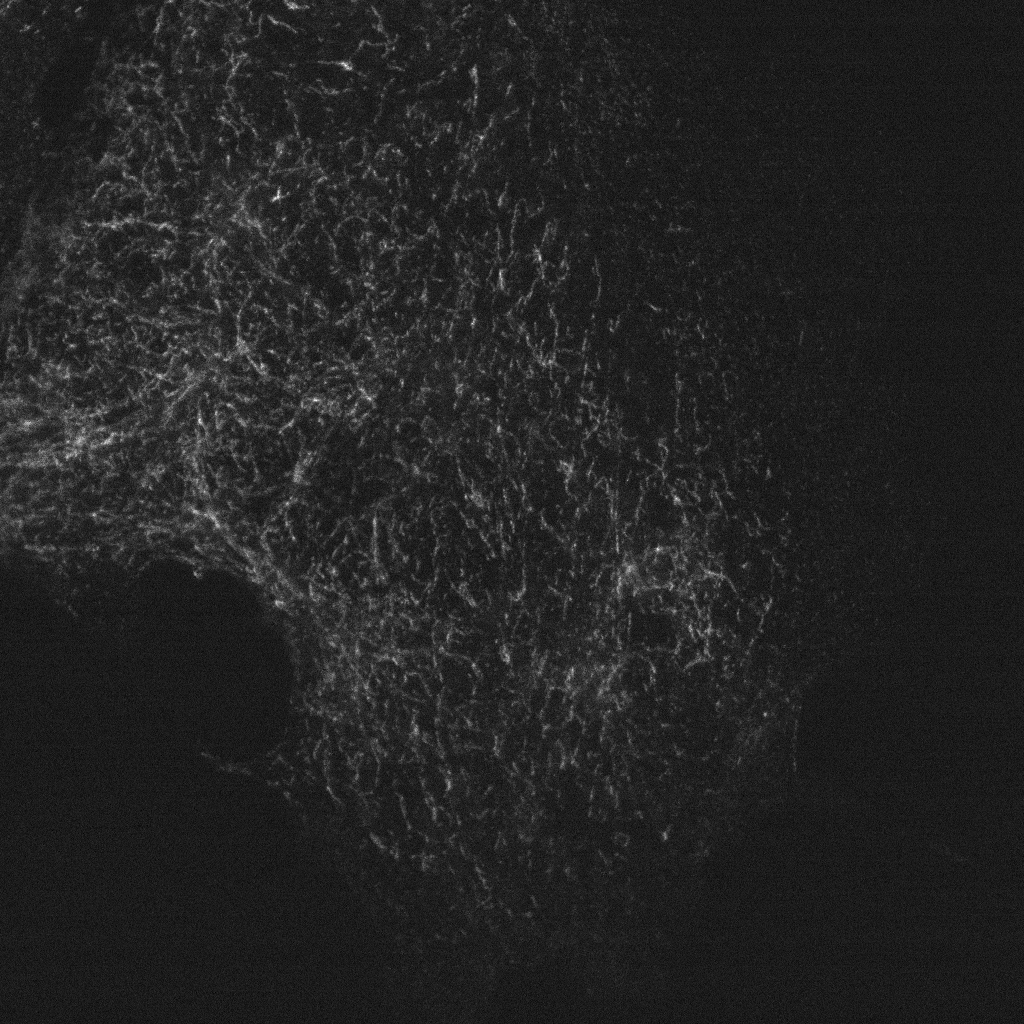

Supplement: Source Data Extended Data Fig. 8 — Images for Pearson’s coefficient determination, ROIs and calculated Pearson’s coefficients. [file 41589_2021_922_MOESM25_ESM.zip › ExtendedDataFigure_08/ExtendedDataFigure_08a/exp01_cell02_t1.tif]

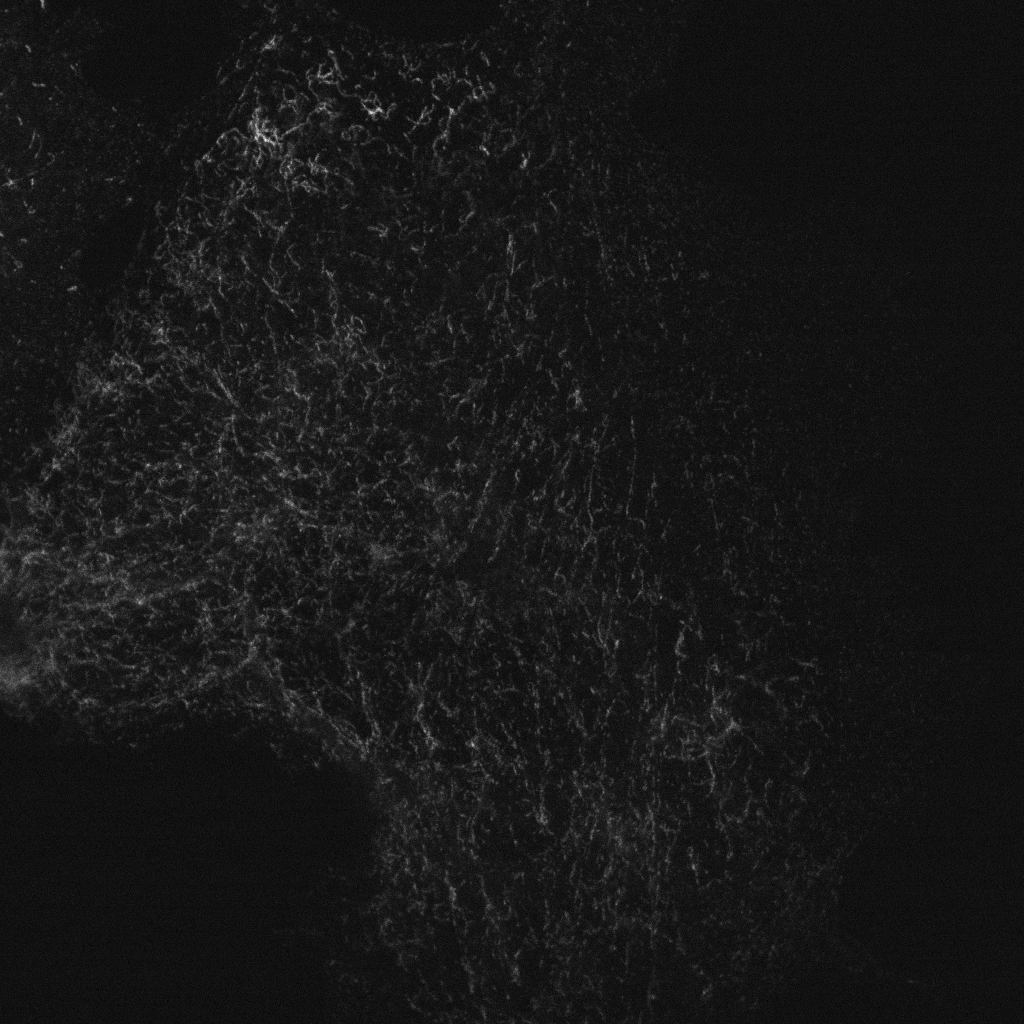

Supplement: Source Data Extended Data Fig. 8 — Images for Pearson’s coefficient determination, ROIs and calculated Pearson’s coefficients. [file 41589_2021_922_MOESM25_ESM.zip › ExtendedDataFigure_08/ExtendedDataFigure_08a/exp01_cell02_t2.tif]

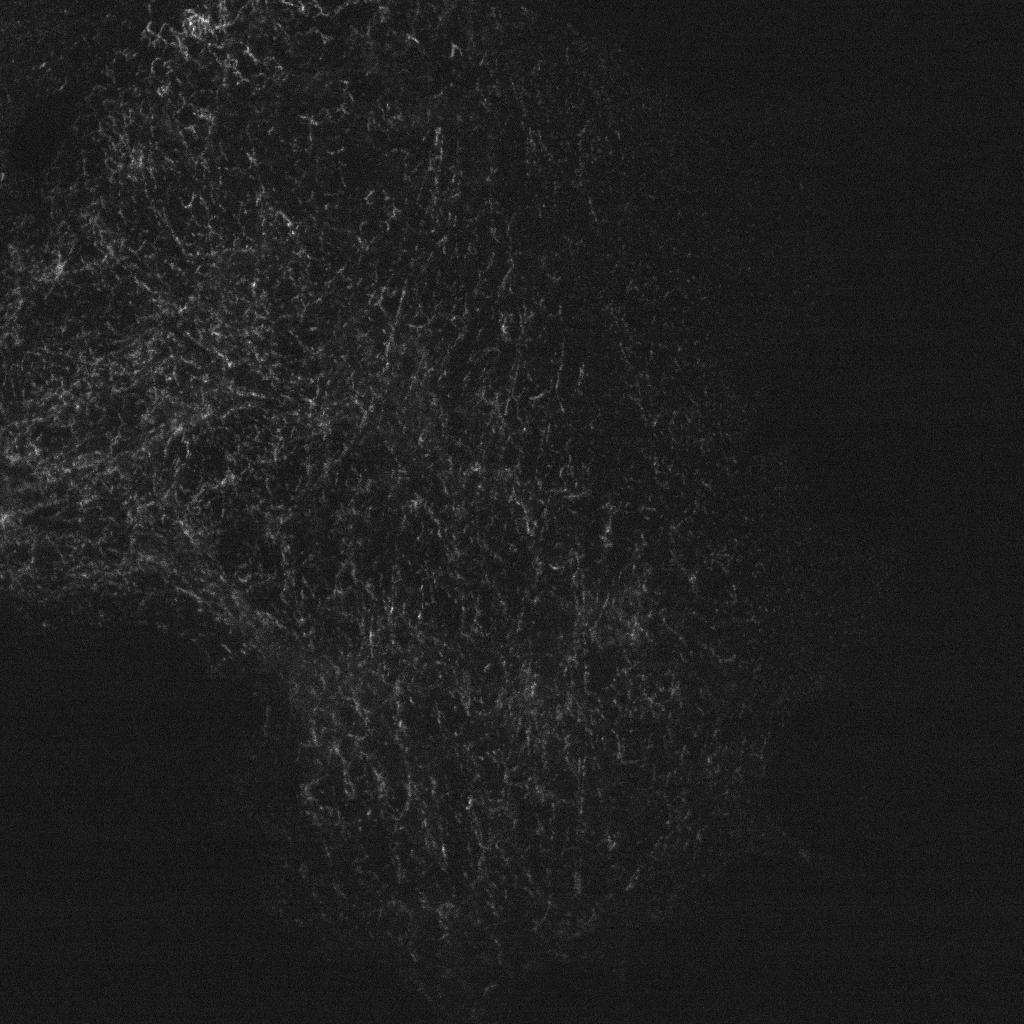

Supplement: Source Data Extended Data Fig. 8 — Images for Pearson’s coefficient determination, ROIs and calculated Pearson’s coefficients. [file 41589_2021_922_MOESM25_ESM.zip › ExtendedDataFigure_08/ExtendedDataFigure_08a/exp01_cell02_t3.tif]
